# Tri‐ and Tetranuclear Metal‐String Complexes with Metallophilic d^10^–d^10^ Interactions

**DOI:** 10.1002/chem.201904106

**Published:** 2019-11-26

**Authors:** Marian Olaru, Julius F. Kögel, Risa Aoki, Ryota Sakamoto, Hiroshi Nishihara, Enno Lork, Stefan Mebs, Matthias Vogt, Jens Beckmann

**Affiliations:** ^1^ Institut für Anorganische Chemie und Kristallographie Universität Bremen Leobener Strasse 7 28359 Bremen Germany; ^2^ Department of Chemistry Graduate School of Science The University of Tokyo 7-3-1, Hongo Bunkyo-ku Tokyo 113-0033 Japan; ^3^ JST-PRESTO 4-1-8, Honcho, Kawaguchi Saitama 332-0012 Japan; ^4^ Institut für Experimentalphysik Freie Universität Berlin Arnimallee 14 14195 Berlin Germany

**Keywords:** gold, ligand design, mercury, metallophilic interactions, metallopincer

## Abstract

The reaction of 2,6‐F_2_C_6_H_3_SiMe_3_ with Ph_2_PLi provided 2,6‐(Ph_2_P)_2_C_6_H_3_SiMe_3_ (**1**), which can be regarded as precursor for the novel anionic tridentate ligand [2,6‐(Ph_2_P)_2_C_6_H_3_]^−^ (*PCP*)^−^. The reaction of **1** with [AuCl(tht)] (tht=tetrahydrothiophene) afforded 2,6‐(Ph_2_PAuCl)_2_C_6_H_3_SiMe_3_ (**2**). The subsequent reaction of **2** with CsF proceeded with elimination of Me_3_SiF and yielded the neutral tetranuclear complex *linear*‐[Au_4_Cl_2_(*PCP*)_2_] (**3**) comprising a string‐like arrangement of four Au atoms. Upon chloride abstraction from **3** with NaBAr^F^_4_ (Ar^F^=3,5‐(CF_3_)_2_C_6_H_3_) in the presence of tht, the formation of the dicationic tetranuclear complex *linear*‐[Au_4_(*PCP*)_2_(tht)_2_](BAr^F^_4_)_2_ (**4**) was observed, in which the string‐like structural motif is retained. Irradiation of **4** with UV light triggered a facile rearrangement in solution giving rise to the dicationic tetranuclear complex *cyclo*‐[Au_4_(*PCP*)_2_(tht)_2_](BAr^F^_4_) (**5**), which comprises a rhomboidal motif of four Au atoms. In **3**–**5**, the Au atoms are associated by a number of significant aurophilic interactions. The atom‐economic and selective reaction of **3** with HgCl_2_ yielded the neutral trinuclear bimetallic complex [HgAu_2_Cl_3_(*PCP*)] (**6**) comprising significant metallophilic interactions between the Au and Hg atoms. Therefore, **6** may be also regarded as a metallopincer complex [ClHg(*AuCAu*)] between Hg^II^ and the anionic tridentate ligand [2,6‐(Ph_2_PAuCl)_2_C_6_H_3_]^−^ (*AuCAu*)^−^ containing a central carbanionic binding site and two “gold‐arms” contributing pincer‐type chelation trough metallophilic interactions. Compounds **1**–**6** were characterized experimentally by multinuclear NMR spectroscopy and X‐ray crystallography and computationally using a set of real‐space bond indicators (RSBIs) derived from electron density (ED) methods including Atoms In Molecules (AIM), the Electron Localizability Indicator (ELI‐D) as well as the Non‐Covalent Interaction (NCI) Index.

## Graphical abstract

**Heavy metal interactions**: The synthesis and characterization of several tetranuclear gold complexes and of a trinuclear bimetallic (metallopincer) complex featuring metallophilic interactions is described in this work.

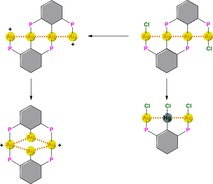

## Introduction

**Scheme 1:**
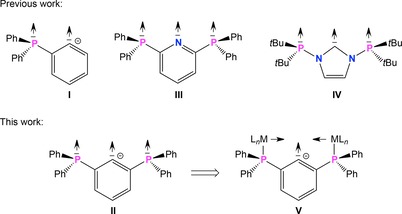
Multidentate ligands to support transition metal complexes.

Metallophilic interactions are structurally directing, attractive forces between two or more closed‐shell metal ions that prefer low coordination numbers. From a quantum‐mechanical point of view, metallophilic interactions are mostly dispersive forces that are significantly enhanced by relativistic effects.^1^ Given that relativistic effects dramatically increase for the post‐lanthanide elements and reach a maximum for gold in the sixth period, linearly coordinated, 12 valence‐electron complexes of Au^I^ and Hg^II^ both having a 5d^10^6s^0^ electron configuration are the most prominent closed‐shell metal species showing metallophilic interactions. In addition to pure aurophilic^2^ and mercurophilic^3^ interactions, an increasing number of heteronuclear metal–metal contacts,^4^ for example, of the Au⋅⋅⋅Hg type,^5^, ^6^ have been observed in recent years. Metallophilic interactions are often related to interesting photophysical phenomena such as luminescence. Since the first publication on a photoluminescent gold complex by the group of Dori in 1970,^7^ numerous examples of photoluminescence based on aurophilic interactions have been reported.^8^ Given that bond energies associated with metallophilic interactions rarely exceed 50 kJ mol^−l^, multidentate substituents or ligands often play a critical role to support multinuclear complexes in which the metal atoms are fixed in close proximity to each other. In this regard, the 2‐diphenylphosphinophenyl ligand (**I**) and derivatives thereof have been frequently used to prepare a number of dinuclear Au^I^⋅⋅⋅Au^I^ complexes, which were for instance the starting materials for the preparation of interesting Au^II^–Au^II^ complexes through oxidative‐addition reactions (Scheme 1).^9^

In this work we report on tri‐ and tetranuclear Au^I^ and Hg^II^ complexes derived from the related 2,6‐bis(diphenylphosphino)phenyl ligand (**II**, Scheme 1), which were prepared through a novel synthetic route using a trimethylsilyl substituent as protecting group. The 2,6‐bis(diphenylphosphino)phenyl ligand **II** is isoelectronic to the 2,6‐bis(diphenylphosphino)pyridine ligand (**III**),^10^ which has been utilized previously for the preparation of interesting complexes including some metal‐string complexes, which hold promise as molecular wire materials (Scheme 1).^11^ The 2,6‐bis(diphenylphosphino)phenyl ligand **II** is also closely related to the diphosphanyl NHC ligand **IV** and related ligand systems, which were introduced recently (Scheme 1) and were shown to stabilize poly‐homo and heterometallic complexes showing metallophilic interactions.^12^

## Results and Discussion

### Synthetic aspects

**Scheme 2:**
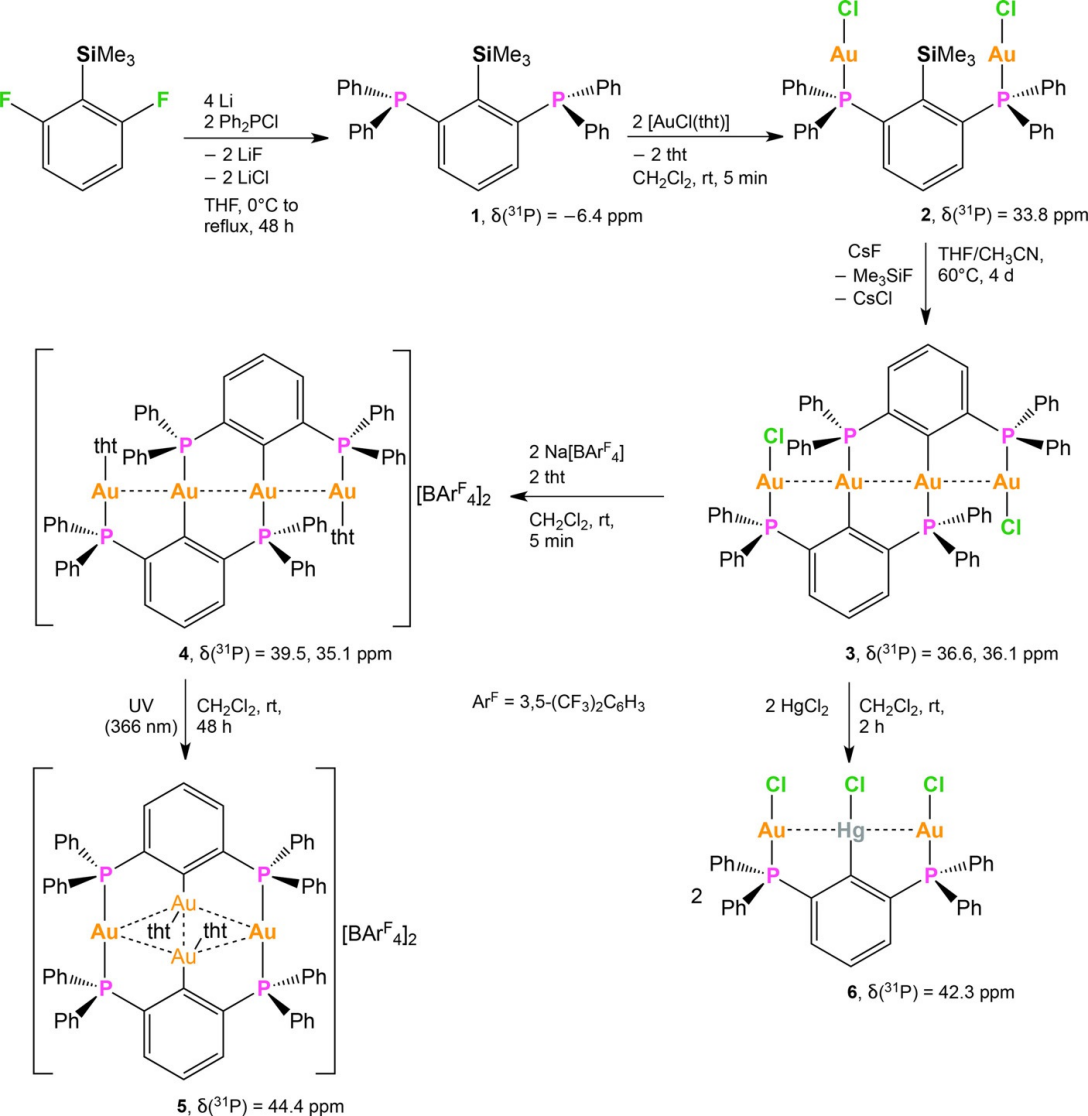
Synthesis and ^31^P NMR chemical shifts in ppm of **1**–**6**.

The reaction of 2,6‐F_2_C_6_H_3_SiMe_3_ with Ph_2_PLi provided 2,6‐(Ph_2_P)_2_C_6_H_3_SiMe_3_ (**1**) as a colorless solid in 54 % yield (Scheme 2). The ^31^P{^1^H} NMR spectrum of **1** dissolved in CDCl_3_ shows a characteristic singlet at *δ*=−6.4 ppm. The corresponding ^29^Si NMR resonance is observed as a triplet (^4^ *J*_Si–P_=7.8 Hz) in the ^29^Si{^1^H} NMR spectrum at *δ*=−3.0 ppm. The complex [2,6‐(Ph_2_PAuCl)_2_C_6_H_3_SiMe_3_] (**2**) was readily prepared through the reaction of **1** with two equivalents of [AuCl(tht)] (Scheme 2). The reaction occurred instantaneously at ambient temperature when both reactants were suspended in dichloromethane. Subsequent crystallization gave **2** as colorless crystals in 90 % yield. The coordination of the two P atoms to Au gives rise to a significant shift of the ^31^P NMR resonance to higher frequencies than **1**. The ^31^P{^1^H} NMR spectrum of **2** dissolved in [D_6_]DMSO reveals a singlet at *δ*=33.8 ppm for two chemically equivalent ^31^P nuclei. Note that the poor solubility of **2**, even in DMSO, precluded the acquisition of a ^29^Si{^1^H} NMR spectrum with sufficient signal intensity. However, the ^1^H NMR resonance associated with the (C*H*_3_)_3_Si group is observed at *δ*=0.58 ppm. Heating a mixture of **1**, [Au(tht)Cl], and anhydrous CsF as a suspension in THF/CH_3_CN (1:1) at 60 °C proceeded with elimination of Me_3_SiF and gave rise to the formation of the neutral tetranuclear complex *linear*‐[Au_4_Cl_2_(*PCP*)_2_] (**3**, *PCP*=2,6‐(Ph_2_P)_2_C_6_H_3_) that was obtained as yellow prisms in 60 % yield after recrystallisation from CH_2_Cl_2_/*n*‐hexane (Scheme 2). Inspection of the ^31^P{^1^H} NMR spectrum of **3** in CD_2_Cl_2_ shows two ^31^P NMR singlet resonances at *δ*=36.6 and 36.0 ppm associated with two sets of two chemically inequivalent phosphorus nuclei. That is, two Ph_2_P moieties coordinate to Au atoms with Cl^−^ ligands in mutual *trans*‐position (inorganic coordination site) and the remaining two Ph_2_P donors coordinate to the two carbon‐bound Au atoms in mutual *trans*‐position (organometallic coordination site). The ^29^Si{^1^H} NMR spectrum shows no signal indicating the loss of the Me_3_Si group in complex **3**. Coherently, no resonance corresponding to the C*H*_3_ group of the (C*H*_3_)_3_Si group is observed in the ^1^H NMR spectrum. Moreover, when the reaction was monitored in situ in a closed NMR tube, the formation of Me_3_SiF was observed indicated by the characteristic multiplet resonance in the ^19^F NMR spectrum at −159.6 ppm.^13^ Chloride abstraction from complex **3** employing NaBAr^F^_4_ (Ar^F^=3,5‐(CF_3_)_2_C_6_H_3_) in the presence of tht readily gave the dicationic tetranuclear complex *linear*‐[Au_4_(*PCP*)_2_(tht)_2_](BAr^F^_4_)_2_ (**4**) with full conversion. Complex **4** is light sensitive and consequently, the reaction was performed in the dark. With respect to **3**, the ^31^P{^1^H} NMR spectrum of **4** similarly reveals two sets of characteristic singlet resonances at *δ*=39.5 and 35.1 ppm, each associated with two chemically inequivalent ^31^P nuclei. One set resides in the internal organometallic coordination site and one set remains in the terminal inorganic coordination site. When the reaction mixture was subsequently exposed to UV light (*λ*_max_=366 nm), an rearrangement and thus quantitative formation of the dicationic tetranuclear complex *cyclo*‐[Au_4_(*PCP*)_2_(tht)_2_](BAr^F^_4_) (**5**) was observed. The rearrangement coincides with an increase in symmetry as signaled by the presence of a singlet resonance at *δ*=44.4 ppm in the ^31^P{^1^H} NMR spectrum (CD_2_Cl_2_). All ^31^P nuclei in **5** reside in an indistinguishable chemical environment with all Ph_2_P donors in mutual *cis*‐position coordinated to a terminal Au atom. Complex **5** was isolated as colorless crystals after recrystallization from CH_2_Cl_2_/petroleum ether. Noteworthy, complex **5** is stable towards moisture and air and only decomposes above 218 °C. The transmetalation of organogold compounds with Cu12c or Hg^14^ salts has been previously reported. The atom‐economic reaction of **3** with HgCl_2_ in CH_2_Cl_2_ proceeded with disaggregation of the tetranuclear complex and formation of Hg−C and Au−Cl bonds and produced the neutral trinuclear complex [HgAu_2_Cl_3_(*PCP*)] (**6**) in 96 % yield (Scheme 2). A ^199^Hg{^1^H} NMR spectrum of **6** was recorded in CD_2_Cl_2_. The observed triplet resonance at *δ*=−841.5 ppm shows a significant Hg−P coupling with a ^3^ *J*_Hg–P_ coupling constant of 327 Hz. Conversely, the ^31^P{^1^H} NMR spectrum has a singlet resonance at *δ*=42.3 ppm, signifying two Ph_2_P moieties with identical chemical environment, with ^199^Hg satellites exhibiting a ^3^ *J*_P–Hg_ coupling constant of 327 Hz.

### Molecular structures

**Figure 1:**
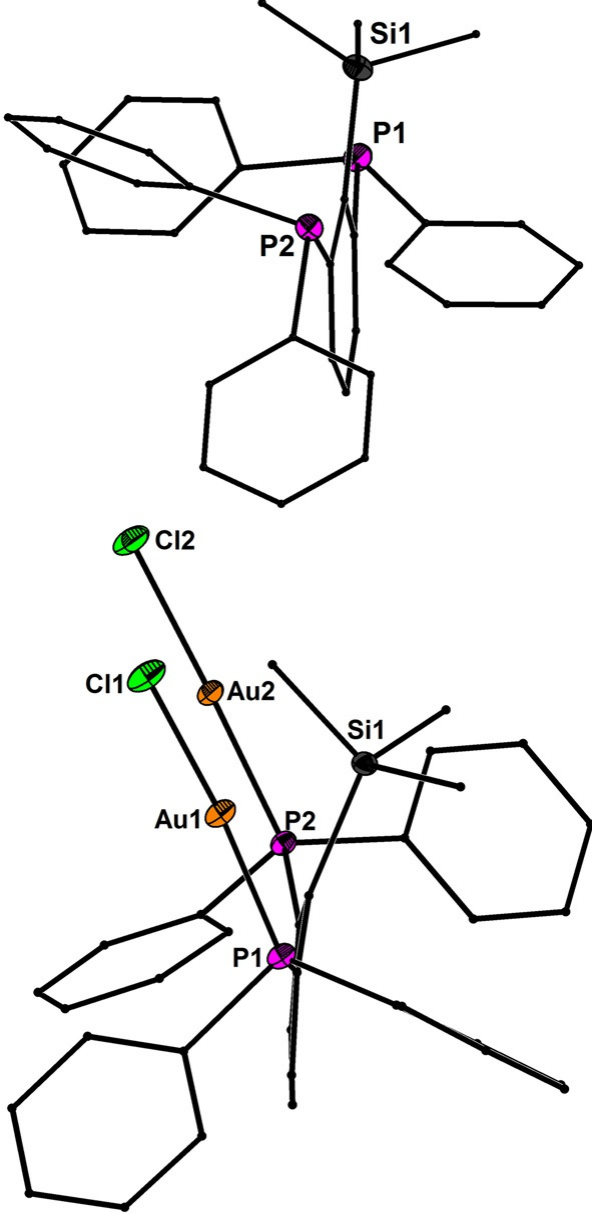
Molecular structures of **1** (up) and **2** (down) showing 50 % probability ellipsoids and the crystallographic numbering Scheme. Selected bond length of **1** [Å]: Si1−C10 1.926(1). Selected bond lengths of **2** [Å]: Si1−C10 1.944(6), Au1−P1 2.227(2), Au2−P2 2.228(2), Au1−Cl1 2.294(2), Au2−Cl2 2.280(2).

**Figure 2:**
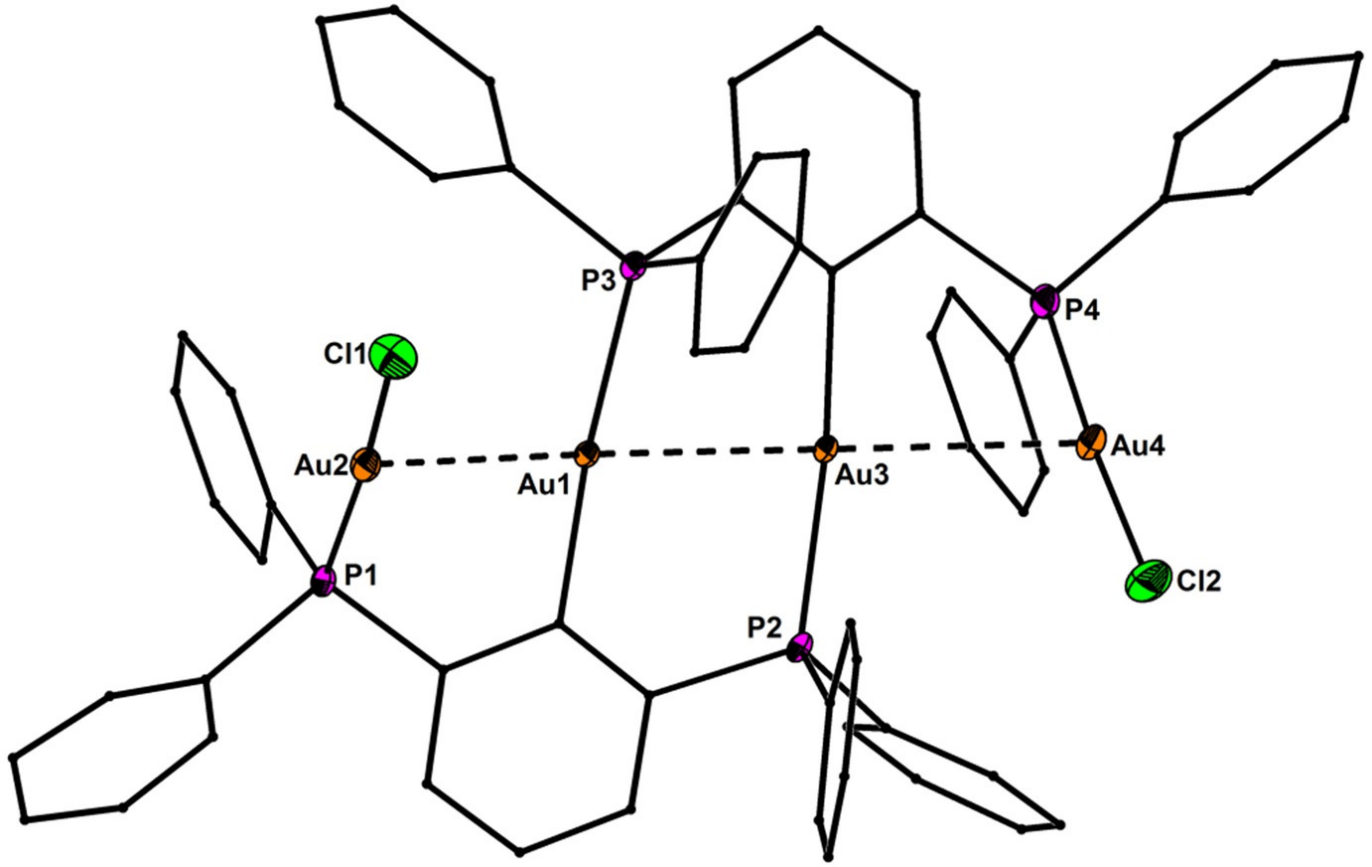
Molecular structure of **3** showing 50 % probability ellipsoids and the crystallographic numbering Scheme. Selected bond lengths [Å]: Au1−C10 2.063(7), Au3−C60 2.064(7), Au1−P3 2.302(2), Au2−P1 2.239(2), Au3−P2 2.305(2), Au4−P4 2.236(2), Au2−Cl1 2.295(2), Au4−Cl2 2.287(2), Au1−Au2 2.9890(4), Au1−Au3 2.8746(4), Au3−Au4 3.0567(4).

**Figure 3:**
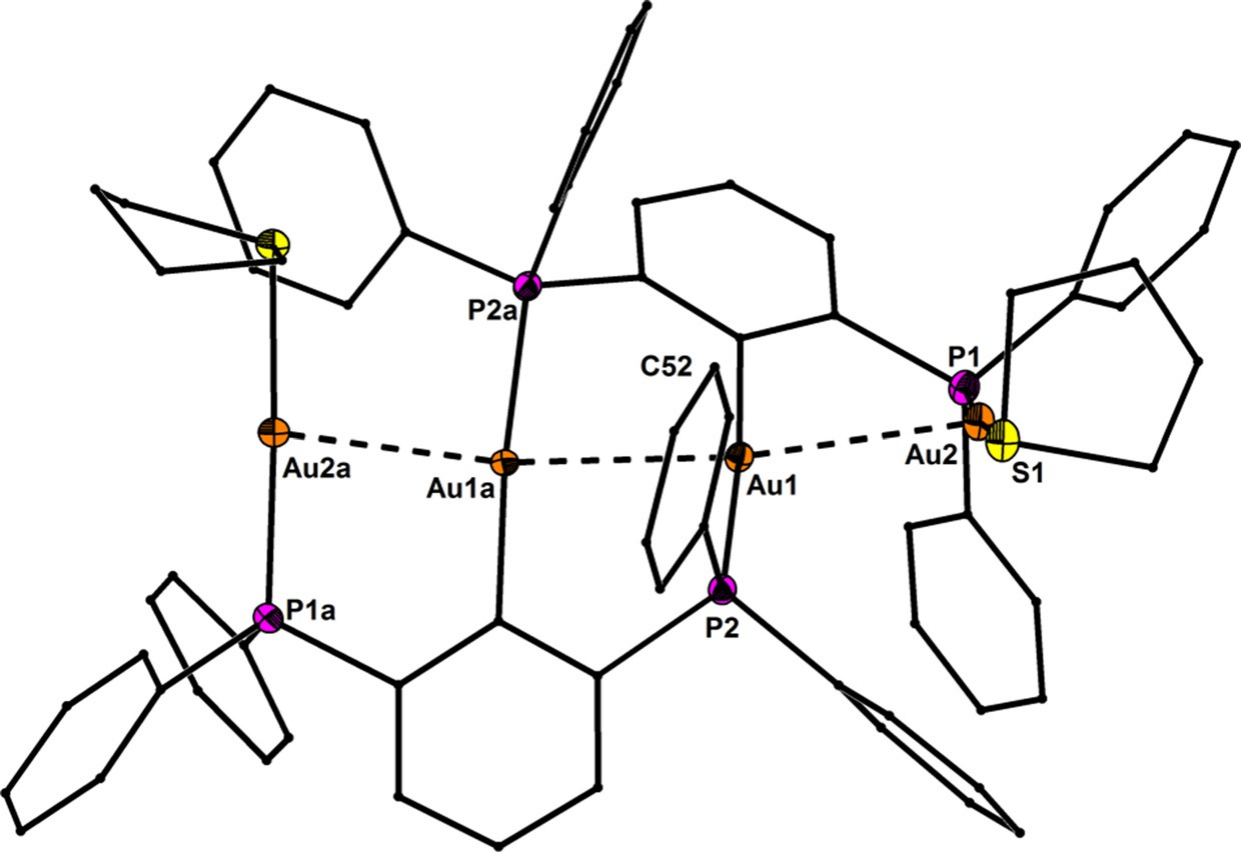
Molecular structure of **4** showing 50 % probability ellipsoids and the crystallographic numbering Scheme. The two BAr^F^_4_ counter anions are omitted for clarity. Selected bond lengths [Å]: Au1−C10 2.051(7), Au1−P2 2.305(2), Au2−P1 2.268(2), Au2−S1 2.338(2), Au1−Au1a 2.8280(5), Au1−Au2 2.9095(4).

**Figure 4:**
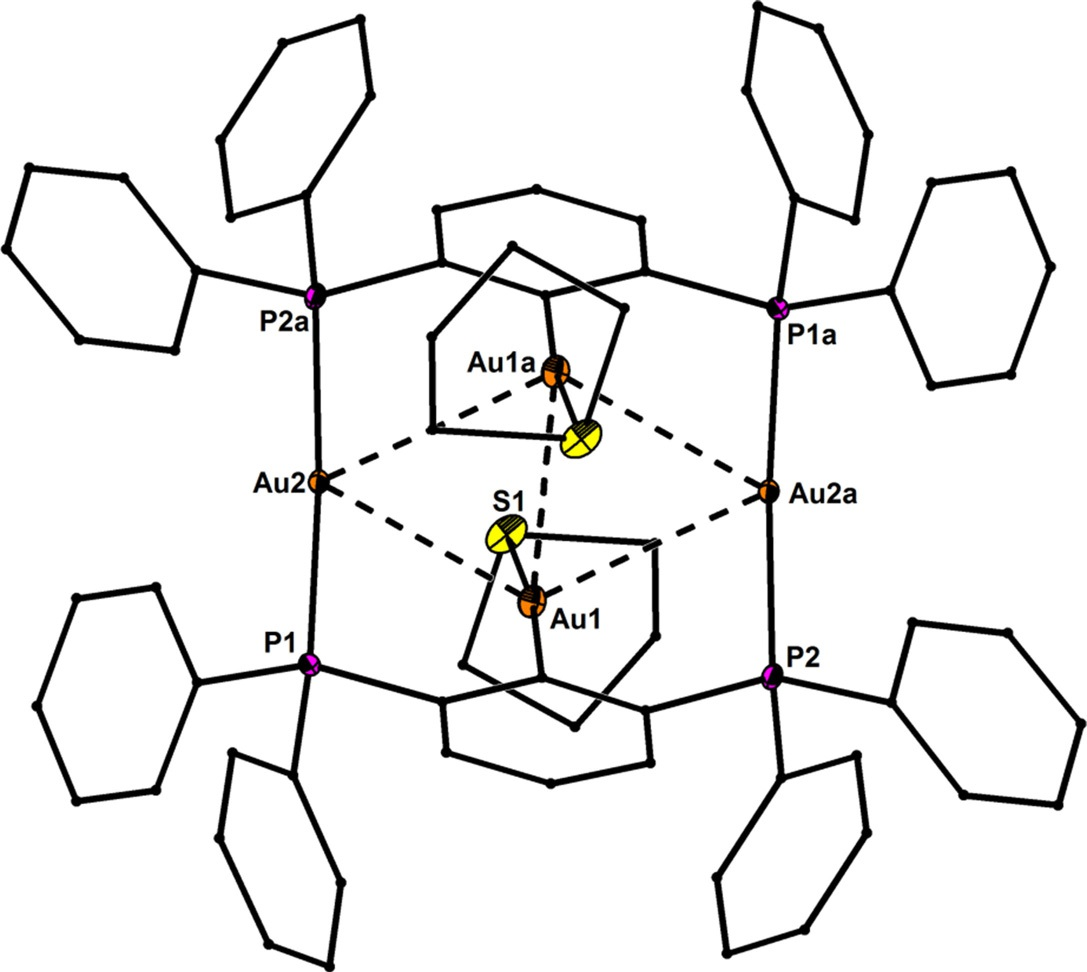
Molecular structure of **5** showing 50 % probability ellipsoids and the crystallographic numbering Scheme. The two BAr^F^_4_ counter anions are omitted for clarity. Selected bond lengths [Å]: Au1−C10 2.025(5), Au2−P1 2.319(1), Au2−P2a 2.313(1), Au1−S1 2.338(2), Au1−Au1a 3.1118(5), Au1−Au2 2.9980(3), Au1−Au2a 3.1356(3).

**Figure 5:**
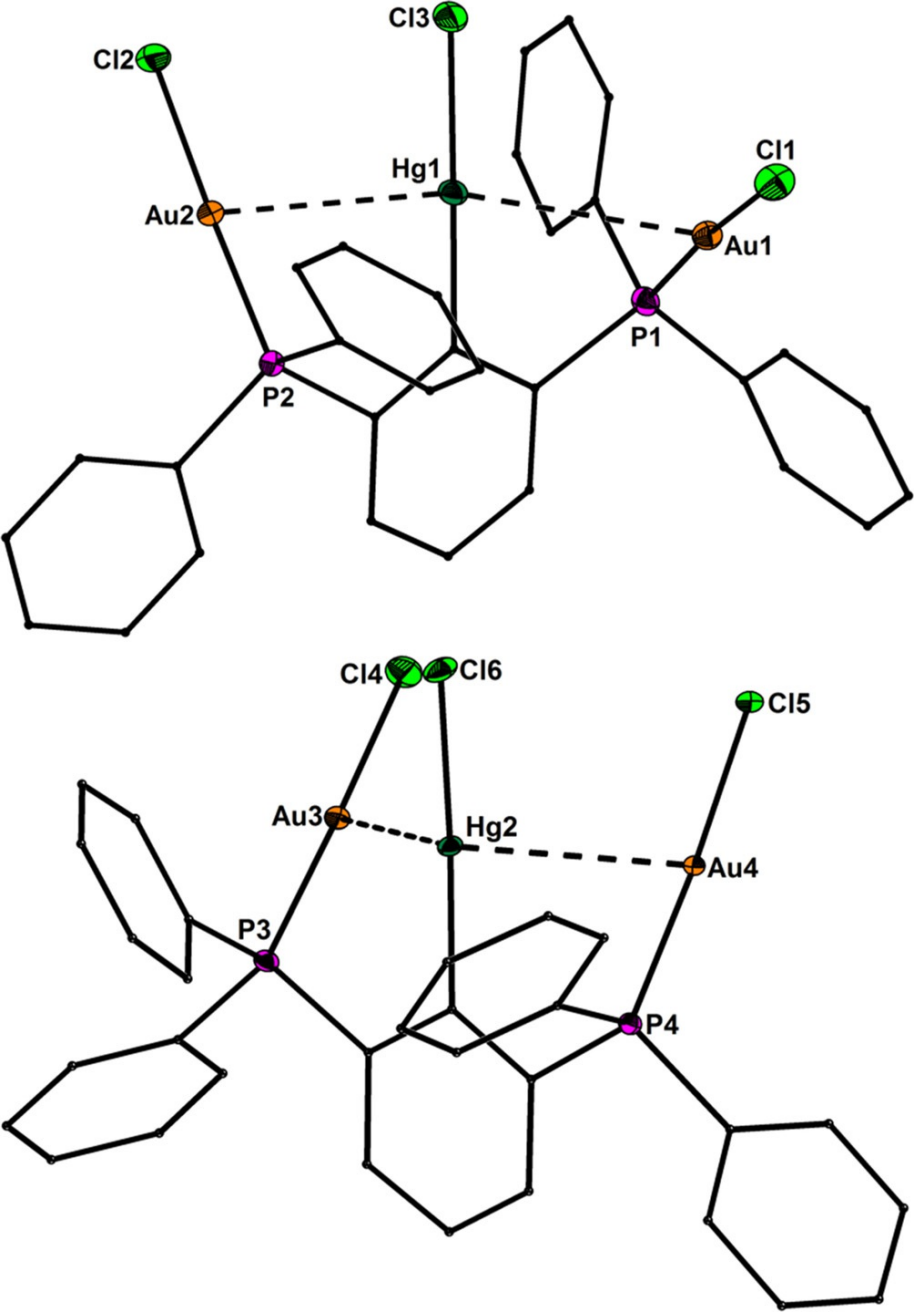
Molecular structures of the two independent conformers of **6** showing 50 % probability ellipsoids and the crystallographic numbering Scheme. Selected bond lengths [Å]: Hg1−C10 2.072(7), Hg2−C60 2.076(8), Hg1−Cl3 2.307(2), Hg2−Cl6 2.302(2), Au1−P1 2.235(2), Au2−P2 2.238(2), Au3−P3 2.239(2), Au4−P4 2.232(2), Au1−Cl1 2.283(2), Au2−Cl2 2.295(2), Au3−Cl4 2.284(2), Au4−Cl5 2.284(2), Au1−Hg1 3.2879(4), Au2−Hg1 3.0995(4), Au3−Hg2 3.4082(4), Au4−Hg2 3.0253(4).

Precise structural information was obtained from X‐ray crystallography. The molecular structures of **1**–**6** are shown in Figure 1–Figure 5. Selected bond lengths are collected in the caption of the figures. In all structures the Au^I^ and Hg^II^ atoms adopt almost linear spatial arrangements as anticipated for complexes with a 14 valence‐electron count. The related Au−C, Au−P, Au−S, Au−Cl, Hg−C, and Hg−Cl bond distances exhibit typical lengths.^5^, ^6^, ^9^ In the structure of **2** the Me_3_Si group is still present after the complexation of the two Au atoms (Figure 1). However, the displacement of the Si atom from the plane defined by the central phenyl ring increases notably upon going from **1** (0.072(1)) to **2** (0.219(1) Å), which might be a sign for the Si−C bond activation in **2**. However, the Si−C bond lengths of **1** (1.8721(1)) and **2** (1.878(6) Å) are indistinguishable within the experimental error. The molecular structures of **3** and **4** (Figure 2 and Figure 3) reveal a string arrangement of four Au atoms, which are associated by three Au–Au contacts (2.8280(5) to 3.0567(4) Å). The contact distances between the inner Au1 and Au3 atoms (2.8746(4) in **3**, 2.8280(5) Å) are significantly shorter than the intermetallic distances in similar cationic or neutral string gold complexes that display aurophilic interactions (e.g. for [Au_4_(dpmp)_2_(SCN)_2_]^2+^ the distance between the inner Au atoms is 3.0049(8) Å (dpmp=bis(diphenylphosphinomethyl)phenylphosphine).^15^ The neutral complex **3** entails two sets of two Au atoms in equivalent chemical environment: the organometallic coordination site encompasses two Au atoms each coordinated by a phenylate—and the Ph_2_P donor moiety in mutual *trans*‐position (Au1 and Au3). This pattern gives rise to a linear coordination sphere around Au atoms located in the center of the Au_4_ string. The second set of Au^I^ atoms terminate the Au_4_ gold string and reside likewise in a linear coordination motif. A P donor and a Cl ligand in mutual *trans*‐position build this linear coordination pattern (Au2 and Au4). The related dicationic complex **4** is centrosymmetric but retains the string‐like Au_4_ motif with its internal organometallic coordination site around Au1 and Au1a and the terminal inorganic coordination site. Due to the abstraction of both Cl ligands and the introduction of tht, the terminal Au moieties (Au2/Au2a) are coordinated by the P donor of the *PCP* ligand and the sulfur donor stemming from the thioether (tht), both in mutual *trans*‐arrangement. Two weakly coordinating [BAr^F^_4_]^−^ anions maintain the charge compensation for the dicationic complex. In contrast, the molecular structure of **5** (Figure 4) shows a planar rhomboidal motif with five significant Au⋅⋅⋅Au interactions (2.9980(3) to 3.1356(3) Å) in the cycle defined by Au1, Au2, Au1a, and Au2a, that are comparable to other similar complexes stabilized by tripodal phosphine ligands (dpmp) reported recently.^16^, ^17^ The increase in number of aurophilic contacts is due to the rhomboidal arrangement and the additional *trans*‐annular contact Au1⋅⋅⋅Au1a (3.1118(5) Å), which might be the thermodynamic driving force for the rearrangement of **4** into **5**. This rearrangement results into a redistribution of the ligands in organometallic and inorganic coordination sites. The gold atoms Au2 and Au2a show a homoleptic linear coordination of two Ph_2_P donors in mutual *trans*‐position, whereas the organometallic coordination site shows a mutual *trans*‐arrangement of a phenylate and a tht moiety to each gold atom of this site (Au1 and Au1a). The crystal structure of **6** comprises two crystallographically independent conformers, in which the two Au atoms chelate the central Hg atom (Figure 5). In this way, the two conformers adopt Au‐Hg‐Au *transoid* and *cisoid* arrangements, respectively. Overall, the Au⋅⋅⋅Hg contacts of **6** (3.0253(4) to 3.4082(4) Å) are somewhat longer than the Au–Au contacts in **3**–**5**, but are close to intermolecular Au⋅⋅⋅Hg distances (3.097(2)–3.498(3) Å) observed recently for a number of so called molecular Au–Hg amalgams,5f, 5g, 18 as well as the intramolecular Au⋅⋅⋅Hg distances (3.112(1)–3.2940(9) Å) observed for some similar complexes.5h, 14 Complex **6** may be also regarded as a metallo‐pincer complex [ClHg(*AuCAu*)] between Hg^II^ and the anionic tridentate ligand [2,6‐(Ph_2_PAuCl)_2_C_6_H_3_]^−^ (*AuCAu*)^−^ containing a central carbanionic binding site and two “gold‐arms” contributing pincer‐type chelation through metallophilic interactions. In a more general way, the (*PCP*)‐ligand **II** is extended by two metal units, which are coordinated to the PPh_2_ moieties in a linear fashion giving rise to an (*MCM*)‐metallo pincer ligand **V** comprised of a central carbanionic donor and two “metal‐arms” providing pincer‐type chelation through metallophilic interactions (Scheme 1).

### Photophysical properties

**Figure 6:**
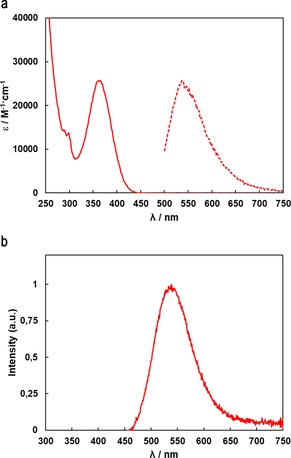
(a) Absorption (solid line) and emission (dashed line, a.u.) spectrum of **3** in CH_2_Cl_2_ solution. (b) Emission spectrum of **3** in the solid state (excitation at 365 nm).

Only in case of **3**, bright luminescence can be observed upon exposure to UV‐A light. The UV/Vis absorption spectrum and the emission spectrum of **3** in CH_2_Cl_2_ solution are shown in Figure 6 a. An absorption maximum at 365 nm and another strong absorption band reaching into the UV region below 250 nm are observed. The emission spectra in CH_2_Cl_2_ solution and in the solid state reveal maxima at 538 and 539 nm, respectively, which is associated with the emission of yellow‐green light. Photoluminescence quantum yields of 4.3 % in dichloromethane solution and 17 % in the solid state were found. Similar emissive properties although at higher quantum yields in solution or solid state, have been reported for linear polynuclear string gold complexes.^17^ The photoluminescence spectrum of **3** in the solid state is shown in Figure 6 b.

### DFT analysis

**Figure 7:**
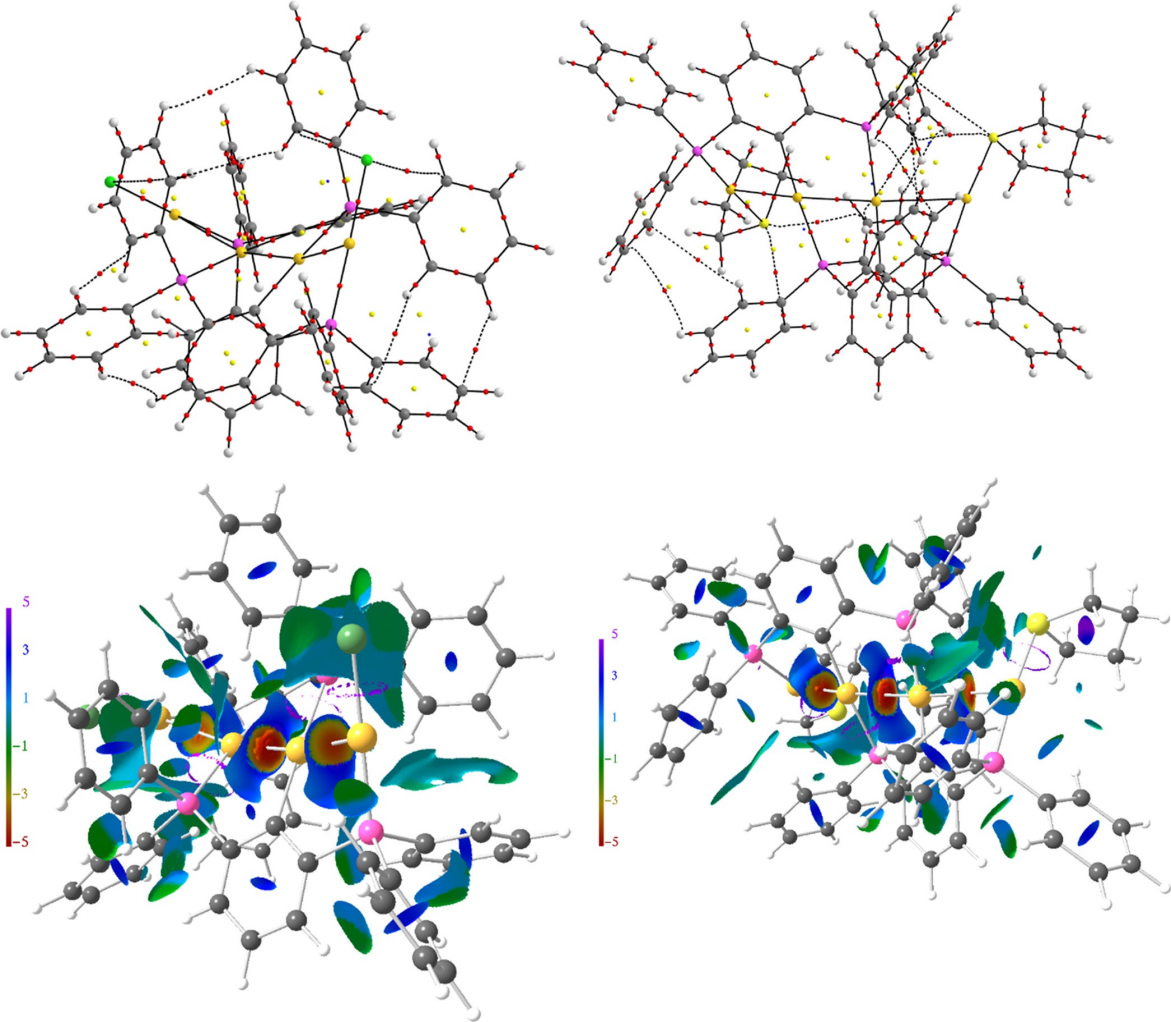
AIM bond paths′ motifs and NCI isosurfaces (*s*=0.5) of compounds **3** (left) and **4** (right). Atom colors are as follows: Au=gold, Cl=green, P=pink, C=grey, H=white.

**Figure 8:**
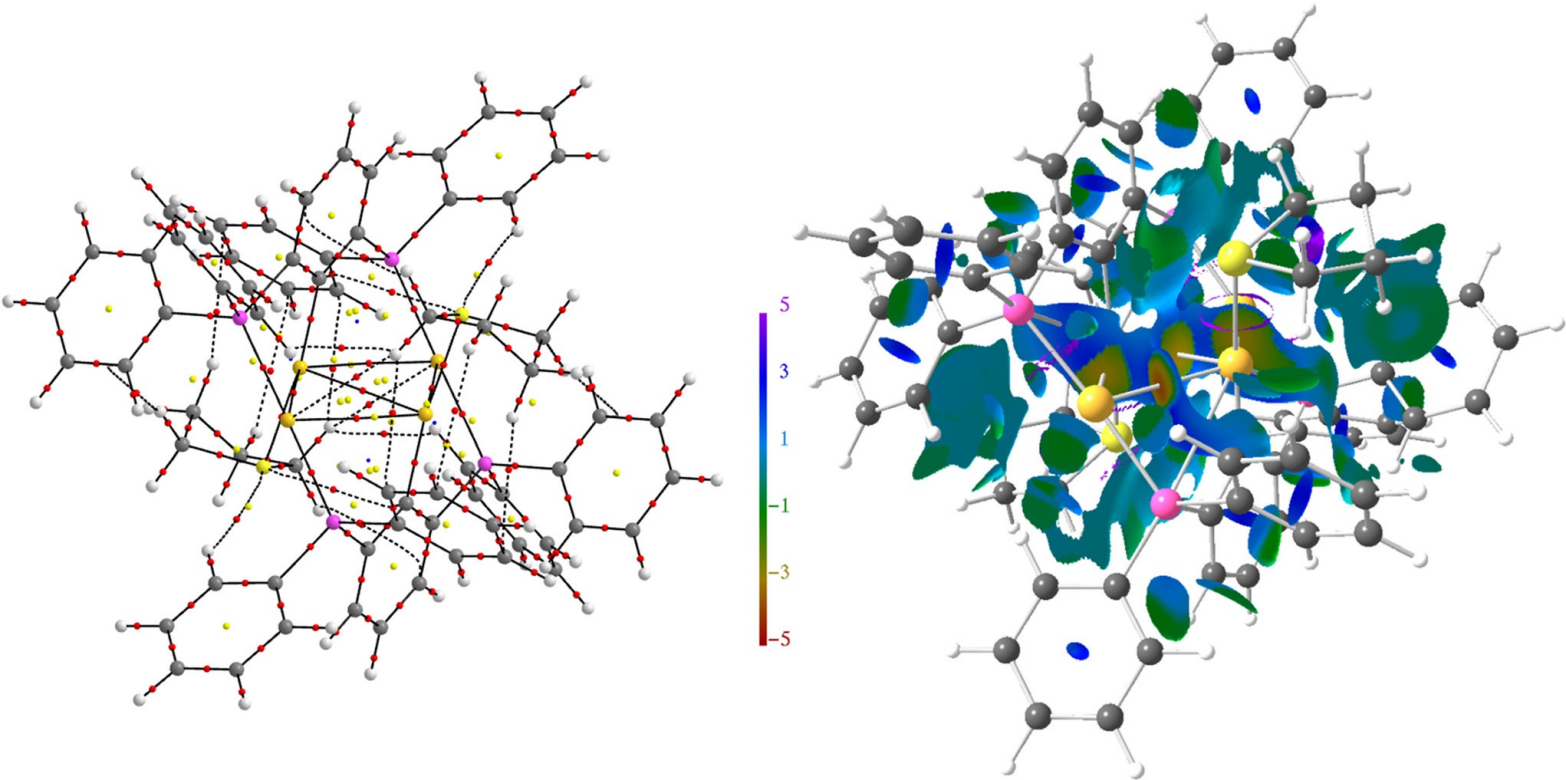
AIM bond paths′ motifs and NCI isosurfaces (*s*=0.5) of compound **5**. Atom colors are as follows: Au=gold, Cl=green, P=pink, C=grey, H=white.

**Figure 9:**
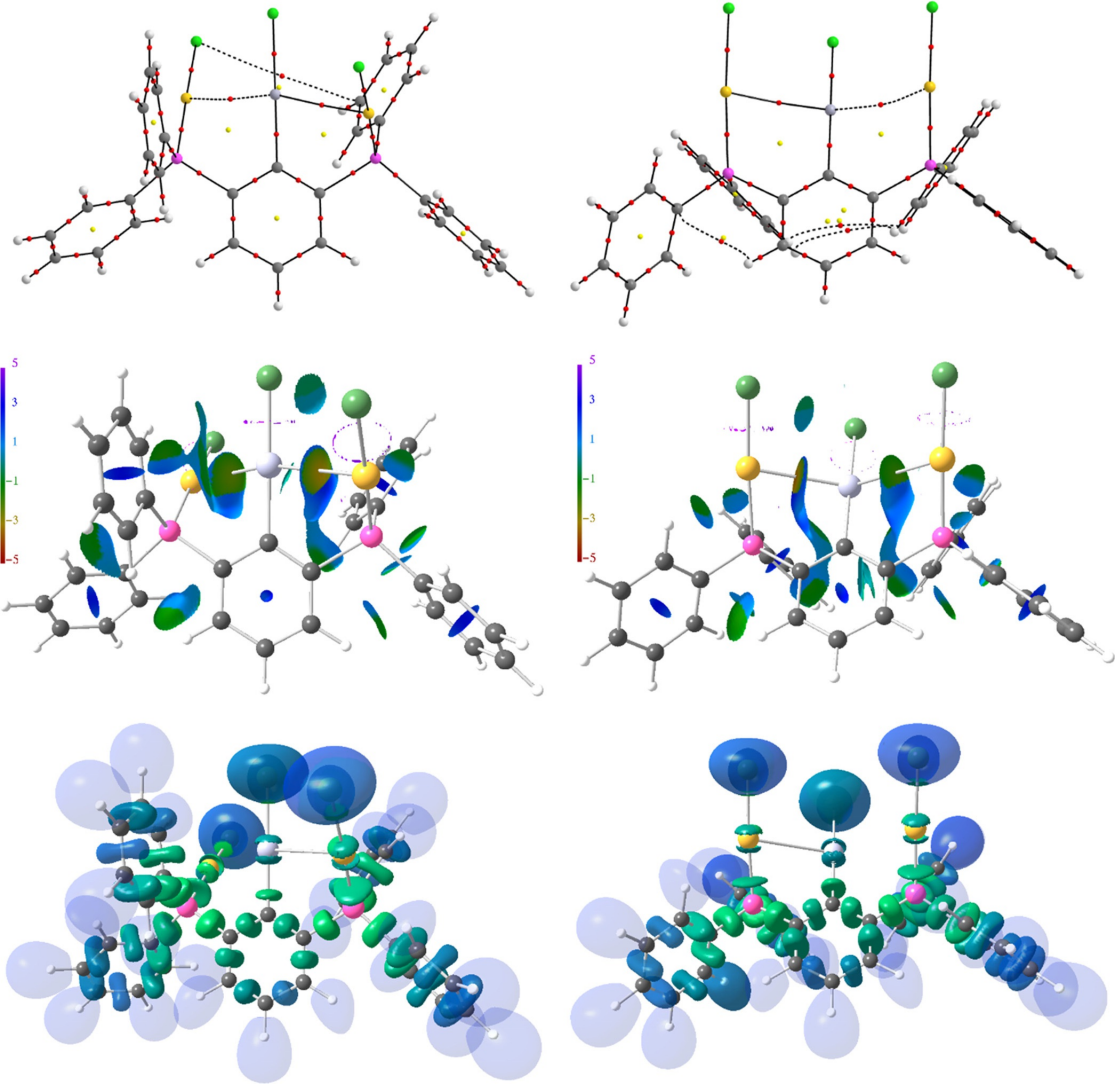
AIM bond paths′ motifs, NCI isosurfaces (*s*=0.5), and ELI‐D isosurfaces (Y=1.3) of the *cis*‐ and *trans*‐conformers of **6**. Atom colors are as follows: Au=gold, Hg=light gray, Cl=green, P=pink, C=grey, H=white.

Complementing the interpretation of the structural parameters, electronic bond characteristics of the metallophilic Au⋅⋅⋅Au and Au⋅⋅⋅Hg interactions were examined in terms of computed real‐space bonding indicators (RSBI). All calculations are based on the experimentally obtained XRD structures with C−H distances corrected in order to obey neutron diffraction results.^19^ The RSBI set comprises parameters extracted from topological analysis of the electron and pair densities according to the Atoms In Molecules (AIM)^20^ and Electron Localizability Indicator (ELI‐D)^21^ space‐partitioning schemes as well as a surface study within the framework of the recently introduced Non‐Covalent Interactions (NCI) index,^22^ which is based on the reduced density gradient (*s*) of the electron density (ED) and unravels noncovalent interaction areas. Thus, the NCI transcend topological approaches, which mainly rely on stationary point analysis as it also detects weak intra‐ and intermolecular interactions, such as London dispersion,^23^ for which not essentially bond‐critical points (bcp) are detectable in the underlying ED. By mapping the second Eigenvalue (*λ*_2_) of the ED‐Laplacian (∇^2^ *ρ*=*λ*_1_+*λ*_2_+*λ*_3_) on *s*, bonding (*λ*_2_<0) can be distinguished from weak Van der Waals (VdW) forces (*λ*_2_≈0), or steric repulsion (*λ*_2_>0). The topological AIM bond paths′ motifs, which are typically referred to resemble the molecular structures, are displayed in Figures 7–Figure 9 for compounds **3**, **4**, **5**, as well as the *transoid* and *cisoid* conformers of **6**. For all cases, metallophilic Au⋅⋅⋅Au or Au⋅⋅⋅Hg attraction is disclosed by formation of corresponding bcp in the ED, the topological parameters of which are typical for this kind of interactions (Table 1).^6^, ^24^ The low value of the ED at the bcp (*ρ*_bcp_ approx. 0.1–0.3 e Å^−3^), the positive but close to zero value of the Laplacian (∇^2^ *ρ*_bcp_ approx. 1–3 e Å^−5^), as well as the dominance of the kinetic energy density over ED ratio (*G*/*ρ*_bcp_ approx. 0.6–0.8 *h* e^−1^) against the total energy density over ED ratio (*H*/*ρ*_bcp_ approx. −0.2–0.0 *h* e^−1^) uncovers these contacts to be mainly noncovalent (Table 1). This is supported by the integrated delocalization index, *δ*(A,B), which quantifies the number of electron pairs shared between two adjacent or distant atoms and lies in the range of 0.2 to 0.4. Similar values are typically observed for ionic atom‐atom contacts with the only difference that ∇^2^ *ρ*_bcp_ and *G*/*ρ*_bcp_ values are larger positive for the latter. Consequently, the NCI surface analysis of **3**–**6** shows pronounced features along the Au⋅⋅⋅Au and Au⋅⋅⋅Hg interaction axes in terms of disc‐shaped reduced density gradient basins with highly negative *λ*_2_ values on the surface suggesting attractive metallophilic interactions (Figures 7), whereas no corresponding ELI‐D basins are formed (Figure 8, Figure 9).^6^, ^24^ In the NCI, weaker attractive or even repelling H⋅⋅⋅H and H⋅⋅⋅π interactions are also observed, which determine the spatial orientation of the different molecular fragments (e.g. phenyl groups) and thus the three‐dimensional appearance of the molecule in the crystal. With a considerably higher ED at the bcp of about 0.7–0.9 e Å^−3^ but Laplacian values (approx. 1–5 e Å^−5^) similar to the Au⋅⋅⋅Au and Au⋅⋅⋅Hg bonds the Au/Hg−Cl/P/C bonds combine covalent as well as noncovalent bonding aspects and may thus be regarded as polarized covalent.

**Table 1:** Topological bond descriptors and delocalization index of prominent bonds of **3**–**6**.

	Bond	*d* [Å]	*d*_1_/*d*	*ρ*_bcp_ [e Å^−3^]	∇^2^ *ρ*_bcp_ [e Å^−5^]	*ϵ*	*G*/*ρ*_bcp_ [a.u.]	*H*/*ρ*_bcp_ [a.u.]	*δ*(A,B)
**3**	Au1−Au2	2.983	0.50	0.23	1.9	0.05	0.70	−0.12	0.36
	Au3−Au4	2.945	0.50	0.25	2.1	0.06	0.72	−0.13	0.38
	Au1−Au3	2.843	0.50	0.30	2.5	0.03	0.76	−0.17	0.43
	Au2−Cl1	2.292	0.50	0.70	5.3	0.00	0.90	−0.37	1.01
	Au2−P1	2.238	0.52	0.85	1.7	0.01	0.64	−0.50	1.02
	Au1−*C_ph_*	2.068	0.54	0.90	4.0	0.04	0.78	−0.46	0.91
**4**	Au1−Au2	2.910	0.50	0.27	2.2	0.04	0.73	−0.15	0.41
	Au1−Au1a	2.827	0.50	0.31	2.6	0.04	0.76	−0.18	0.44
	Au1a−Au2a	2.910	0.50	0.27	2.2	0.04	0.73	−0.15	0.41
	Au1−P2	2.305	0.51	0.76	2.0	0.01	0.64	−0.45	0.89
	Au2−S1	2.338	0.50	0.69	4.2	0.03	0.80	−0.38	0.87
	Au1−*C_ph_*	2.052	0.54	0.93	4.0	0.03	0.77	−0.48	0.92
**5**	Au1a−Au2a	2.998	0.50	0.22	1.9	0.10	0.72	−0.11	0.31
	Au1−Au1a	3.111	0.50	0.18	1.6	0.10	0.69	−0.07	0.25
	Au1a−Au2	3.135	0.50	0.17	1.5	0.16	0.67	−0.07	0.24
	Au1−Au2a	3.135	0.50	0.17	1.5	0.16	0.67	−0.07	0.24
	Au1−Au2	2.998	0.50	0.22	1.9	0.10	0.72	−0.11	0.31
	Au2a−P1a	2.319	0.51	0.75	1.7	0.01	0.60	−0.45	0.88
	Au1−S1	2.339	0.50	0.69	4.3	0.02	0.82	−0.38	0.84
	Au1−*C_ph_*	2.024	0.55	0.99	3.7	0.04	0.76	−0.50	0.95
	Au2−H_tht_	2.952	0.63	0.06	0.5	0.17	0.59	0.08	0.04
*trans*‐**6**	Au1−Hg1	3.288	0.51	0.13	1.1	0.33	0.61	−0.01	0.18
	Au2−Hg1	3.099	0.51	0.18	1.6	0.14	0.67	−0.05	0.25
	Hg1−*C_ph_*	2.073	0.55	0.90	3.0	0.04	0.70	−0.46	0.88
	Au1−Cl1	2.282	0.50	0.72	5.3	0.00	0.90	−0.38	1.06
	Au1−P1	2.236	0.52	0.85	1.6	0.01	0.64	−0.50	1.03
*cis*‐**6**	Au3−Hg2	3.408	0.52	0.11	0.9	0.87	0.58	0.03	0.15
	Au4−Hg2	3.026	0.51	0.20	1.8	0.10	0.70	−0.08	0.28
	Hg1−*C_ph_*	2.075	0.55	0.90	3.0	0.04	0.70	−0.46	0.88
	Au3−Cl4	2.285	0.50	0.72	5.2	0.00	0.89	−0.38	1.07
	Au3−P3	2.239	0.52	0.84	1.8	0.00	0.64	−0.50	1.03

*ρ*_bcp_: electron density, ∇^2^ *ρ*_bcp_: Laplacian, *d*_1_/*d*: ratio, *ϵ*: bond ellipticity, *G*/*ρ*_bcp_ and H/*ρ*_bcp_: kinetic and total energy density over *ρ*_bcp_ ratios, *δ*: delocalization index.

Accordingly, both *G*/*ρ*_bcp_ (approx. 0.6–0.9 *h* e^−1^) and *H*/*ρ*_bcp_ (approx. −0.5–0.4 *h* e^−1^), show strongly positive and negative values, respectively. The covalent character is further supported by *δ*(A,B) being close to or even above 1 and the formation of Au−Cl/P and Hg−Cl/C bonding basins in the ELI‐D (Figure 8). Due to the higher Pauling electronegativity of Au atoms (2.4) compared with Hg atoms (1.9) the AIM atomic charges are close to zero for the former (Q^AIM^_(Au)_=−0.09–0.07 e), but positive for the latter (Q^AIM^_(Hg)_=0.64–0.65 e), which confirms previous results.^6^, ^24^ As anticipated, Cl atomic charges are negative (approx. −0.5 e), whereas P atomic charges are highly positive (approx. 1.8 e) within AIM space‐partitioning (see the Supporting Information, Tables S3–S6 for a full list).

## Summary and Conclusions

The synthesis and characterization of the tetranuclear gold complexes *linear*‐[Au_4_Cl_2_(*PCP*)_2_] (3), *linear*‐[Au_4_(*PCP*)_2_(tht)_2_][BAr^F^_4_]_2_ (**4**), *cyclo*‐[Au_4_(*PCP*)_2_(tht)_2_][BAr^F^_4_]_2_ (**5**), and the trinuclear bimetallic complex [HgAu_2_Cl_3_(*PCP*)] (**6**) were reported, whereby (*PCP*)^−^ comprises the novel tridentate carbanionic ligand [2,6‐(Ph_2_P)_2_C_6_H_3_]^−^ (**II**). Compounds **4** and **5** are metal‐string complexes in which four Au atoms are associated by three aurophilic interactions in very similar linear chain arrangements. Compound **3** shows yellow‐green photoluminescence both in dichloromethane solution and in the solid state (*λ*_max_=538 and 539 nm, respectively). UV‐light triggers an irreversible rearrangement from **4** into the isomer **5** in which four Au atoms are associated by five aurophilic interactions in a rhomboidal arrangement. Compound **6** can be regarded as metallo‐pincer complex [ClHg(*AuCAu*)], whereby (*AuCAu*)^−^ comprises the novel tridentate carbanionic ligand [2,6[2,6‐(Ph_2_PAuCl)_2_C_6_H_3_]^−^ (**V**) containing a central carbanionic binding site and two “gold‐arms” contributing pincer‐type chelation through two metallophilic Au−Hg interactions (Scheme 1). All metallophilic interactions of **3**–**6** give rise to AIM bond paths and bond critical points. Typically for noncovalent interactions, the NCI shows contact patches where the Au⋅⋅⋅Au and Au⋅⋅⋅Hg interactions occur, while the ELI‐D remains featureless demonstrating the complementarity of these real‐space bond indicators.^25^

## Conflict of interest

The authors declare no conflict of interest.

## Supporting information

As a service to our authors and readers, this journal provides supporting information supplied by the authors. Such materials are peer reviewed and may be re‐organized for online delivery, but are not copy‐edited or typeset. Technical support issues arising from supporting information (other than missing files) should be addressed to the authors.

Supplementary
