# Supplementary material for: Tri‐ and Tetranuclear Metal‐String Complexes with Metallophilic d10–d10 Interactions
Source: Chemistry. 2019 Nov 26;26(1):275–84. doi: 10.1002/chem.201904106 (PMC6972983; doi:10.1002/chem.201904106)
Supplement: Supplementary file 1 — Supplementary [file CHEM-26-275-s001.pdf]

CHEMISTRY
A European Journal

Supporting Information

Tri- and Tetranuclear Metal-String Complexes with Metallophilic d^{10}–d^{10} Interactions

Marian Olaru,[a] Julius F. Kögel,[a] Risa Aoki,[b] Ryota Sakamoto,[b, c] Hiroshi Nishihara,[b] Enno Lork,[a] Stefan Mebs,*[d] Matthias Vogt,*[a] and Jens Beckmann*[a]

chem_201904106_sm_miscellaneous_information.pdf

Contents

Experimental section ... 2

General information. ... 2

Synthesis and characterisation of **1** ... 4

Synthesis and characterisation of **2** ... 7

Synthesis and characterisation of **3** ... 10

Synthesis and characterisation of **4** ... 13

Isolation of single crystals of **4** suitable for an X-ray diffraction study ... 17

Synthesis and characterisation of **5** ... 18

Synthesis and characterisation of **6** ... 22

HRMS spectra ... 25

X-ray crystallography ... 37

Computational methodology ... 40

References ... 50

Experimental section

General information.

Reagents were obtained commercially and were used as received except otherwise noted. Anhydrous solvents were collected from a SPS800 mBraun solvent purification system. Caesium fluoride was dried at 150°C (1×10^{-2} mbar) for 16h and stored in the glovebox. Starting material 2,6-$F_2C_6H_3SiMe_3$[20] was prepared following the protocol describing the synthesis of the related derivative 2,6-$F_2C_6H_3SiEt_3$.[21] Na[B{3,5-$(CF_3)_2C_6H_3$}$_4$] was prepared according to literature procedures.[22] NMR spectra (^{1}H, ^{13}C, ^{31}P, ^{19}F, ^{199}Hg, COSY, HSQC and HMBC) were recorded at room temperature on Bruker Avance III 400 and Bruker Avance III 600 spectrometers. Some ^{31}P NMR spectra were measured on Bruker Avance DPX 200. ^{29}Si-DEPT NMR spectra were measured at room temperature using a Bruker Avance 360 spectrometer. ^{1}H, ^{13}C chemical shifts are reported in δ units (ppm) relative to the residual peak of solvent ($CHCl_3$, 7.26 ppm; $(CD_3)S(O)CD_2H$ 2.50 ppm, $CDHCl_2$ 5.32 ppm) in the ^{1}H NMR spectra, and to the peak of the deuterated solvent ($CDCl_3$ 77.16 ppm; [D$_6$]DMSO 39.52 ppm, , CD_2Cl_2 53.84 ppm) in ^{13}C NMR spectra.[23] ^{1}H, ^{13}C, ^{29}Si ^{19}F, NMR, ^{31}P and ^{199}Hg spectra are reported in δ units (ppm) and are referenced against Me_4Si, $CFCl_3$, H_3PO_4 (85% in water) and $HgCl_2$ (in [D$_6$]DMSO) respectively, using the chemical shift of the lock solvent as a reference. Because the signals corresponding to the aromatic protons are overlapping, the HMBC spectra were not helpful for the assignment of all ^{13}C resonance signals. Assignment of the coupling constants and the ^{13}C resonance signals were made only in the clear-cut cases. The labelling scheme is shown in the supporting information. UV/vis spectra were recorded on a JASCO V-570 spectrometer. Photoluminescence spectra were collected using a HITACHI F-4500 spectrometer. Absolute photoluminescence quantum yields were measured by a Hamamatsu Photonics C9920-02G. The solutions were prepared using spectroscopic grade dichloromethane (Wako). Nitrogen was bubbled through the solutions for three minutes before photoluminescence spectra and quantum yields were measured. HRMS APCI(+) and ESI(+) spectra were recorded on a Thermo Scientific Orbitrap XL spectrometer. Data analysis and calculation of the theoretical isotopic patterns were carried out with Xcalibur® software package.[24]

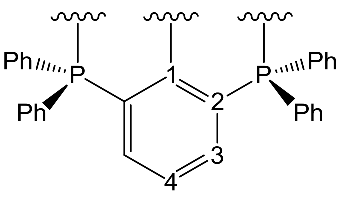

1, 2, 5, 6

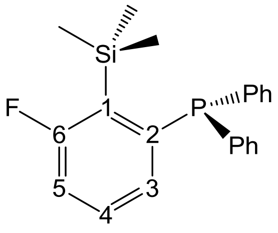

1a

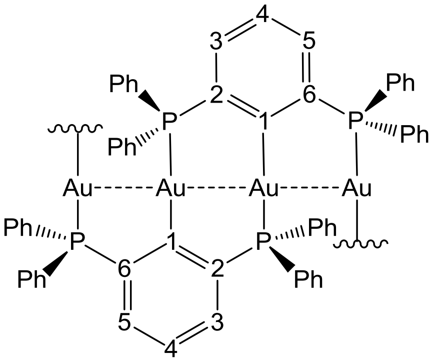

3, 4

Scheme S1. Labeling scheme used for assignment of ^{1}H and ^{13}C NMR resonance signals of **1**–**6**. The labels of the hydrogen atoms follow the carbon numbering scheme.

Synthesis and characterisation of 1

A 250 mL three neck round bottom flask fitted with a reflux condenser and argon inlet was charged, under argon, with lithium shots (2.15 g, 307 mmol) and anhydrous THF (120 mL). The contents of the flask were cooled to 0°C, then Ph_2PCl (33.9 g, 153 mmol) was added slowly via a syringe and the mixture stirred 0 to 14 °C for 10 h; after 50 minutes the colour changed to blood-red. To the Ph_2PLi solution was added 2,6-$F_2C_6H_3SiMe_3$ (9.0 g, 48 mmol). The reaction was refluxed at 90–95°C (oil bath temperature) for the next 48 h. All volatiles were removed at reduced pressure and to the semi-solid brown residue was added MeCN (150 mL). The resulting suspension was filtered (in air) on a glass frit. The solid was collected from the frit and suspended in $CHCl_3$ (approx. 150 mL). The grey suspension was filtered through a pad of Celite to remove lithium salts. After all volatiles were removed by rotary evaporation, the resulting grey solid (16.3 g) was purified by flash chromatography with $CHCl_3$ using silicagel as stationary phase to obtain **1** (14.0 g, 54 %) as a white solid. Compound **1** is poorly soluble in MeCN and hexanes (at rt), and soluble in halogenated solvents (CH_2Cl_2, $CHCl_3$), aromatic solvents (toluene, benzene), THF and Et_2O. **Mp** 128–130 °C. **^{1}H NMR (400 MHz, $CDCl_3$, 300 K)**: δ = 7.36 – 7.28 (m, 12H, P*Ph*$_2$), 7.29 – 7.19 (m, 8H, P*Ph*$_2$), 7.12 – 7.09 (m, 3H, H3 and H4), 0.57 ppm (t, $^5J(^1H–^{31}P)$ = 2.6 Hz, 9H, Si*Me*$_3$). **^{13}C NMR (100 MHz, $CDCl_3$, 300 K)**: δ = 155.47 (t, $^2J(^{13}C–^{31}P)$ = 40.5 Hz, C1), 145.23 (dd, $^1J(^{13}C–^{31}P)$ = 18.5, $^3J(^{13}C–^{31}P)$ = 12.9 Hz, C2), 138.98 – 138.67 (m, *i*-C of P*Ph*$_2$), 135.94 (s, C3 or C4), 133.60 (d, *J* = 19.8 Hz, CH of P*Ph*$_2$), 128.70 – 128.34 (multiple signals overlapped: 2×CH of P*Ph*$_2$, and C3 or C4), 6.52 ppm (t, $^4J(^{13}C–^{31}P$ = 13.5 Hz., $SiMe_3$). **^{31}P NMR (81 MHz, $CDCl_3$, 300 K)**: δ = −5.1 ppm (s). **^{29}Si NMR (72 MHz, $CDCl_3$, 300 K)**: δ = −2.99 (t, $^3J(^{29}Si–^{31}P)$ = 7.8 Hz). HRMS-APCI (*m*/*z*): $[M+H]^+$ calcd for $C_{33}H_{33}P_2Si$, 519.18213; found, 519.18065.

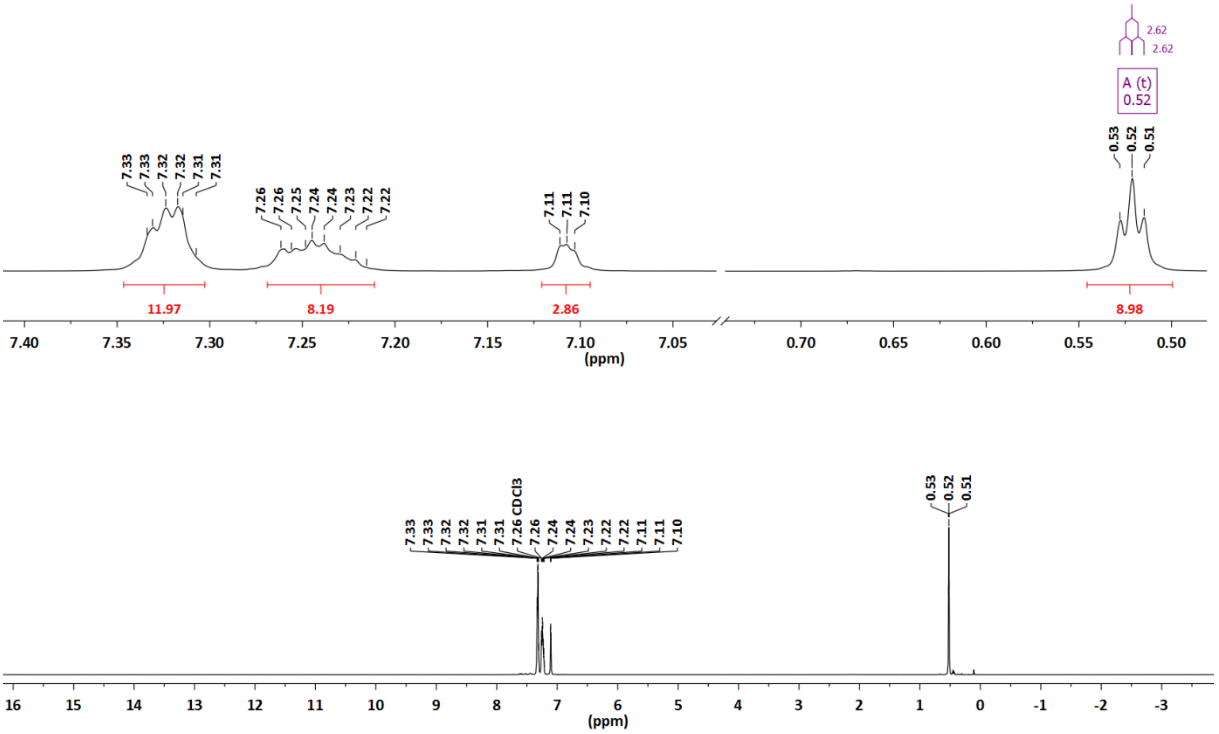

Figure S2: ^{1}H NMR ($CDCl_3$, 400 MHz) spectrum of **1**.

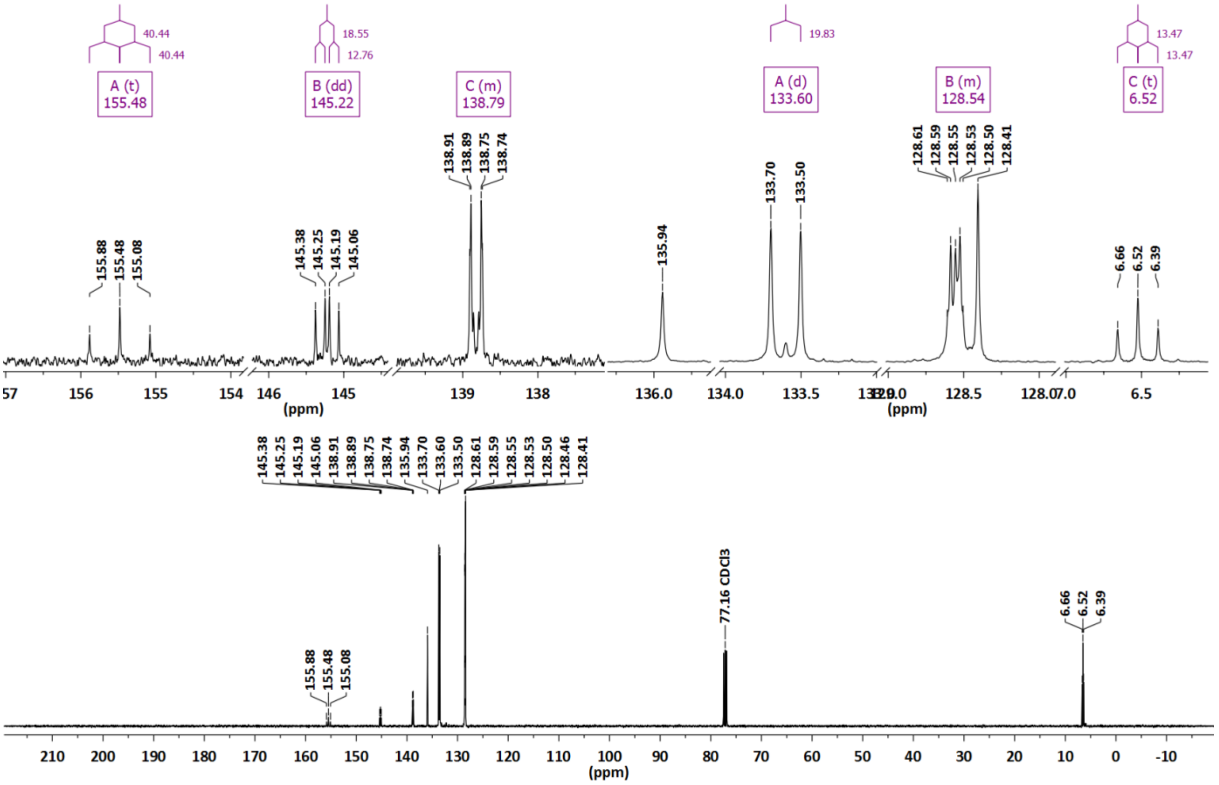

Figure S3: ^{13}C NMR ($CDCl_3$, 101 MHz) spectrum of **1**.

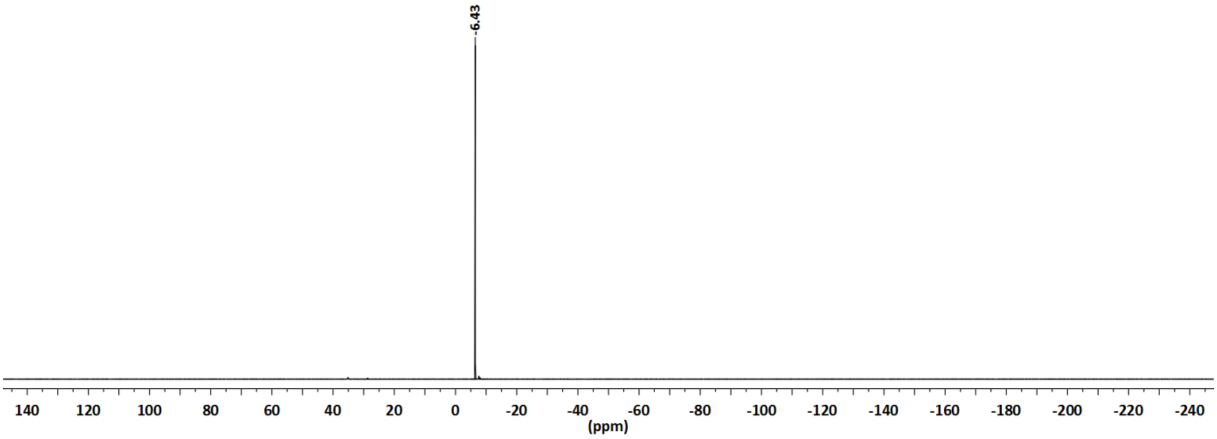

Figure S4: ^{31}P NMR ($CDCl_3$, 162 MHz) spectrum of **1**.

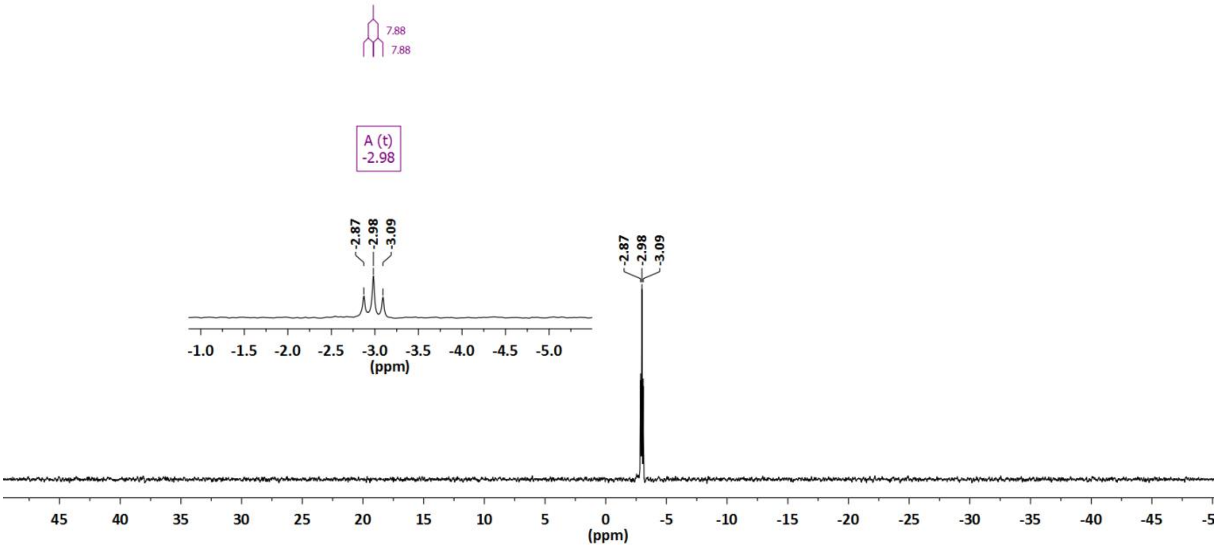

Figure S5: ^{29}Si NMR ($CDCl_3$, 72 MHz) spectrum of **1**.

Synthesis and characterisation of 2

To a mixture of **1** (53 mg, 0.10 mmol) and [AuCl(tht)] (65 mg, 0.2 mmol) CH_2Cl_2 (2 mL) was added and the mixture stirred approx. 5 minutes. The resulting colourless solution was layered with *n*-hexane (3 mL). The layered solution was allowed to stand for 12 h. The formed crystals were collected and washed with 1×1 mL $CHCl_3$ and 3×2 mL *n*-hexane and subsequently dried to obtain **2** as colourless crystals (90 mg, 90%). **Mp**. 175–180 °C. Compound **2** is poorly soluble in most organic solvents. Compound **2** is stable to air and moisture. **^{1}H NMR (600 MHz, [D$_6$]DMSO, 300 K)**: δ = 7.66 – 7.57 (m, 12H, P*Ph*$_2$), 7.55 – 7.48 (m, 8H, P*Ph*$_2$), 7.46 (t, $^3J(^1H–^1H)$ = 7.9 Hz, 1H, H4,), 7.35 – 7.30 (m, 2H, H3), 0.58 ppm (s, 9H, Si*Me*$_3$). **^{13}C NMR (151 MHz, [D$_6$]DMSO, 300 K)**: δ = 155.58 (t, $^2J(^{13}C–^{31}P)$ = 25.3 Hz, C1), 139.39 – 139.19 (m, br, C3), 137.52 (dd, $^1J(^{13}C–^{31}P)$ = 57.6, $^3J(^{13}C–^{31}P)$ = 15.3 Hz, C2), 133.67 (d, *J* = 14.1 Hz, CH of P*Ph*$_2$), 132.13 (s, *p*-CH of P*Ph*$_2$), 130.44 (d, *J* = 61.2 Hz, *i*-C of P*Ph*$_2$), 129.80 (d, $^1J(^{13}C–^{31}P)$ = 12.4 Hz, CH of P*Ph*$_2$), 128.77 (t, $^3J(^{13}C–^{31}P)$ = 9.7 Hz, C4), 8.22 ppm (t, $^4J(^{13}C–^{31}P)$ = 2.5 Hz, Si*Me*$_3$). **^{31}P NMR (243 MHz, [D$_6$]DMSO, 300 K)**: δ = 33.84 ppm (s). **^{29}Si-DEPT NMR (72 MHz, [D$_6$]DMSO, 300 K)**: δ = resonance was not observed due to poor solubility of the compound. HRMS-ESI (*m*/*z*): [M−Cl]$^+$ calcd for $C_{33}H_{32}Au_2ClP_2Si$, 947.07626; found, 947.07837.

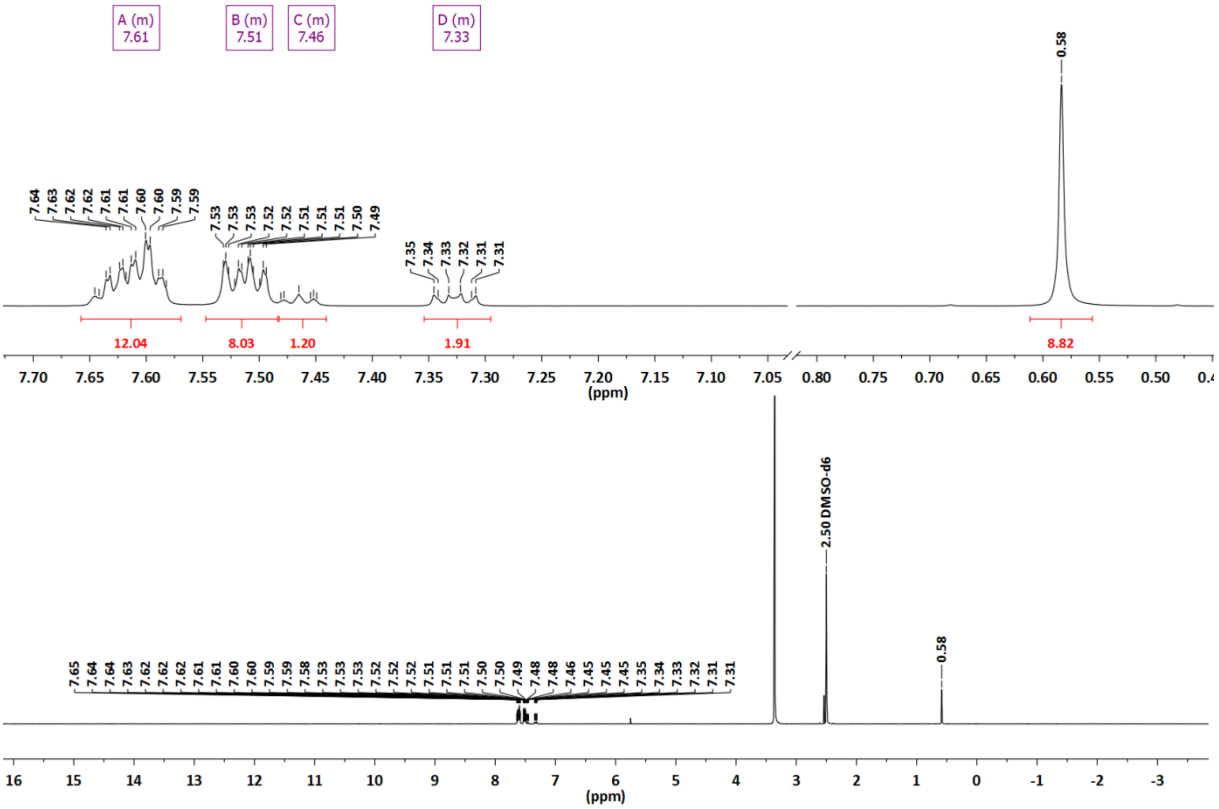

Figure S6: ^{1}H NMR ([D$_6$]DMSO, 600 MHz) spectrum of **2**.

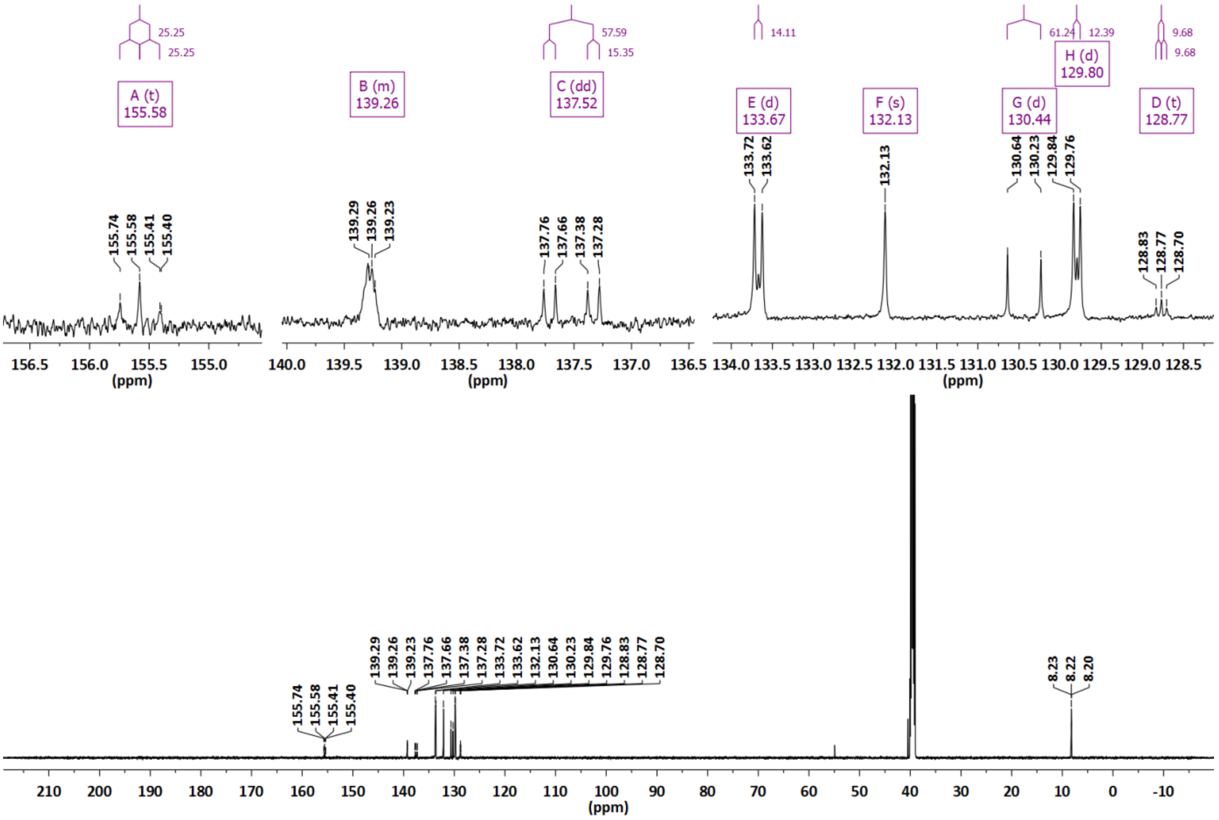

Figure S7: ^{13}C NMR ([D$_6$]DMSO, 151 MHz) spectrum of **2**.

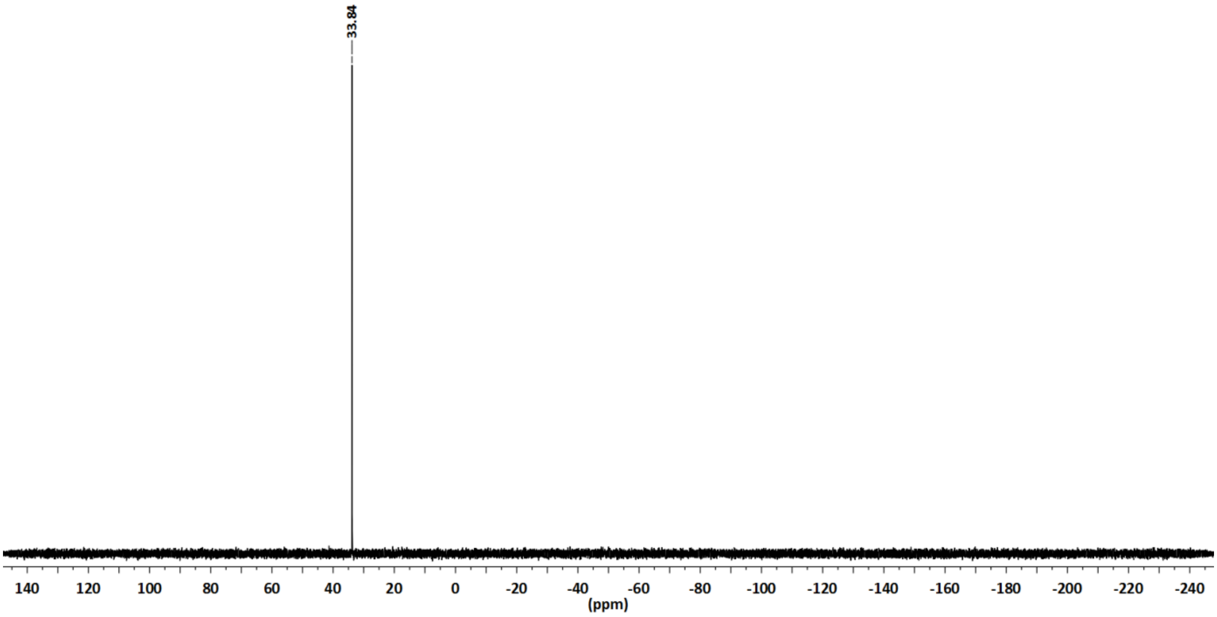

Figure S8: ^{31}P NMR ([D$_6$]DMSO, 243 MHz) spectrum of **2**.

Synthesis and characterisation of 3

Under inert conditions, a 25 mL Schlenk tube fitted with a Teflon valve was charged with **1** (104 mg, 0.2 mmol), [AuCl(tht)] (129 mg, 0.4 mmol, 2 equiv.) and anhydrous CsF (73 mg, 0.48 mmol, 2.4 equiv.). The mixture was suspended in THF/MeCN (1:1 ratio, 10 mL). The suspension was stirred for 24 h at room temperature and subsequently heated for 4 days at 60°C (closed vessel). A green-yellow precipitate suspended in a light green supernatant solution was obtained. All volatiles were removed under reduced pressure and to the remaining solid CH_2Cl_2 (approx. 100 mL) was added. The resulting suspension was filtered and the clear CH_2Cl_2 solution was concentrated under reduced pressure and subsequently layered with *n*-hexane. After 24 h yellow prisms started to form. The crystals were decanted from the mother liquor, washed with *n*-hexane and Et_2O, and dried in a stream of argon. The concentrated mother liquor was layered again with *n*-hexane and a second batch of crystals was obtained, which was treated equally. The crystallization was repeated a third time as described above to give **3** (105 mg, 60%) as a yellow crystalline material. Compound **3** is stable to air and moisture, fairly soluble in CH_2Cl_2, and partially soluble in toluene, $CHCl_3$, and MeCN. **Mp**. 222–225°C (dec.). **^{1}H NMR (400 MHz, CD_2Cl_2, 300 K)**: δ = 7.53 – 7.40 (m, 24H, P*Ph*$_2$), 7.40 – 7.26 (m, 16H, P*Ph*$_2$), 7.03 – 6.92 (m, 4H, H3 and H5), 6.95 – 6.85 ppm (m, 2H, H4). **^{13}C NMR (101 MHz, CD_2Cl_2, 300 K)**: δ = 189.95 (ddd, $^2J(^{13}C–^{31}P)$ = 106.7, $^3J(^{13}C–^{31}P)$ = 49.9, $^3J(^{13}C–^{31}P)$ = 43.4 Hz, C1), 145.62 (dd, J = 67.4, 25.4 Hz C2 or C6), 140.88 – 139.22 (m, C2 or C6), 135.31 – 134.70 (multiple resonances overlapped: 2×CH of P*Ph*$_2$, and C5 or C3), 134.05 (d, $^3J(^{13}C–^{31}P)$ = 14.6 Hz, C4), 131.85 (s, *p*-CH of P*Ph*$_2$), 131.26 (d, $^1J(^{13}C–^{31}P)$ = 56.4 Hz, *i*-C of P*Ph*$_2$), 131.21 (s, *p*-CH of P*Ph*$_2$) 130.91 (dd, $^1J(^{13}C–^{31}P)$ = 49.6, $^5J(^{13}C–^{31}P)$ = 2.4 Hz, *i*-C of P*Ph*$_2$), 129.54 – 129.00 (multiple resonances overlapped: 2×CH of P*Ph*$_2$), 126.78 – 126.51 ppm (m, C3 or C5). **^{31}P NMR (162 MHz, CD_2Cl_2, 300 K)**: δ = 36.59 (s), 36.04 (s) ppm. HRMS-ESI (*m*/*z*): $[M−Cl]^+$ calcd for $C_{60}H_{46}Au_4ClP_4$, 1713.08951; found, 1713.08618.

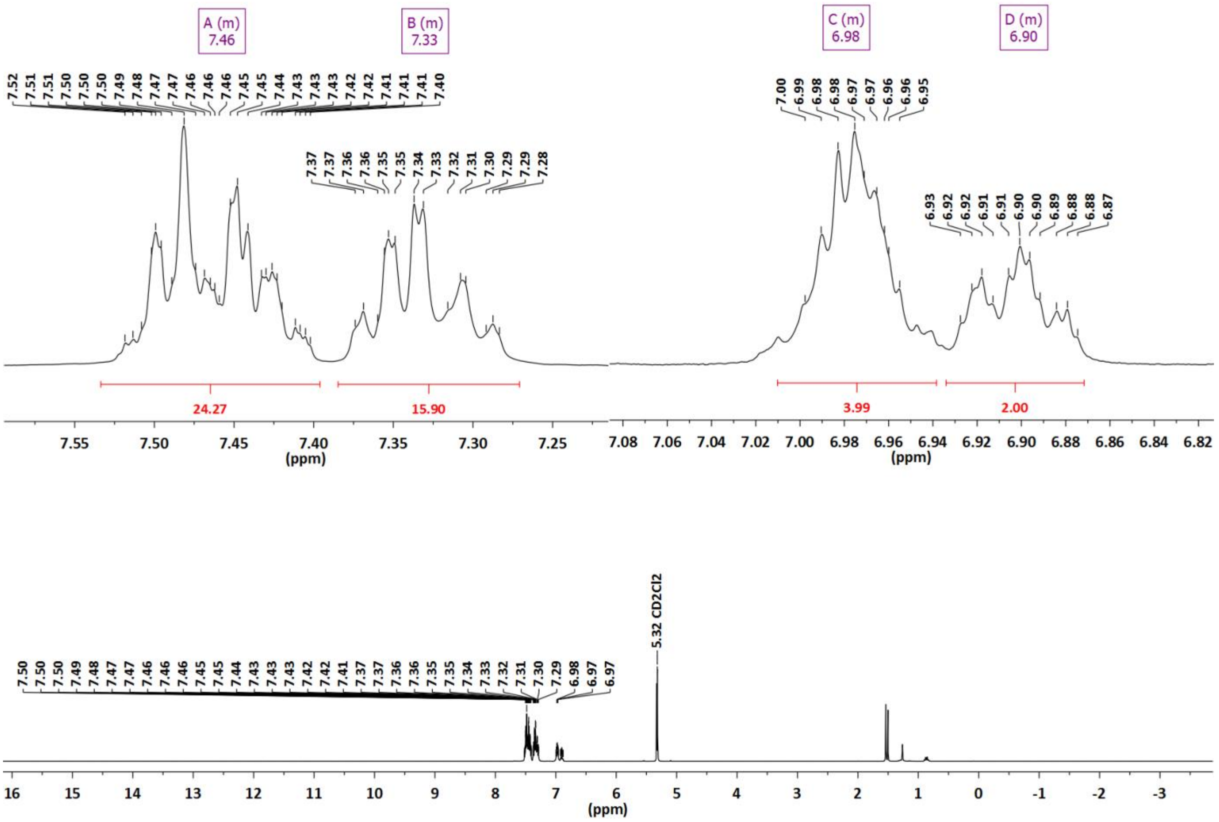

Figure S9: ^{1}H NMR (CD_2Cl_2, 400 MHz) spectrum of **3**.

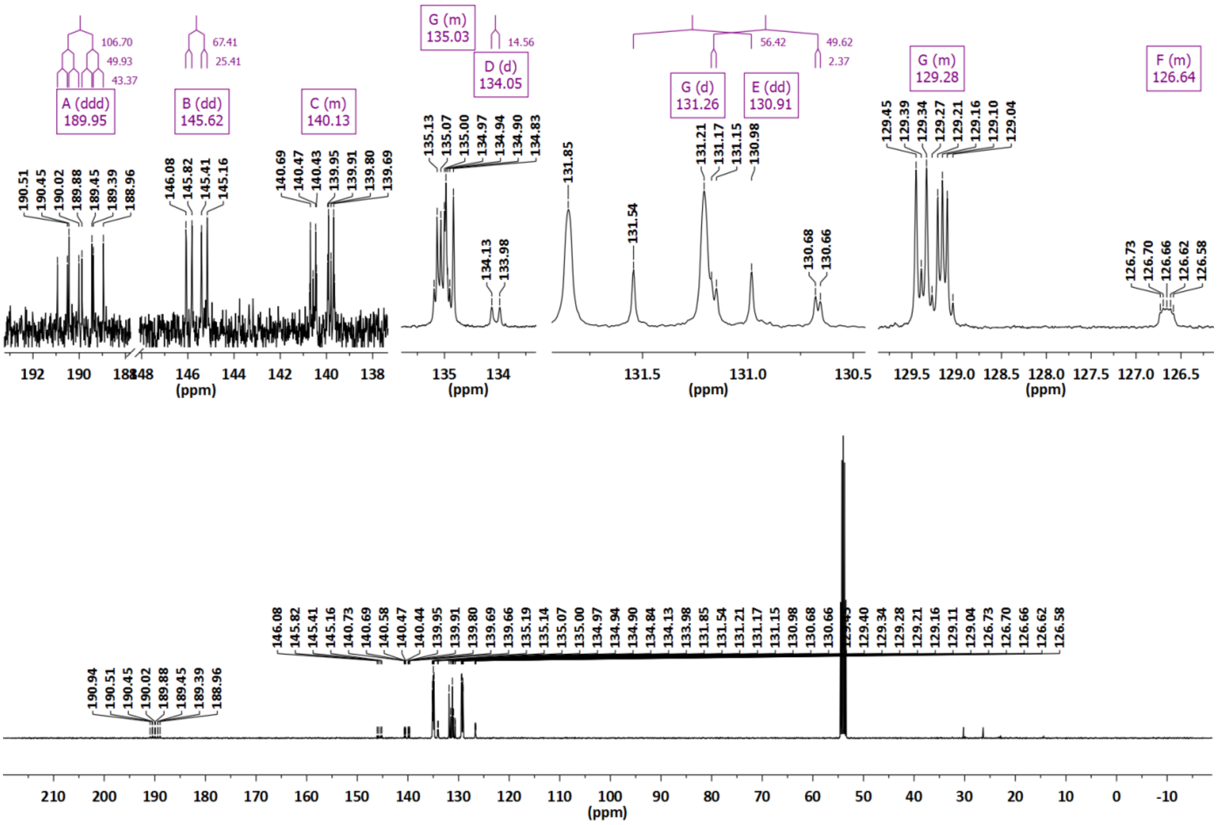

Figure S10: ^{13}C NMR (CD_2Cl_2, 101 MHz) spectrum of **3**.

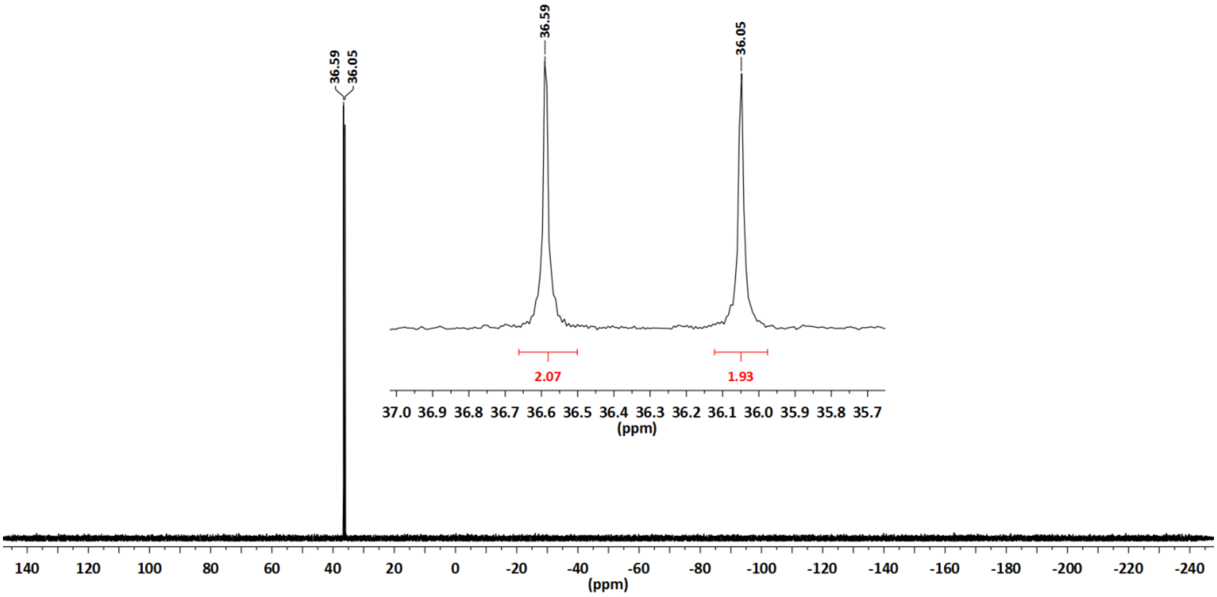

Figure S11: ^{31}P NMR (CD_2Cl_2, 162 MHz) spectrum of **3**.

Synthesis and characterisation of 4

An NMR tube was charged with **1** (17 mg, 0.0097 mmol, 1 eq) and $[NaBAr^F_4]\cdot(H_2O)_3$ (18 mg, 0.0191 mmol, 2 eq). A mixture the of CD_2Cl_2 (0.6 mL) and tht (2 mg, 2 μL, 0.022 mmol, 2.2 eq) was added, the NMR tube was shaken for approx. 5 minutes in the dark then introduced in the NMR. Complete conversion of **3** into **4** was observed by ^{31}P NMR. **^{1}H NMR (400 MHz, CD_2Cl_2, 300 K)**: δ = 7.75 – 7.70 (m, 16H, BAr^F_4), 7.66 – 7.49 (m, 17H, P*Ph*$_2$), 7.55 (s, 8H, BAr^F_4), 7.47 – 7.38 (m, 16H, P*Ph*$_2$), 7.31 – 7.21 (br, m, 7H, P*Ph*$_2$), 7.10 (br, apparent dd, *J* = 9.1, 6.2 Hz, 2H, H4), 6.95 (br, apparent t, *J* = 7.9 Hz, 4H, H3 and H5), 2.87 – 2.78 (br, m, 8H, S-C*H*$_2$-, tht), 1.99 – 1.87 ppm (m, 8H, -C*H*$_2$-, tht). **^{13}C NMR (101 MHz, CD_2Cl_2, 300 K)**: δ = 190.74 – 188.18 (m, C1), 162.3 (q, $^1J(^{11}B–^{13}C)$ = 49.9 Hz, sept, $^1J(^{10}B–^{13}C)$ = 16.7 Hz, C_{BArF}), 146.36 – 144.87 (m, C2 or C6), 138.34 – 136.78 (m, C2 or C6), 136.07 – 134.16 (multiple signals overlapped: C3, C5, C_{BArF}, multiple C of P*Ph*$_2$), 133.17 (s, *p*-CH, PPh_2), 132.55 (s, *p*-CH, PPh_2), 130.52 – 128.53 (multiple signals overlapped: C_{BArF}, multiple C of P*Ph*$_2$), 128.39 – 128.05 (br, m, C4), 125.42 (q, $^1J(^{19}F–^{13}C)$ = 272.5 Hz, C_{BArF}), 118.07 (apparent quintet, $^3J(^{19}F–^{13}C)$ = 4.1 Hz, CH, C_{BArF}), 39.29 (s, S-CH_2-, tht), 31.36 ppm (s, -CH_2-, tht). **^{31}P NMR (162 MHz, CD_2Cl_2, 300 K)**: δ = 39.48 (s), 35.14 ppm (s). **^{19}F NMR (376 MHz, CD_2Cl_2, 300 K)**: δ =−62.8 ppm (s).

After the ^{1}H, ^{13}C, ^{19}F, ^{31}P, COSY, HSQC, and HMBC spectra were measured (which required 20 h) another ^{31}P NMR spectrum was recorded. After 20 h in the dark approx. 2% molar of **5** and approx. 5% molar of an unidentified by-product were found aside from **4** (Figure S16).

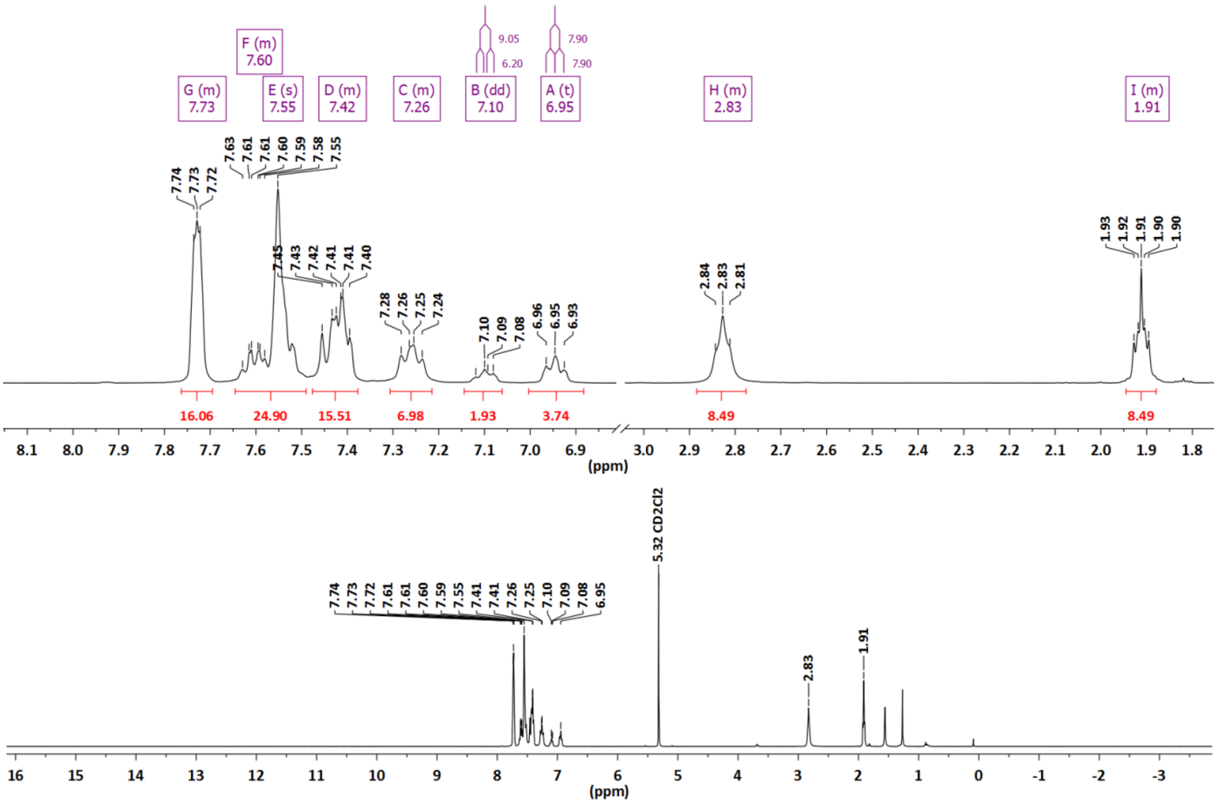

Figure S12: ^{1}H NMR (CD_2Cl_2, 400 MHz) spectrum of **4**.

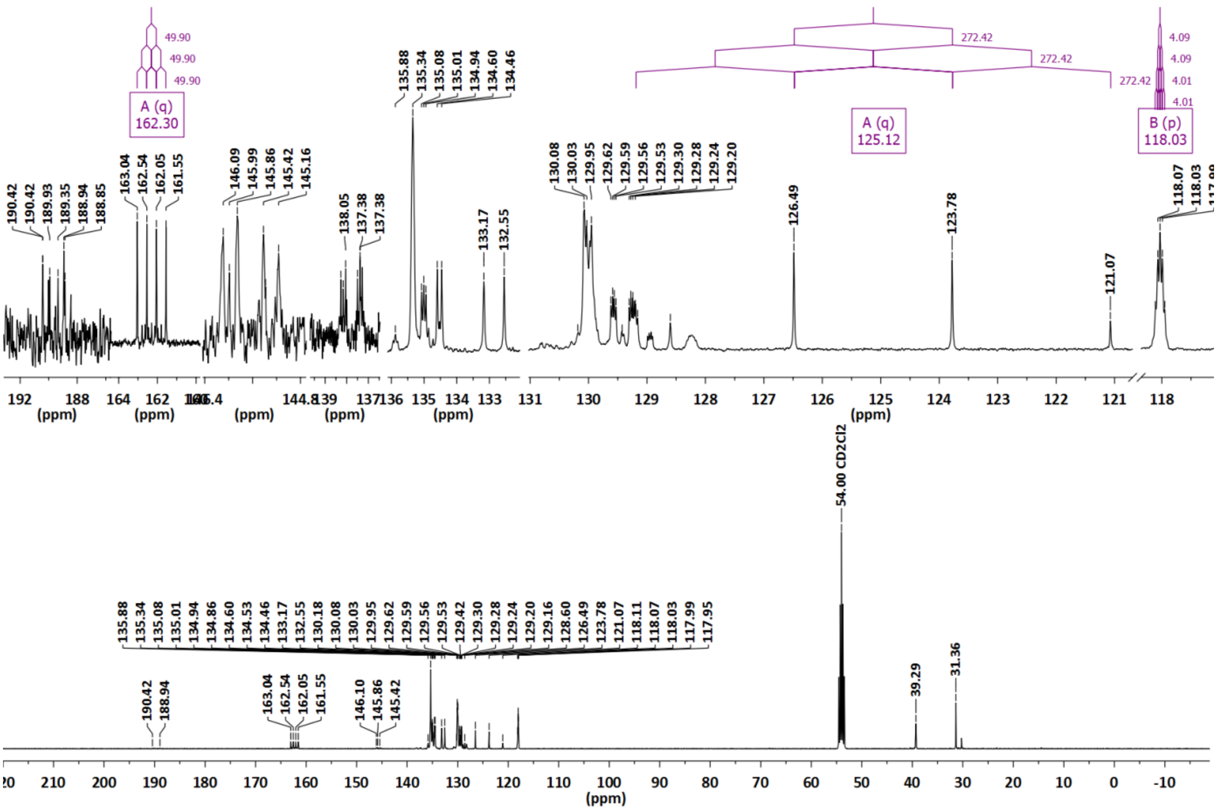

Figure S13: ^{13}C NMR (CD_2Cl_2, 101 MHz) spectrum of **4**.

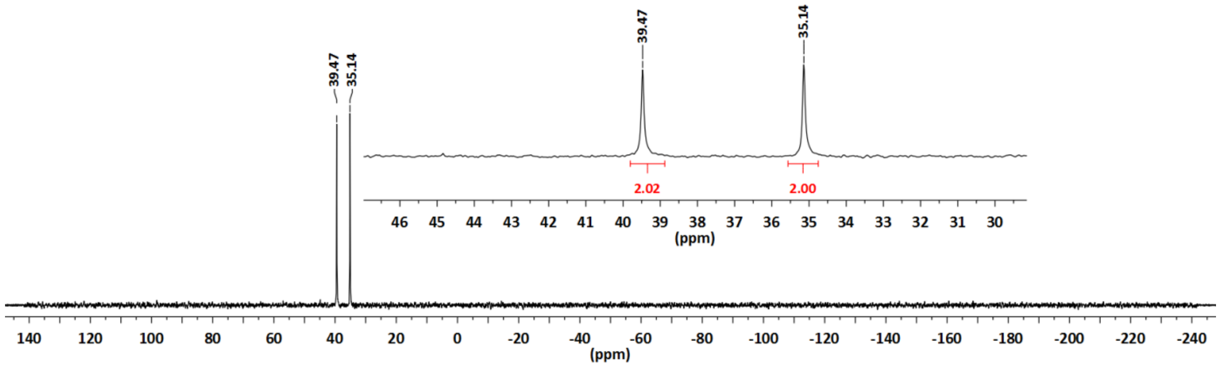

Figure S14: ^{31}P NMR (CD_2Cl_2, 162 MHz) spectrum of **4**.

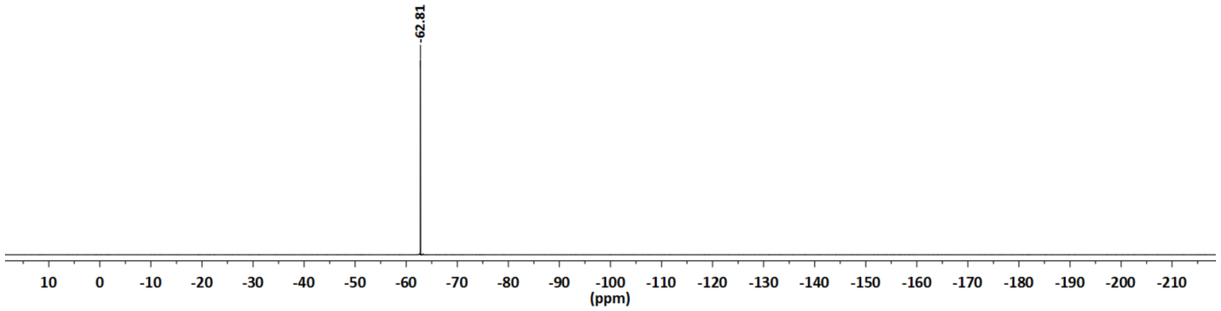

Figure S15: ^{19}F NMR (CD_2Cl_2, 376 MHz) spectrum of **4**.

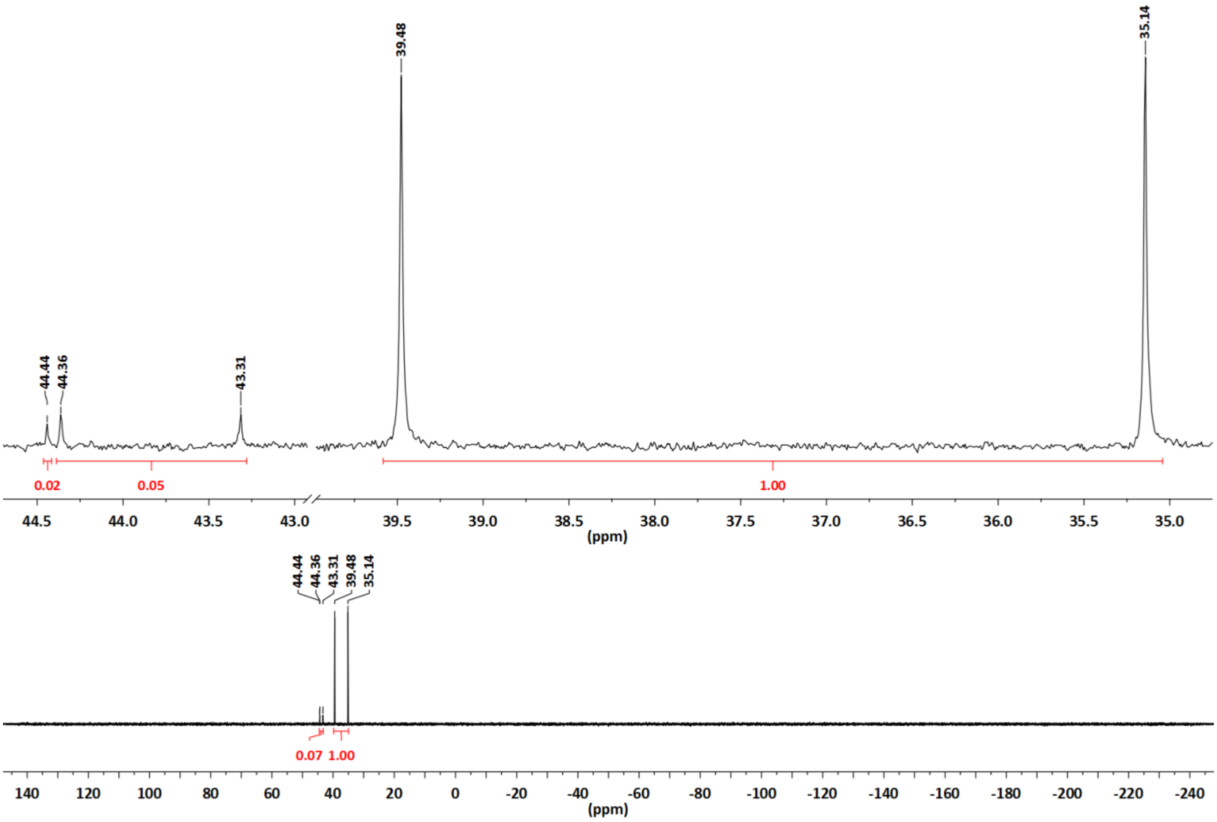

Figure S16: ^{19}F NMR (CD_2Cl_2, 162 MHz) spectrum of **4** after 20h in the dark.

Isolation of single crystals of 4 suitable for an X-ray diffraction study

In the glovebox, a vial was charged with complex **3** (77 mg, 0.044 mmol) and pre-dried $NaBAr^{F}_{4}$ (78 mg, 0.088 mmol). The vial was taken out of the glovebox and with protection from ambient light, CH_2Cl_2 (3 mL) and tht (20 μL, 20 mg, 0.22 mmol) were added. The mixture was stirred for 25 minutes then filtered through a syringe filter (PTFE membrane). The vial containing the filtrate solution was placed in a larger brown bottle and *n*-hexane vapours were allowed to diffuse into the CH_2Cl_2 solution for 12 h. A resin formed at the bottom of the vial as most of the CH_2Cl_2 evaporated during this time. The residue was washed with heptane (4×5 mL) to obtain a semi-solid paste. Addition of $CHCl_3$ (2 mL) caused the formation of a suspension. To this suspension CH_2Cl_2 was added (approx. 4 mL) giving rise to a clear solution, which was subsequently layered with heptane (20 mL). Crystallization commenced within hours and after approx. 20 h the supernatant solution was separated. Two types of crystals were present in the vial (see Figure S17): blocks (which proved to be compound **5**) and needles (which proved to be compound **4**). More needles formed later from the supernatant solution as the more volatile halogenated solvents evaporated slowly.

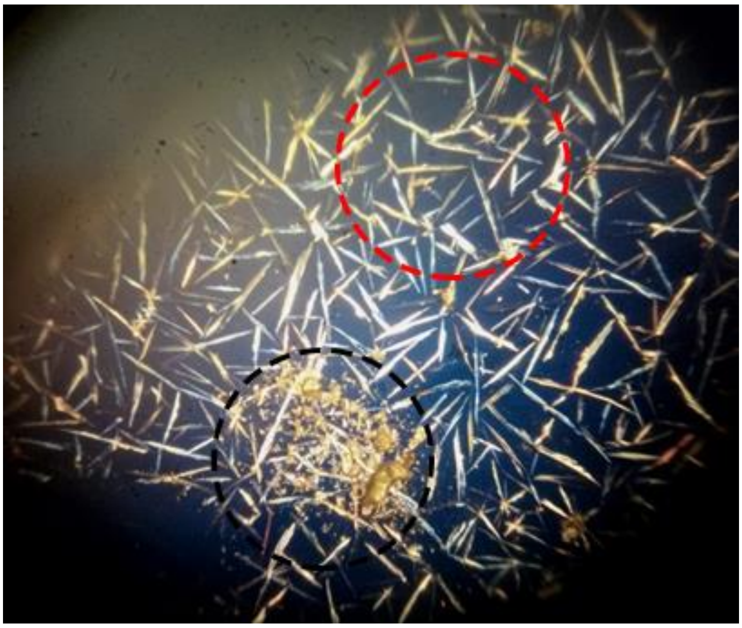

Figure S17. Photograph of the crystals of **4** (red circle) and **5** (black circle).

Synthesis and characterisation of 5

$NaBAr^F_4$ (20 mg, 0.22 mmol) was added to a solution of **1** (20 mg, 0.11 mmol) in CD_2Cl_2 (0.5 mL) in a *J. Young* NMR tube. The NMR tube was protected from ambient light and tht (3 drops) was added. A $^{31}P\{^1H\}$ NMR spectrum was recorded immediately. Compound **4** was the major product present (see supporting information); traces of **5** and another minor by-product were also observable. The NMR tube was exposed to UV light (λ_{max}= 366 nm). The initial yellow-reddish solution became dark green immediately after exposure to the UV light. After 35 minutes of irradiation, complex **4** was completely converted. The NMR tube was stored 12 h in the dark and the solution became pale yellow but it was still turning green upon exposure to UV- or ambient light. After 48 h, when the solution was not responding to light exposure anymore, all volatiles were removed under reduced pressure and the residue re-dissolved in CD_2Cl_2. The $^{31}P\{^1H\}$ NMR spectrum indicated the formation of **5** as the major product (ca. 76%) and another minor impurity. Compound **5** was purified and isolated as colourless crystals after recrystallization from CH_2Cl_2 / petroleum ether (1:2) in 20–30% yield. Compound **5** is stable to air and moisture. **M.p.** 218°C (dec., becomes grey without melting). **^{1}H NMR (400 MHz, CD_2Cl_2, 300 K)**: δ = 7.75 – 7.70 (br, m, 16H, BAr^F_4), 7.67 – 7.42 (m, 40H, P*Ph*$_2$), 7, 54 (s,8H, BAr^F_4) 7.18 – 7.10 (m, 2H, H4), 6.98 – 6.89 (m, 4H, H3), 2.13 (br, 8H, S-C*H*$_2$-, tht), 1.08 ppm (br, 8H, -C*H*$_2$-, tht). **^{13}C NMR (101 MHz, CD_2Cl_2, 300 K)**: δ = 179.13 (t, $^2J(^{13}C–^{31}P)$ = 21.5 Hz, C1), 162.3 (q, $^1J(^{11}B–^{13}C)$ = 49.8 Hz, sept, $^1J(^{10}B–^{13}C)$ = 16.7 Hz, C_{BArF}), 139.03 (tt, $^1J(^{13}C–^{31}P)$ = 37.0, $^3J(^{13}C–^{31}P)$ = 10.8 Hz, C2), 136.25 (apparent t, *J* = 7.2 Hz, C3), 135.33 (br, s, C_{BArF}), 134.89 (t, *J* = 7.8 Hz, CH of P*Ph*$_2$), 134.33 (t, *J* = 6.6 Hz, CH of P*Ph*$_2$), 133.63 (s, *p*-CH of P*Ph*$_2$), 133.02 (s, *p*-CH of P*Ph*$_2$), 130.92 (t, *J* = 30.1 Hz, *i*-C of P*Ph*$_2$), 130.62 (t, *J* = 5.7 Hz, CH of P*Ph*$_2$), 130.23 (t, *J* = 5.7 Hz, CH of P*Ph*$_2$), 129.41 (qq, $^2J(^{19}F–^{13}C)$ = 31.5 Hz, $^4J(^{19}F–^{13}C)$ = 2.7 Hz, C_{BArF}), 128.42 – 128.08 (m, C4), 127.38 (t, *J* = 25.1 Hz, *i*-C of P*Ph*$_2$), 125.12 (q, $^1J(^{19}F–^{13}C)$ = 272.5 Hz, C_{BArF}), 118.02 (apparent quintet, $^3J(^{19}F–^{13}C)$ = 3.7 Hz, CH, C_{BArF}), 39.96 (s, S-CH$_2$-, tht), 30.70 ppm (s, -CH$_2$-, tht). **^{31}P NMR (162 MHz, CD_2Cl_2, 300 K)**: δ = 44.44 ppm (s). **^{19}F NMR (376 MHz, CD_2Cl_2, 300 K)**: δ = −62.8 ppm (s). **ESI-MS** (MeCN, negative mode): *m/z*: 863.0 ($C_{36}H_8F_{36}B$) for $[B\{3,5\text{-}(CF_3)_2C_6H_3\}_4]^-$. HRMS-ESI (*m/z*): $[M - 2(tht)]^{2+}$ calcd for $C_{60}H_{46}Au_4P_4$, 839.06005; found, 839.05865.

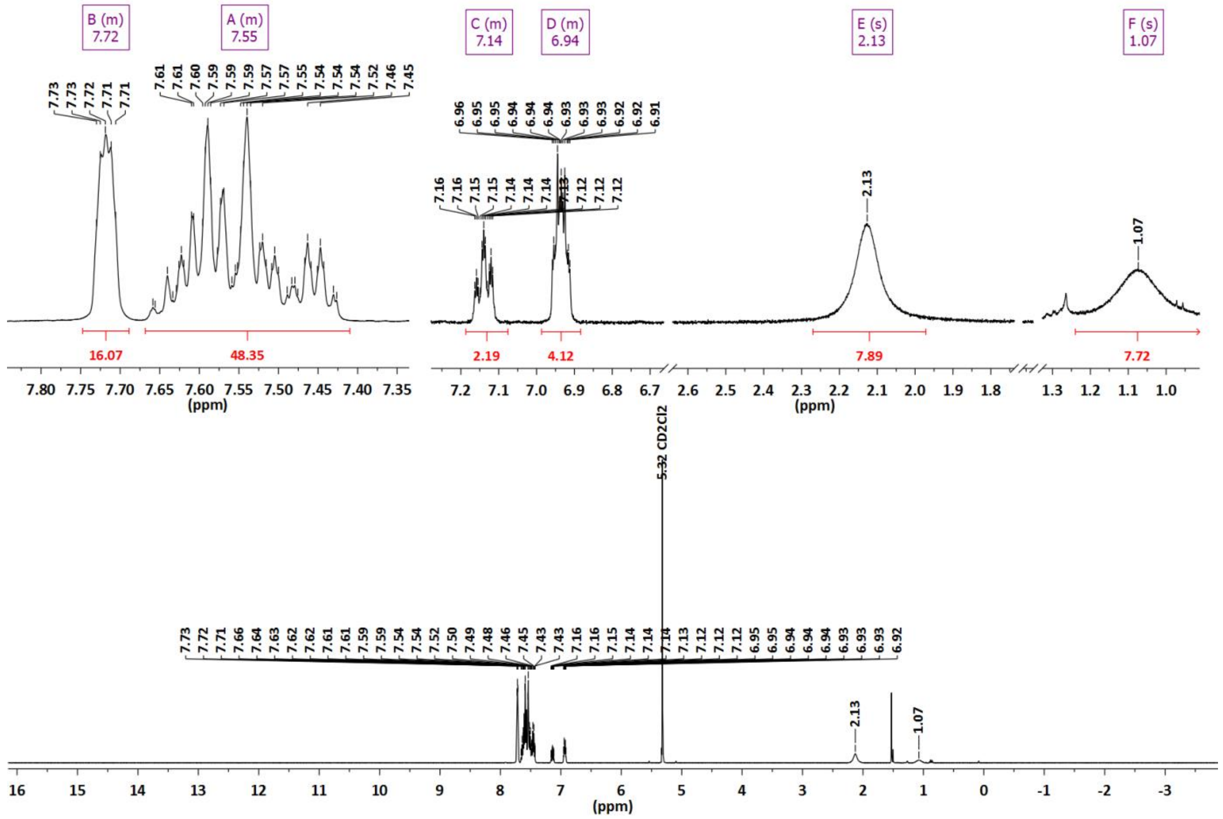

Figure S18: ^{1}H NMR (CD_2Cl_2, 400 MHz) spectrum of **5**.

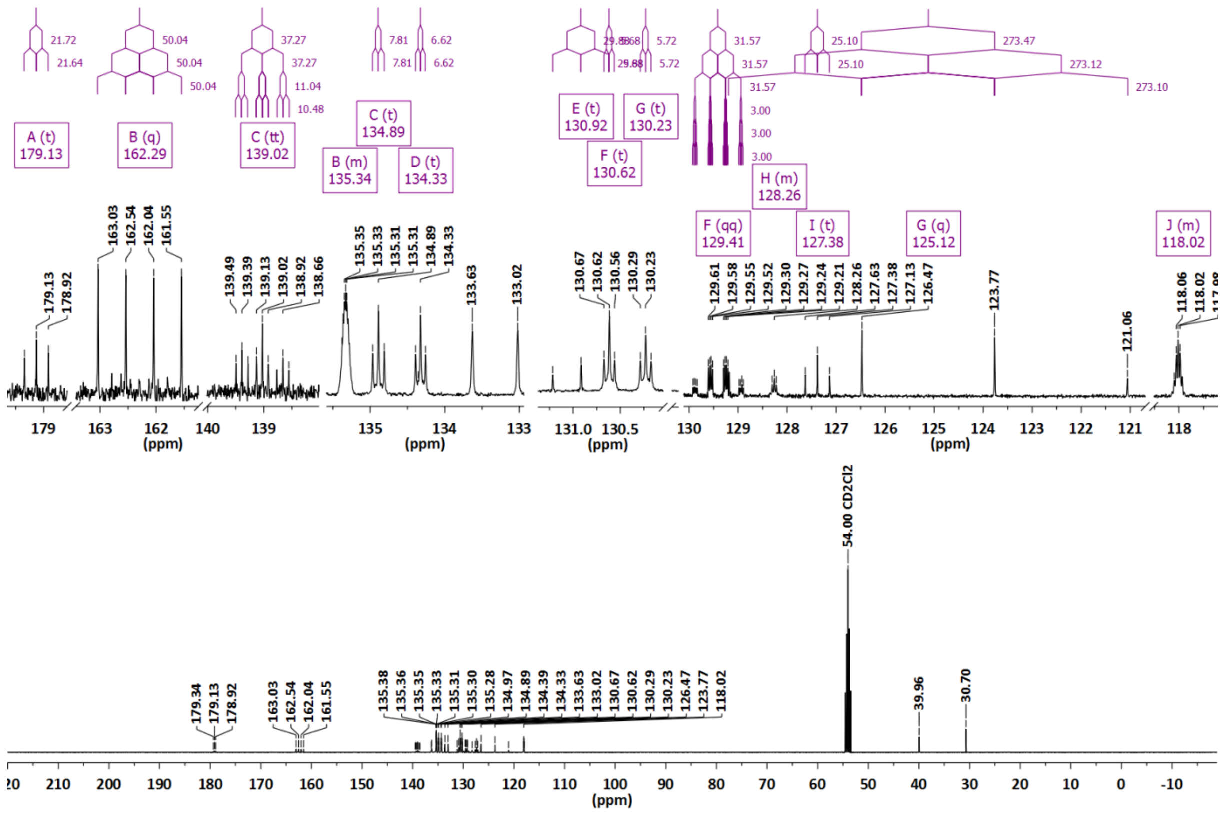

Figure S19: ^{13}C NMR (CD_2Cl_2, 101 MHz) spectrum of **5**.

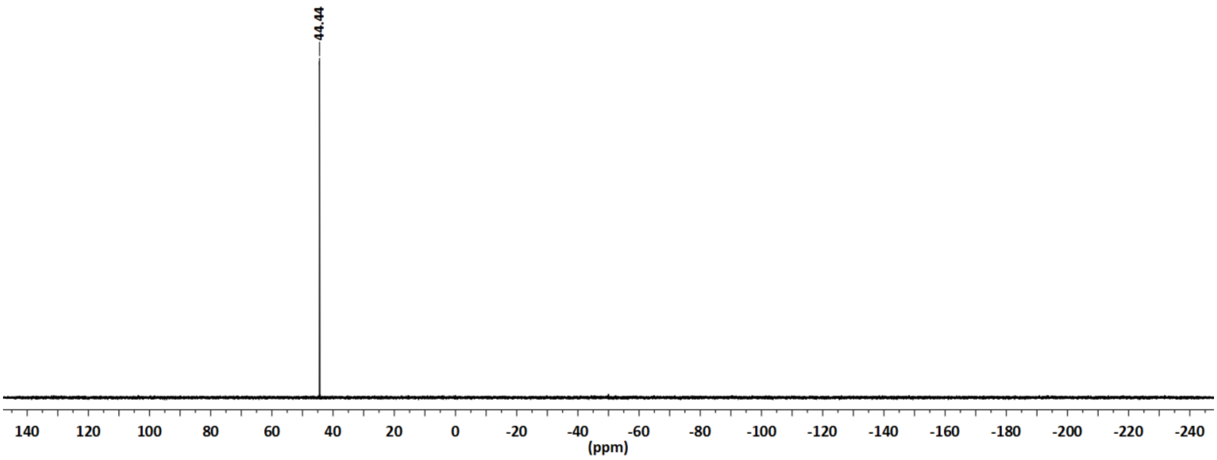

Figure S20: ^{31}P NMR (CD_2Cl_2, 162 MHz) spectrum of **5**.

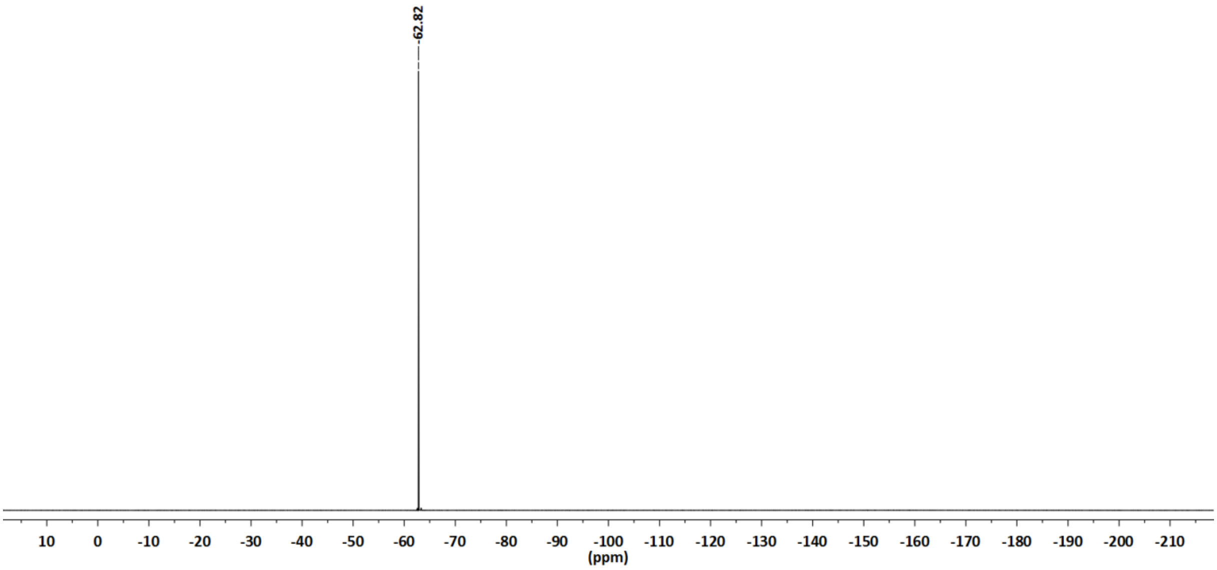

Figure S21: ^{19}F NMR (CD_2Cl_2, 376 MHz) spectrum of **5**.

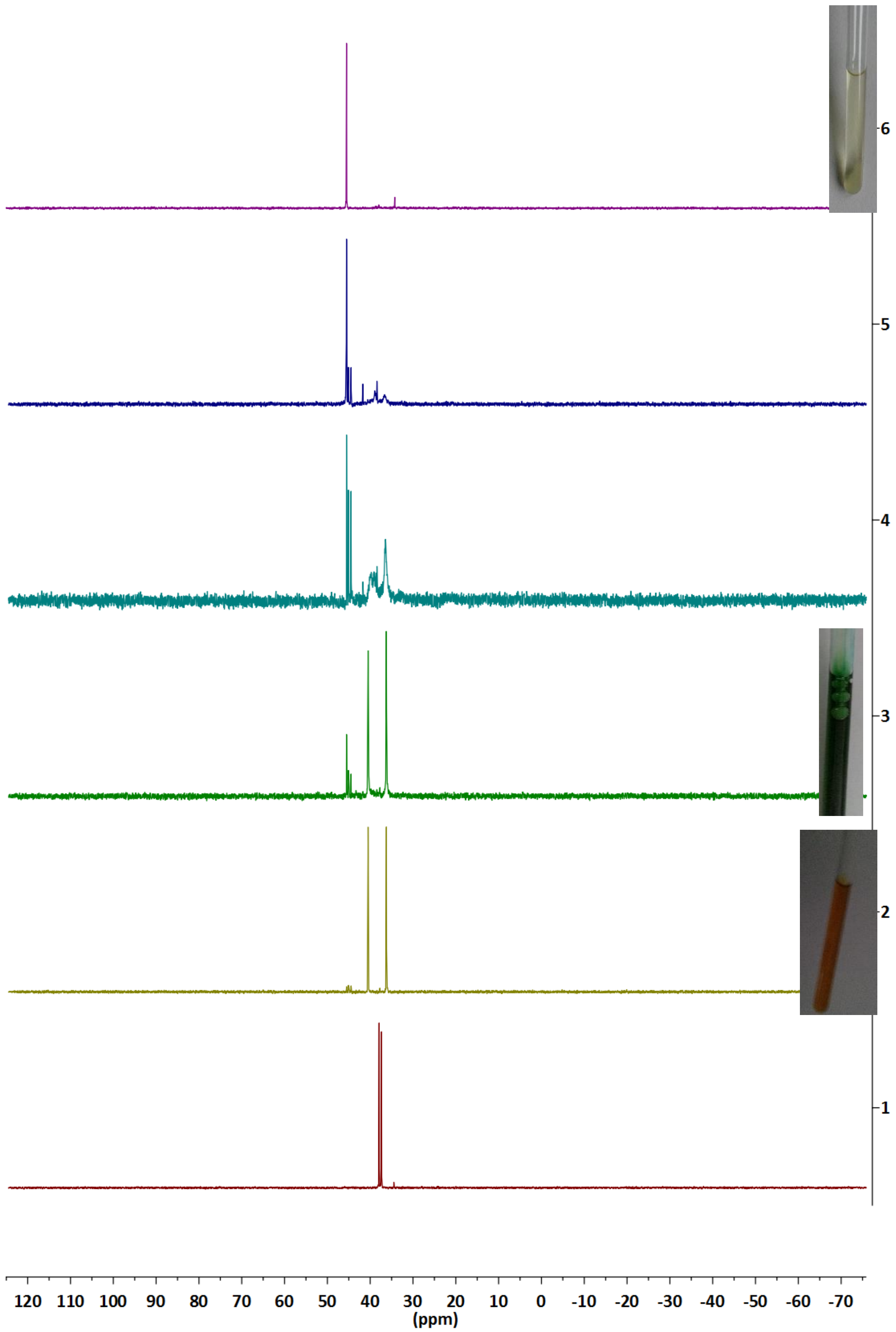

Figure S22: ^{31}P NMR (CD_2Cl_2, 81 MHz) spectra recorded during the conversion of **4** into **5**.
(1) Reference spectrum of **3**. (2) Spectrum recorded shortly after addition of tht to the mixture of **3** and NaBArF in CD_2Cl_2; **3** converts into **4**. (3) Spectrum recorded after exposing the NMR tube contents to UV (366 nm) for 3 min. (4) Spectrum recorded after exposing the NMR tube contents to UV (366 nm) for 30 min. (5) Spectrum recorded 12 h later (the NMR tube was stored in the dark); main signal corresponds to **5**. (6) Spectrum recorded 30 h later after evaporation of all volatiles to remove the excess tht; mixture of **6** and a side-product (integral ration: 1:0.12).

Synthesis and characterisation of 6

In air, to a solid mixture of **1** (60 mg, 0.034 mmol) and $HgCl_2$ (27 mg, 0.1 mmol, 3 equiv.), CH_2Cl_2 was added and the reaction mixture was stirred at ambient temperature for 2 h. A small aliquot of the solution was checked by ^{31}P NMR and complete and clean conversion of **1** into **6** was observed. Excess $HgCl_2$ was removed by filtration through a small plug of Celite, CH_2Cl_2 evaporated to dryness and the resulting solid washed with pentane (2×6 mL) to give **6** as a colourless solid (76 mg, 96%). **Mp.** 218°C (dec.). **^{1}H NMR (400 MHz, CD_2Cl_2, 300 K)**: δ = 7.68 – 7.57 (m, 4H, P*Ph*$_2$), 7.59 – 7.48 (m, 16H, P*Ph*$_2$), 7.38 – 7.32 (m, 1H, H4), 7.23 – 7.17 (m, 2H, H3). **^{13}C NMR (101 MHz, CD_2Cl_2, 300 K)**: δ = 172.11 (t, $^{2}J(^{13}C–^{31}P)$ = 38.8 Hz, C1), 138.62 (dd, $^{1}J(^{13}C–^{31}P)$ = 69.7, $^{3}J(^{13}C–^{31}P)$ = 18.1 Hz, C2), 136.64 (dd, $^{2}J(^{13}C–^{31}P)$ = 11.0, $^{4}J(^{13}C–^{31}P)$ = 2.3 Hz, C3), 135.20 (d, J = 13.7 Hz, CH, P*Ph*$_2$), 133.52 (d, $^{4}J(^{13}C–^{31}P)$ = 2.6 Hz, *p*-CH, P*Ph*$_2$), 130.34 (d, J = 12.0 Hz, CH, P*Ph*$_2$), 130.28 (d, $^{3}J(^{13}C–^{31}P)$ = 16.5 Hz, C4), 127.51 ppm (d, $^{1}J(^{13}C–^{31}P)$ = 60.9 Hz, *i*-C, P*Ph*$_2$). **^{31}P NMR (162 MHz, CD_2Cl_2, 300 K)**: δ = 42.34 (s, $^{3}J(^{31}P–^{199}Hg)$ = 327 Hz). **^{199}Hg NMR (72 MHz, CD_2Cl_2, 300 K)**: δ = –841.5 (t, $^{3}J(^{31}P–^{199}Hg$ = 327 Hz). HRMS-ESI (*m/z*): $[M - Cl]^+$ calcd for $C_{30}H_{23}Au_2Cl_2HgP_2$, 1110.96839; found, 1110.97230.

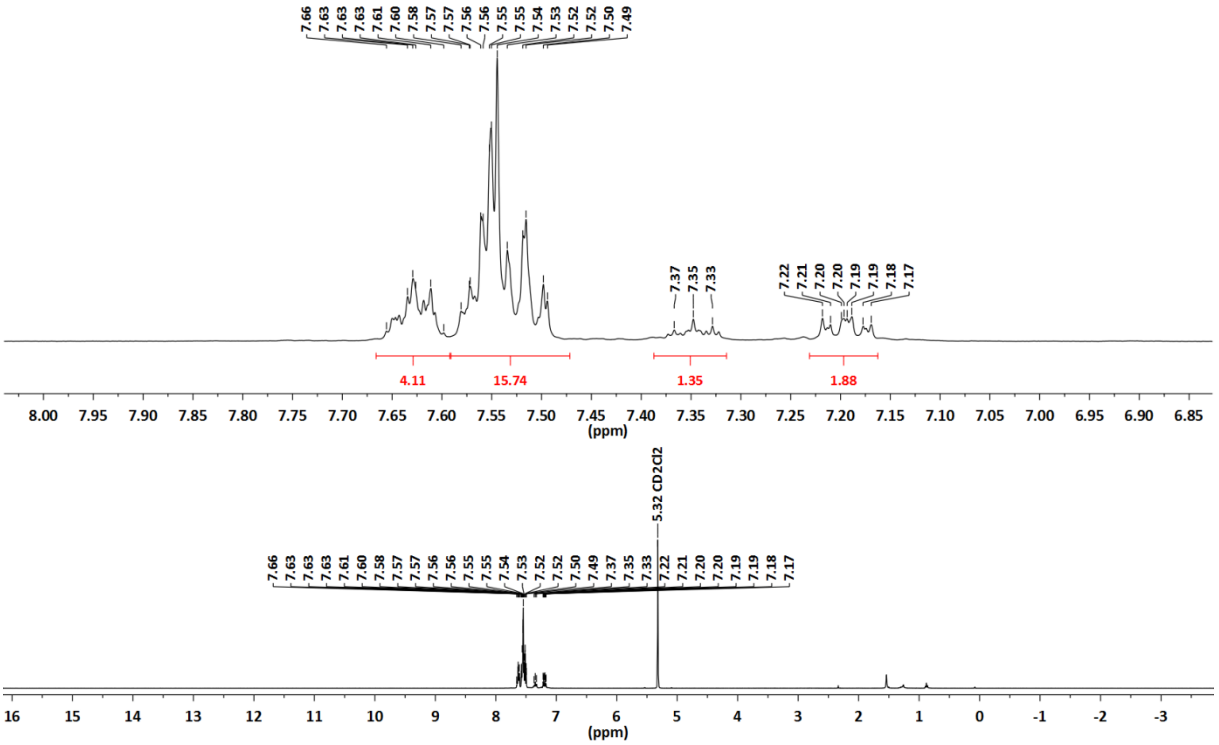

Figure S23: ^{1}H NMR (CD_2Cl_2, 400 MHz) spectrum of **6**.

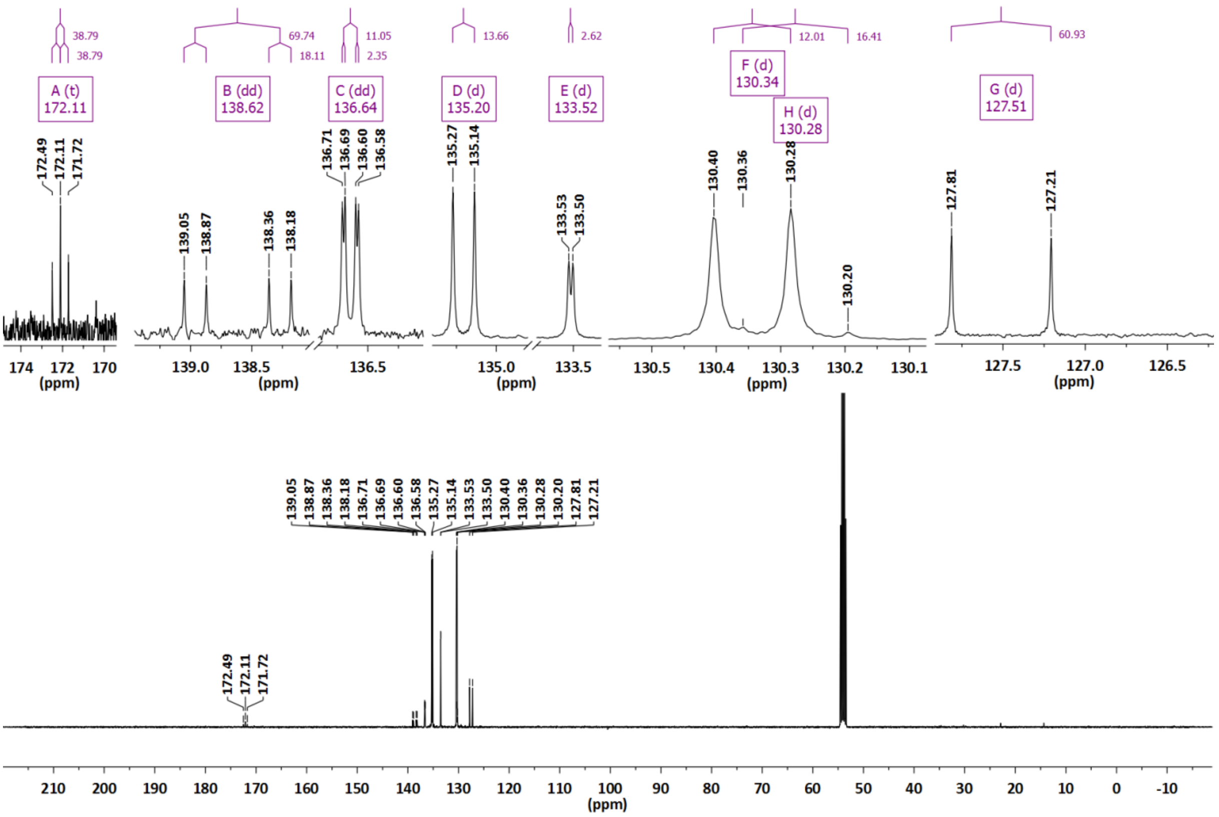

Figure S24: ^{13}C NMR (CD_2Cl_2, 101 MHz) spectrum of **6**.

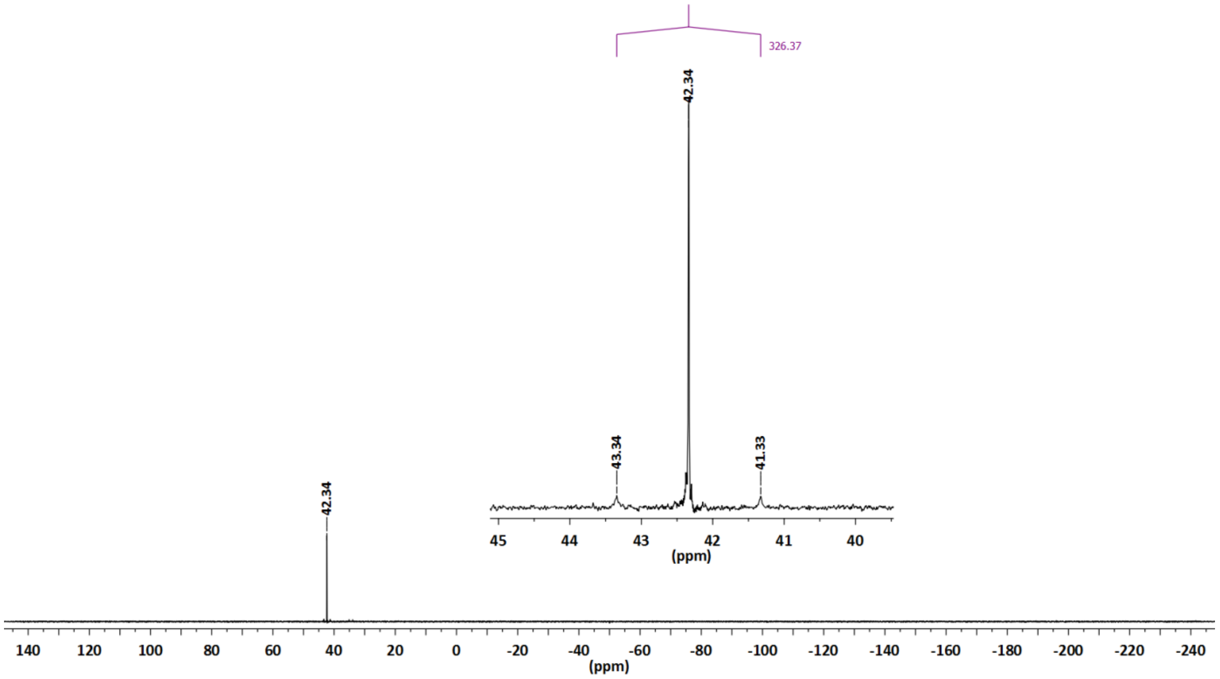

Figure S25: ^{31}P NMR (CD_2Cl_2, 162 MHz) spectrum of **6**.

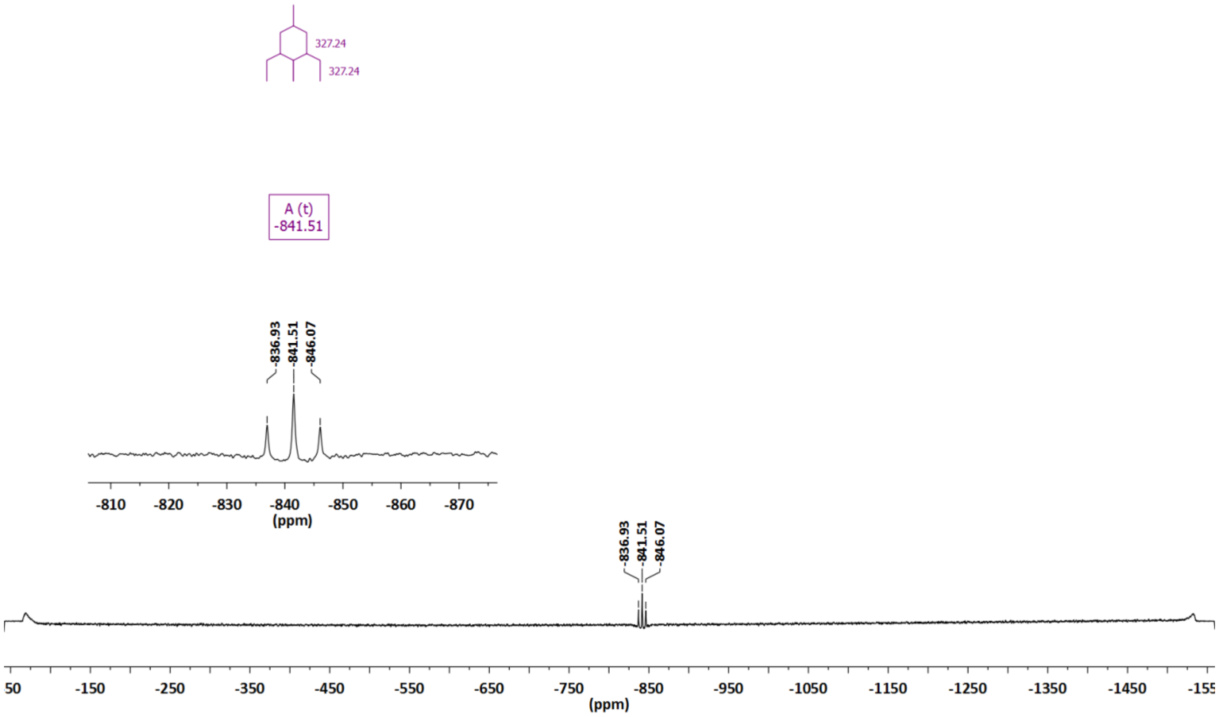

Figure S26: ^{199}Hg NMR (CD_2Cl_2, 72 MHz) spectrum of **6**.

HRMS spectra

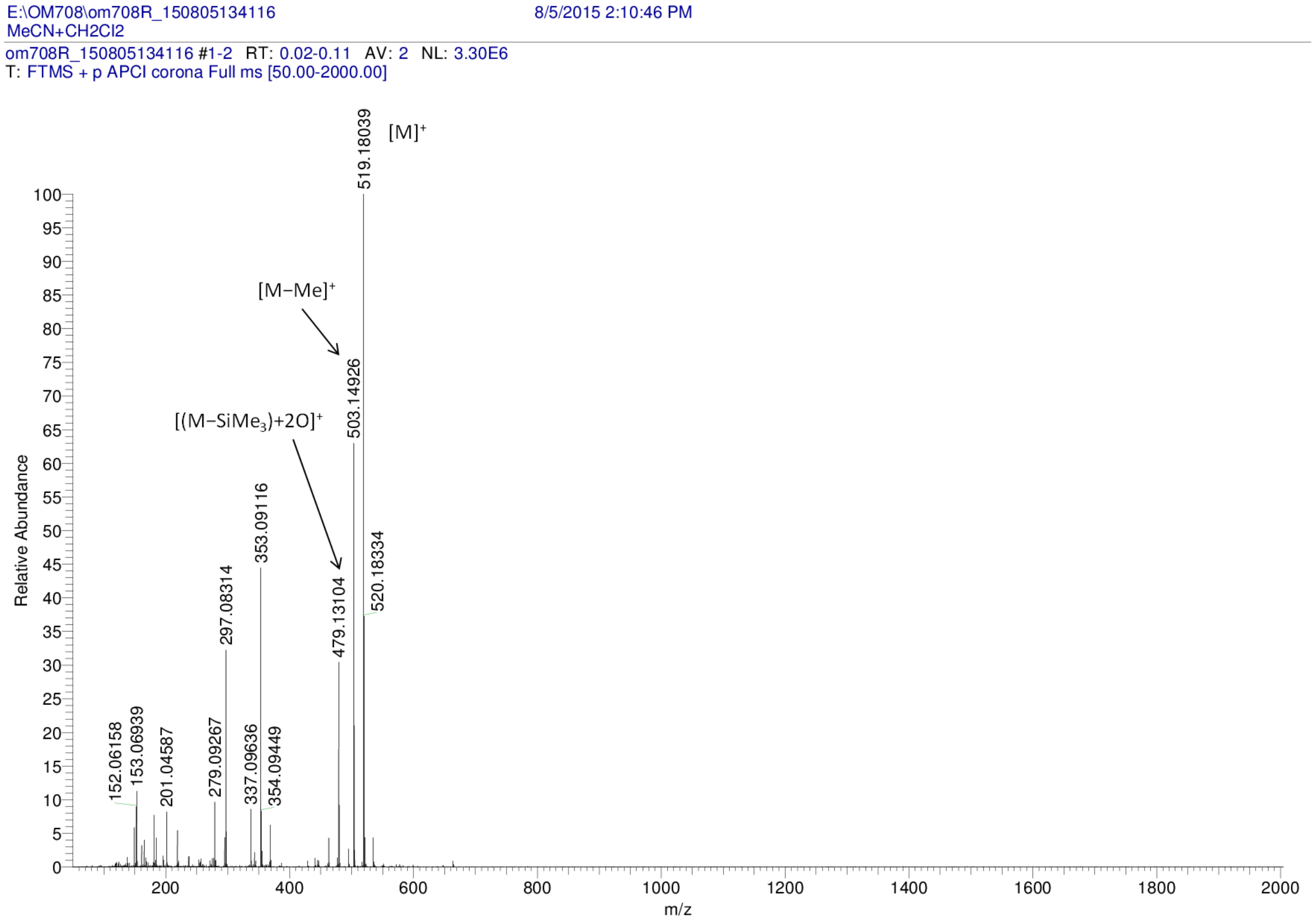

Figure S27: HRMS APCI(+) spectrum of **1**.

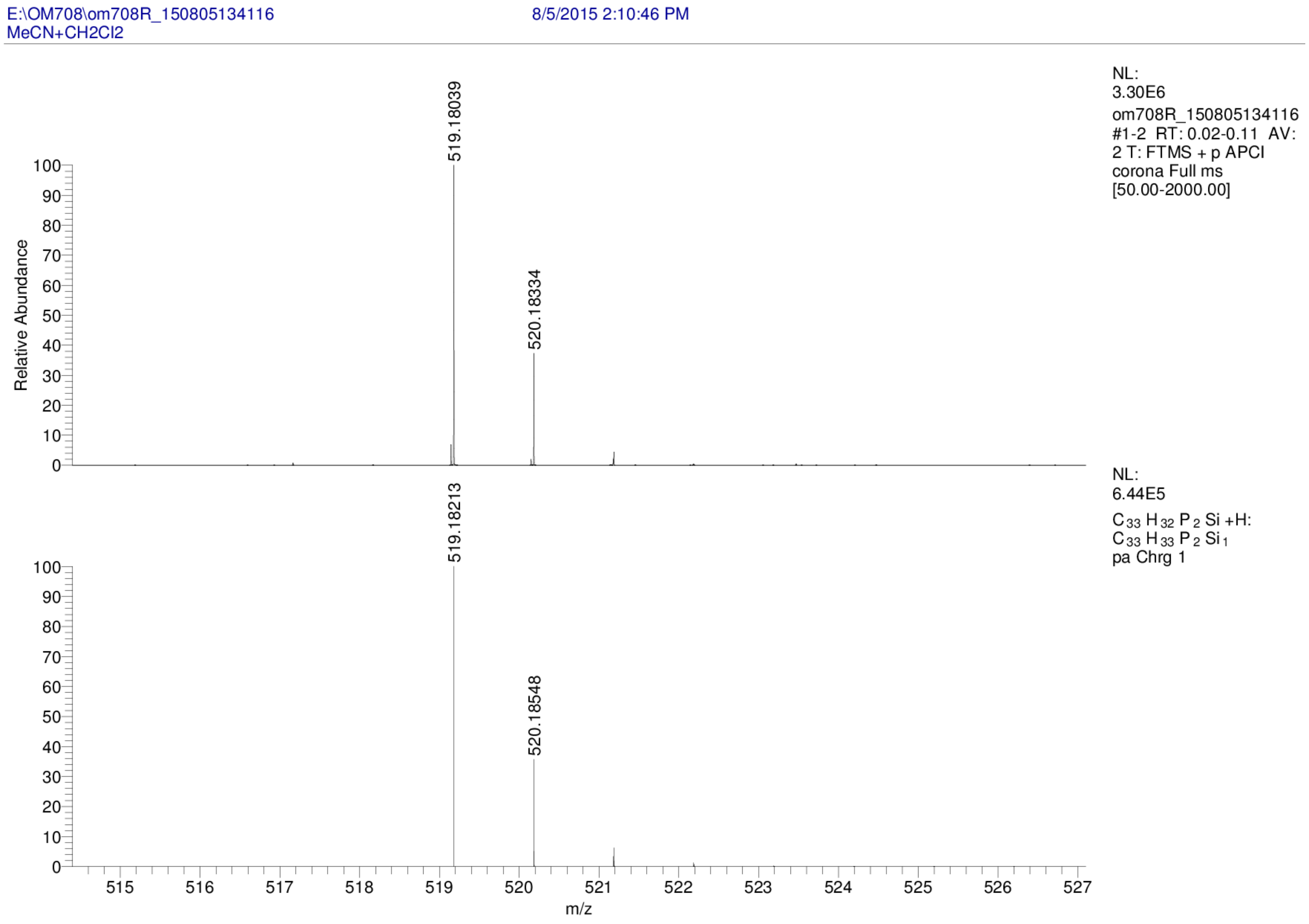

Figure S28: Measured (top) and calculated (bottom) isotopic pattern of $[M]^+$ of **1**.

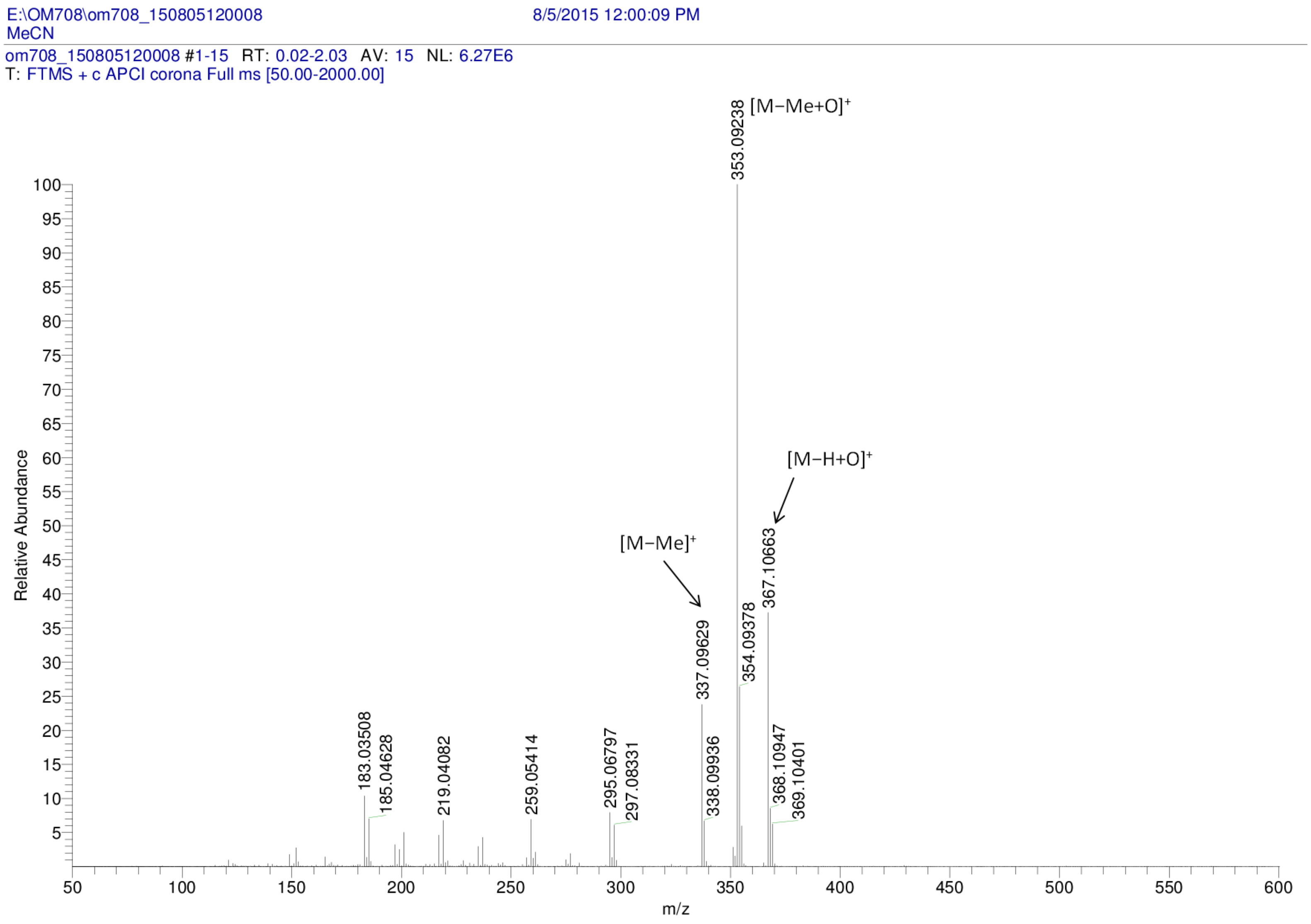

Figure S29: HRMS APCI(+) spectrum of **1a**.

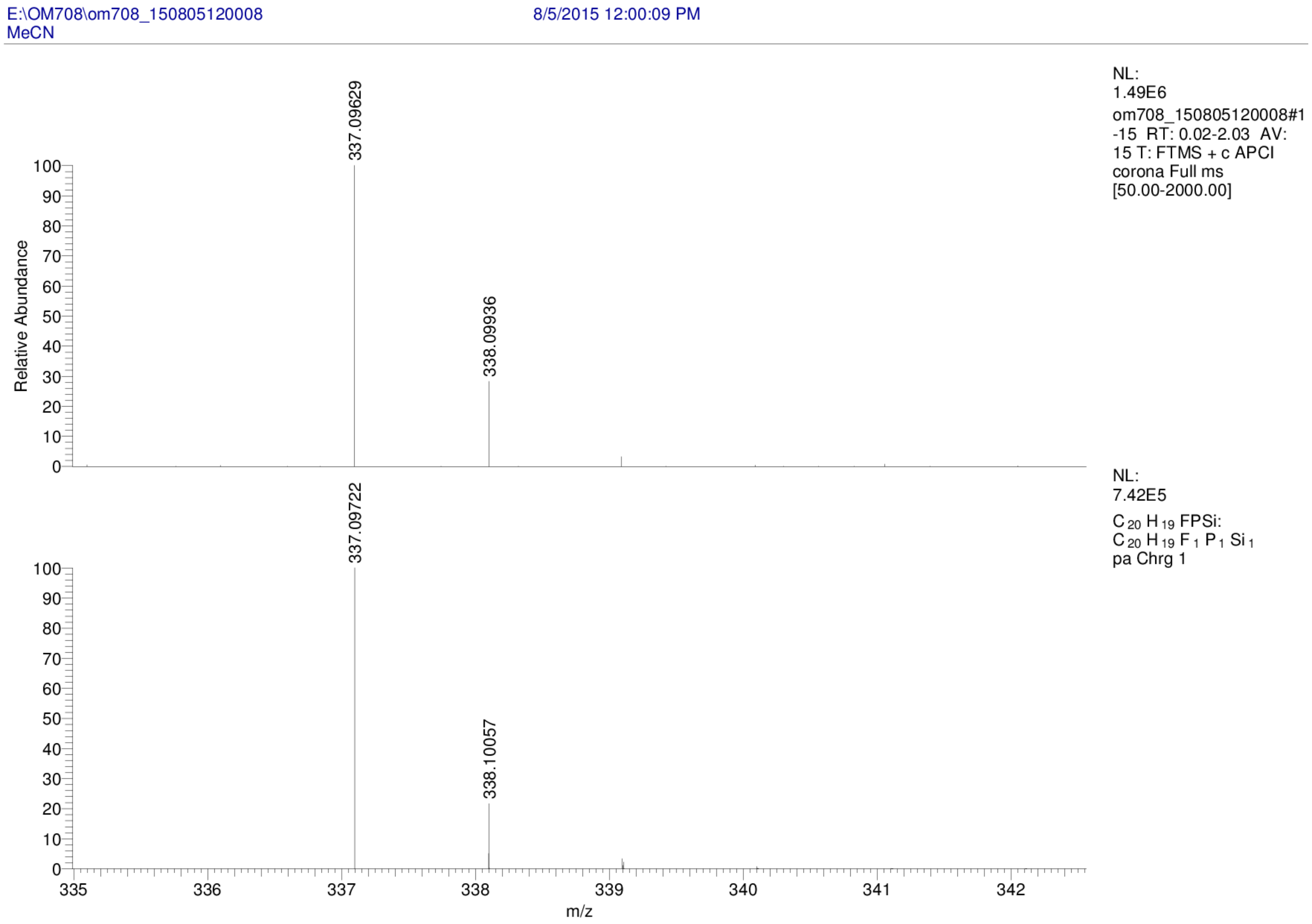

Figure S30: Measured (top) and calculated (bottom) isotopic pattern of $[M\text{-}Me]^+$ of **1a**.

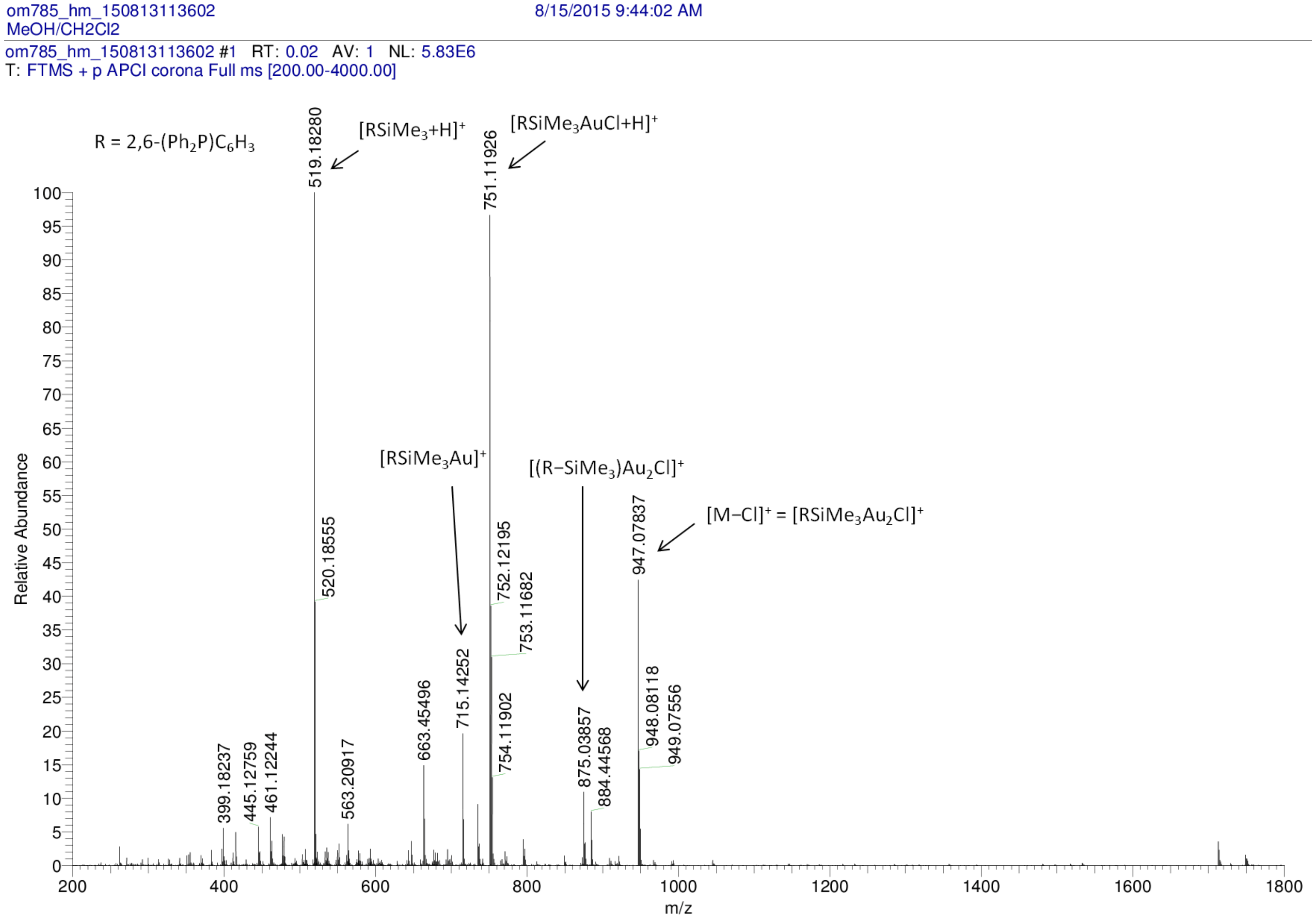

Figure S31: HRMS APCI(+) spectrum of **2**.

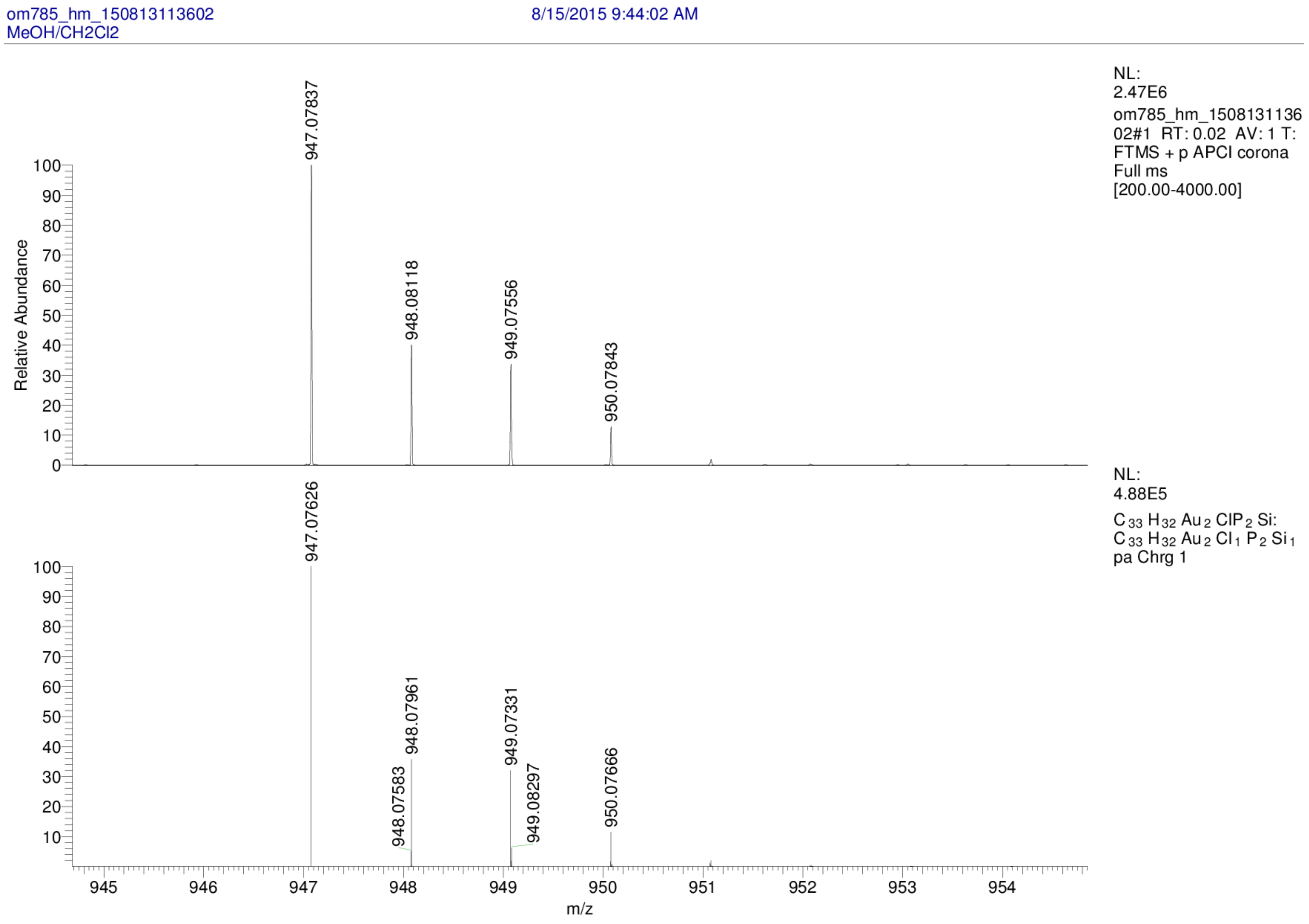

Figure S32: Measured (top) and calculated (bottom) isotopic pattern of $[M{-}Cl]^+$ of **2**.

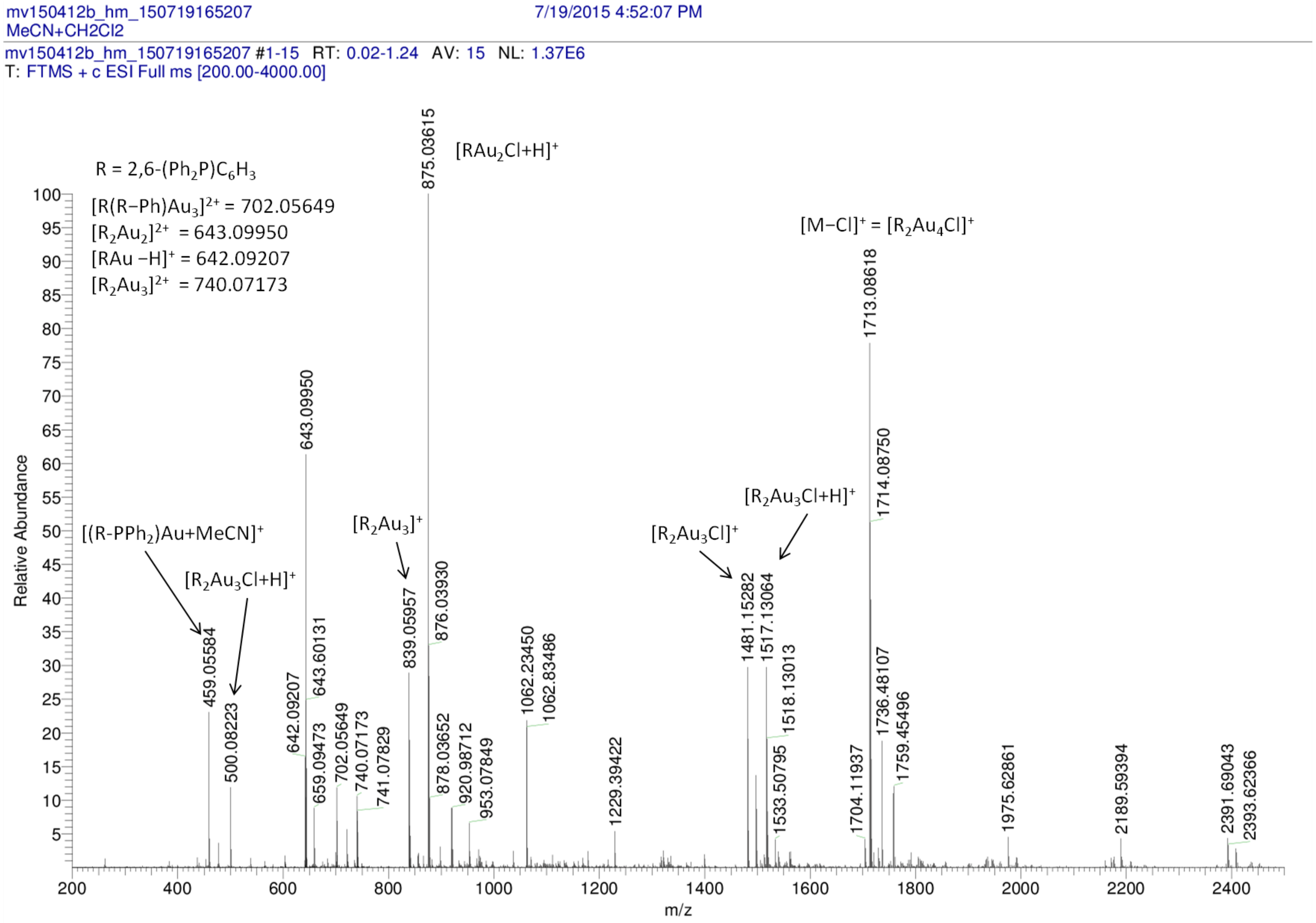

Figure S33: HRMS ESI(+) spectrum of **3**.

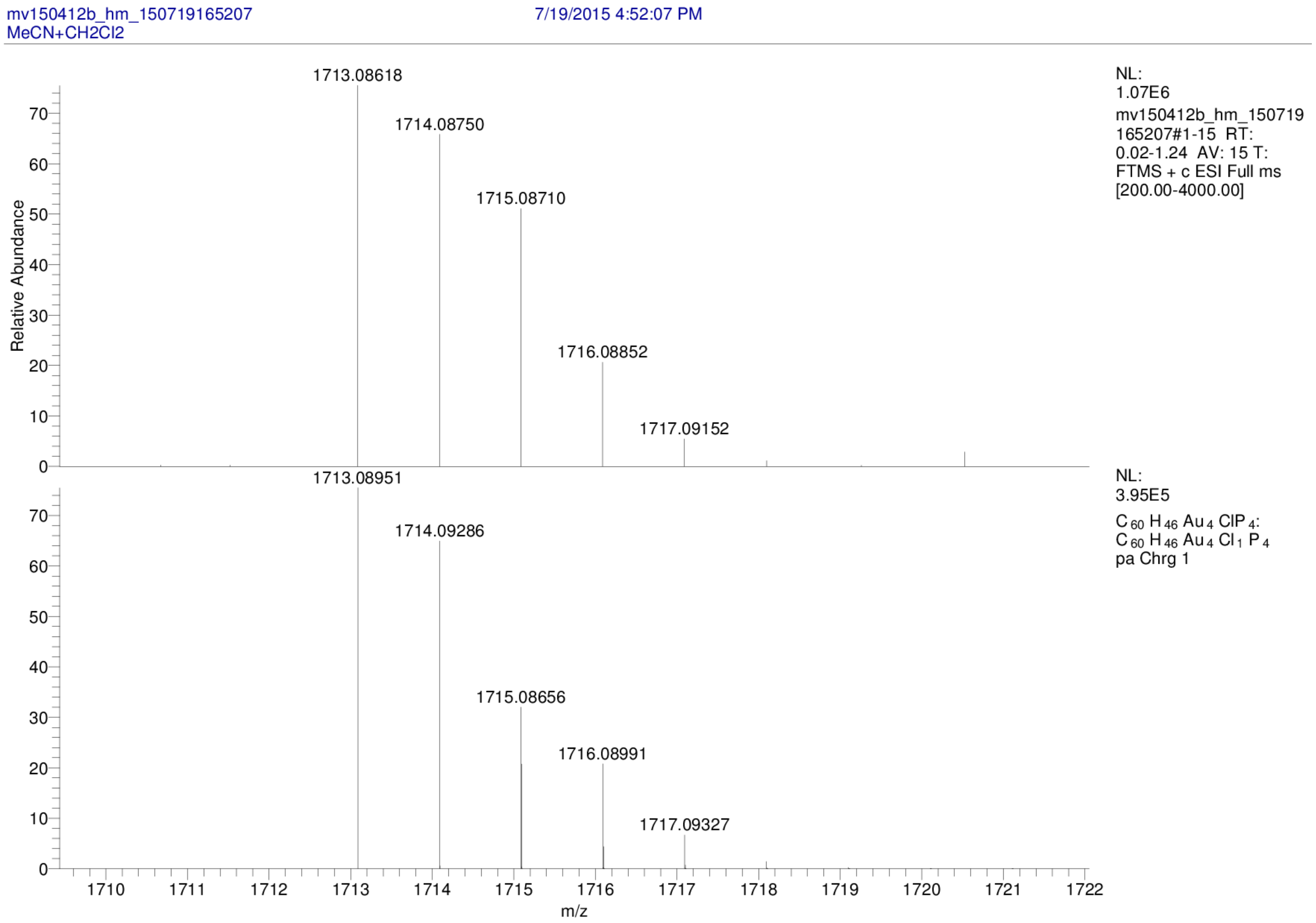

Figure S34: Measured (top) and calculated (bottom) isotopic pattern of $[M-Cl]^+$ of **3**.

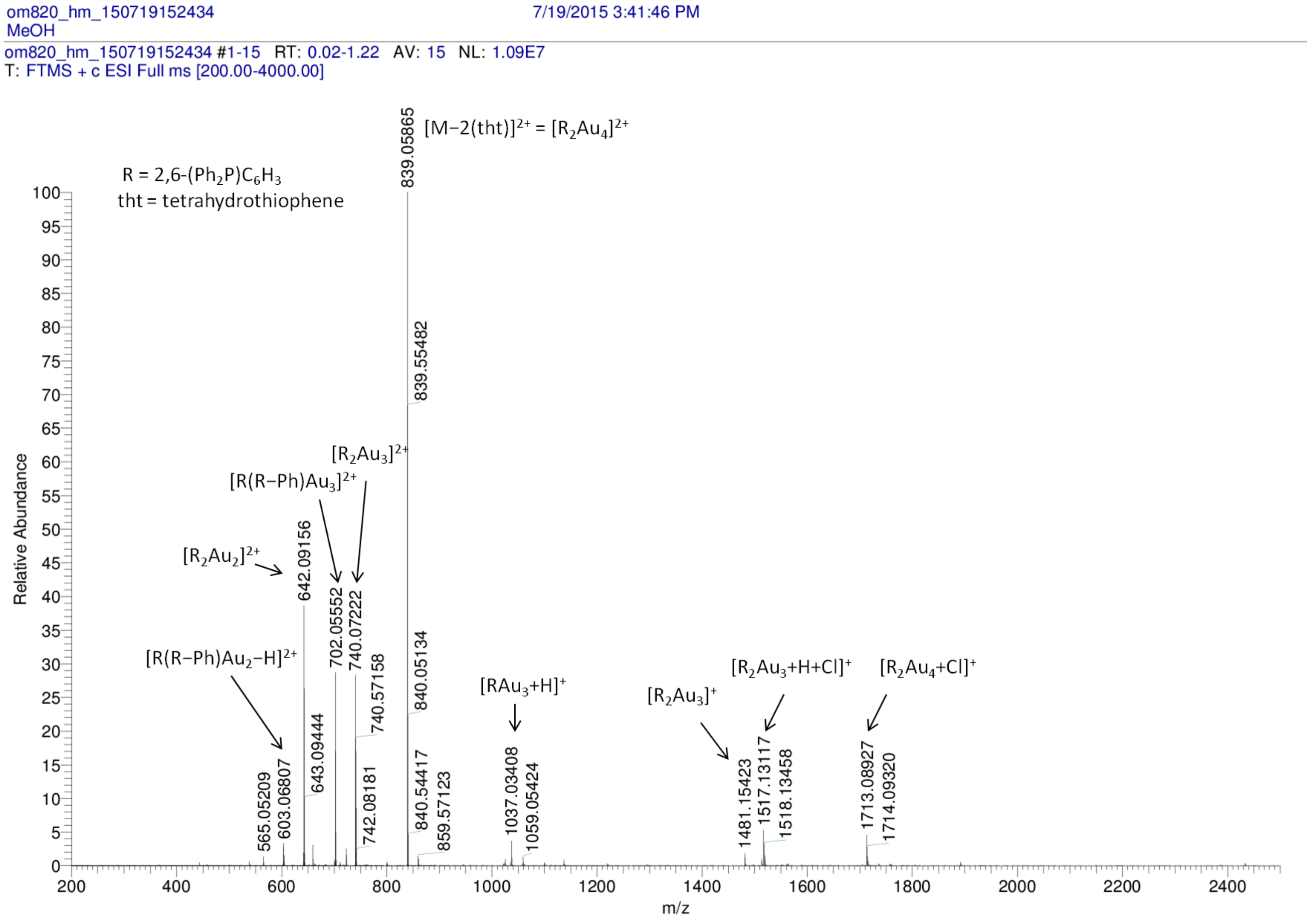

Figure S35: HRMS ESI(+) spectrum of **5**.

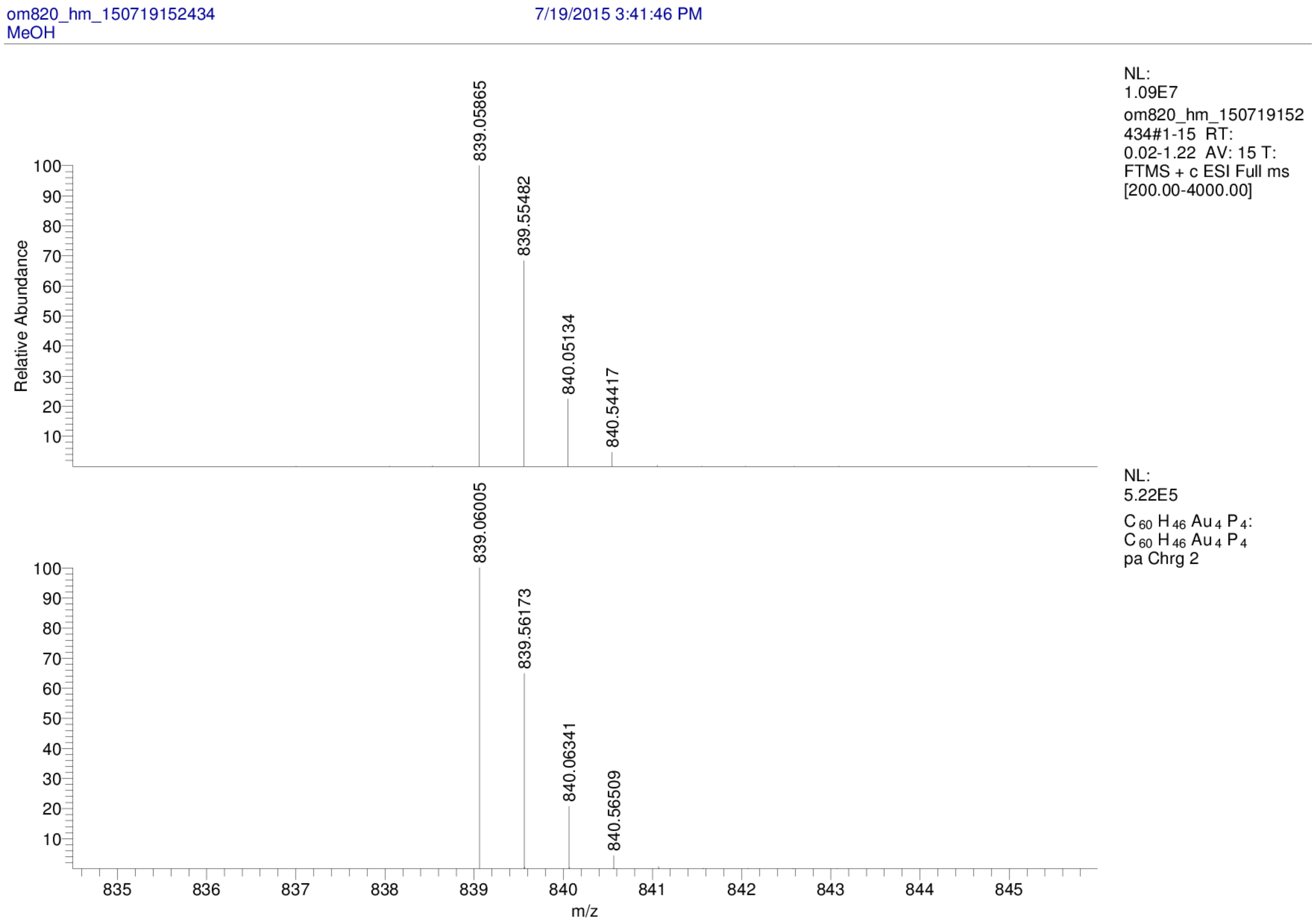

Figure S36: Measured (top) and calculated (bottom) isotopic pattern of $[M{-}2(tht)]^{2+}$ of **5**.

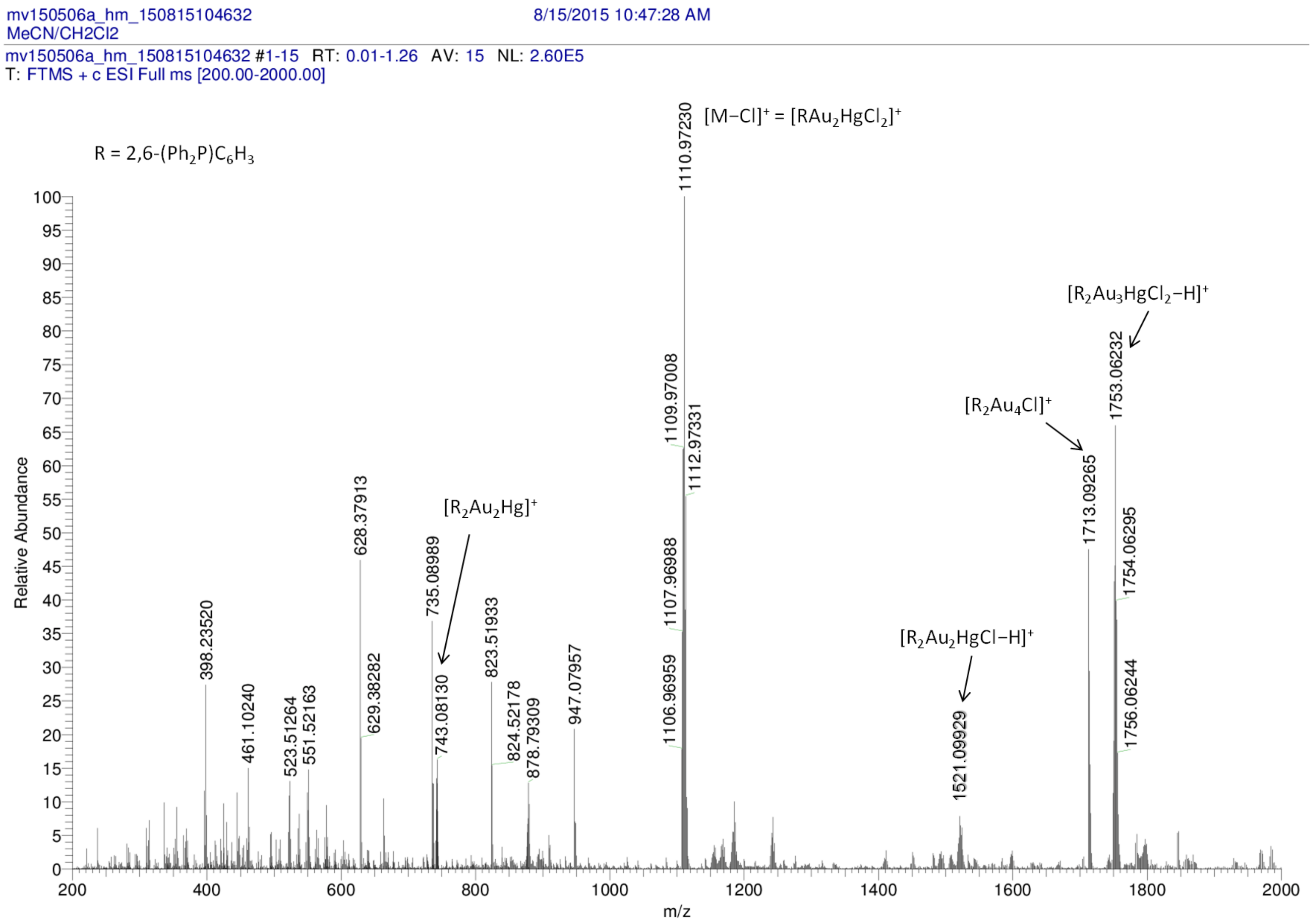

Figure S37: HRMS ESI(+) spectrum of **6**.

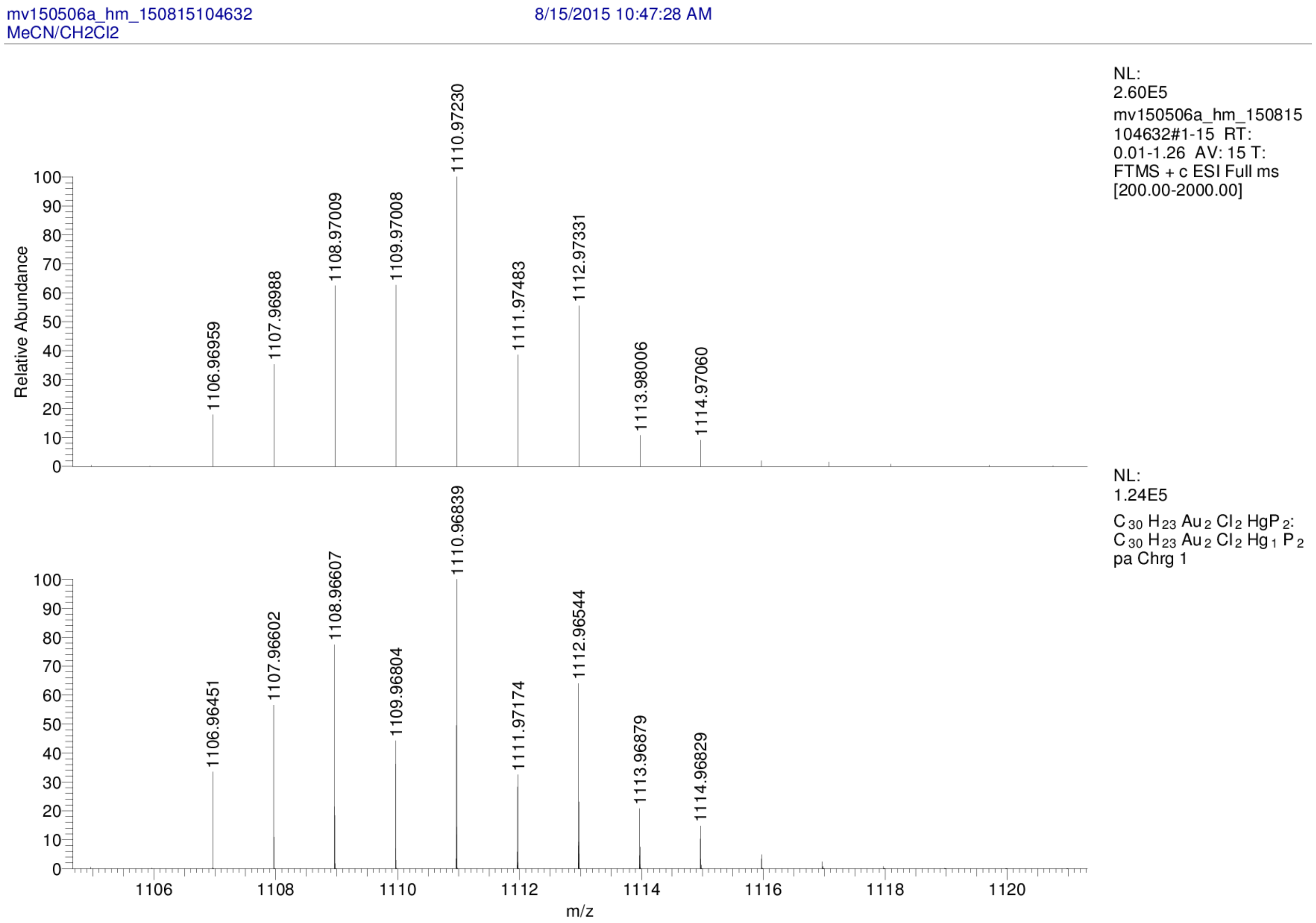

Figure S38: Measured (top) and calculated (bottom) isotopic pattern of $[M{-}Cl]^+$ of **6**

X-ray crystallography

Intensity data of **1**, **2**·CH_2Cl_2, **3**·CH_2Cl_2, **4**, **5**·3 CH_2Cl_2 and **6**·CH_2Cl_2 were collected at 100 K on a Bruker Venture D8 diffractometer with graphite-monochromated Mo-Kα (0.7107 Å) radiation. All structures were solved by direct methods and refined based on F^2 by use of the SHELX program package as implemented in WinGX.[25] All non-hydrogen atoms were refined using anisotropic displacement parameters. Hydrogen atoms attached to carbon atoms were included in geometrically calculated positions using a riding model. A heavy disordered $CHCl_3$ molecule co-crystallized with **4** was accounted using the SQUEZZE routine.[26] The hydrogen atom attached to disordered solvent molecules of **5** were not included into the refinement. Absolute structures were inferred by refinement of the Flack parameters for **3** (0.017(4)) and **6** (0.195(6)).[27] Crystal and refinement data are collected in Table 2. Figures were created using DIAMOND.[28] Crystallographic data (excluding structure factors) for the structural analyses have been deposited with the Cambridge Crystallographic Data Centre. Copies of this information may be obtained free of charge from The Director, CCDC, 12 Union Road, Cambridge CB2 1EZ, UK (Fax: +44-1223-336033; e-mail: deposit@ccdc.cam.ac.uk or http://www.ccdc.cam.ac.uk).

Table 2. Crystal data and structure refinement of **1**–**6**.

	**1**	**2**·CH_2Cl_2	**3**·CH_2Cl_2
Formula	$C_{33}H_{32}P_2Si$	$C_{34}H_{34}Au_2Cl_4P_2Si$	$C_{61}H_{48}Au_4Cl_4P_4$
Formula weight, g mol^{-1}	518.62	1068.38	1834.54
Crystal system	triclinic	monoclinic	monoclinic
Crystal size, mm	0.08 × 0.07 × 0.04	0.07 × 0.03 × 0.03	0.05 × 0.05 × 0.04
Space group	$P\bar{1}$	$P2_1/c$	$P2_1$
a, Å	7.9118(2)	20.3635(7)	12.9746(5)
b, Å	12.2181(2)	11.1977(4)	26.721(1)
c, Å	15.5661(3)	16.0413(5)	17.0685(6)
α, °	93.643(1)	90	90
β, °	103.912(1)	103.828(2)	106.002(1)
γ, °	97.000(1)	90	90
V, $Å^3$	1442.96(5)	3551.8(2)	5688.3(4)
Z	2	4	4
ρ_{calcd}, Mg m^{-3}	1.194	1.998	2.142
μ (Mo $K\alpha$), mm^{-1}	0.212	8.700	10.624
F(000)	548	2032	3432
θ range, deg	2.68 to 29.81	2.24 to 31.08	2.23 to 27.56
Index ranges	–11 ≤ h ≤ 11	–29 ≤ h ≤ 29	–16 ≤ h ≤ 16
	–17 ≤ k ≤ 17	–16 ≤ k ≤ 16	–34 ≤ k ≤ 34
	–21 ≤ l ≤ 21	–23 ≤ l ≤ 23	–22 ≤ l ≤ 22
No. of reflns collected	179053	201885	213933
Completeness to θ_{max}	99.7%	99.7%	99.8%
No. indep. Reflns	8463	11370	26177
No. obsd reflns with ($I>2\sigma(I)$)	7093	9964	24676
No. refined params	328	361	1315
GooF (F^2)	1.050	1.270	1.040
R_1 (F) ($I > 2\sigma(I)$)	0.0355	0.0389	0.0317
wR_2 (F^2) (all data)	0.0918	0.0962	0.0799
Largest diff peak/hole, e $Å^{-3}$	0.536 / –0.497	3.697 / –2.621	2.416 / –1.859
CCDC number	1569295	1569296	1569297

Table 2. cont.

**4**	**5**·3CH_2Cl_2	**6**·CH_2Cl_2
$C_{132}H_{86}Au_4B_2F_{48}P_4S_2$	$C_{135}H_{94}Au_4B_2Cl_6F_{48}P_4S_2$	$C_{31}H_{25}Au_2Cl_5HgP_2$
3581.49	3838.29	1231.22
monoclinic	triclinic	orthorhombic
0.05 × 0.05 × 0.05	0.04 × 0.04 × 0.02	0.10 × 0.10 × 0.06
C2/c	$P\bar{1}$	$Pca2_1$
24.366(1)	13.1360(4)	38.085(1)
25.321(1)	16.0949(6)	15.9410(6)
25.019(1)	17.5774(6)	11.0856(4)
90	71.438(1)	90
113.493(1)	80.658(1)	90
90	84.536(1)	90
14157(1)	3472.4(2)	6730.3(4)
4	1	8
1.736	1.835	2.430
4.374	4.518	13.763
7144	1856	4512
2.33 to 28.39	2.62 to 27.50	0.972 to 34.06
–32 ≤ h ≤ 32	–20 ≤ h ≤ 20	–58 ≤ h ≤ 58
–33 ≤ k ≤ 33	–25 ≤ k ≤ 25	–24 ≤ k ≤ 25
–33 ≤ l ≤ 33	–27 ≤ l ≤ 27	–17 ≤ l ≤ 17
236975	228147	537437
99.4%	99.7%	97.2%
17663	27354	26537
11761	22249	24371
865	162	729
1.044	1.060	1.191
0.0681	0.0553	0.0404
0.1771	0.1679	0.0941
2.457 / –1.746	7.324 / –7.433	6.176 / –4.595
1569298	1569299	1569300

Computational methodology

Density functional theory calculations were performed for the C-H corrected[14] XRD structures of compounds **3**–**6** at the B3PW91/6-311+G(2df,p)[29] level of theory with Gaussian09.[30] Additionally, effective core potentials (ECP28MDF)[31] and corresponding cc-pVTZ basis sets were applied to the Hg and Au atoms.[32] Subsequently, topological analysis of the electron density according to the Atoms-In-Molecules space-partitioning scheme[15] was performed using AIMALL,[33] whereas Electron-Localizability-Indicator (ELI-D) *iso*-surfaces[15] were generated by DGRID[34] (grid step size: 0.1 a.u.). NCI-grids (grid step size: 0.05 a.u.) are generated with NCIplot.[35] AIM bon paths motifs are displayed with AIMALL,[33] whereas NCI and ELI-D figures are displayed with MolIso.[36]

Table S1. Topological bond descriptors and delocalization index of prominent bonds of **3–6**

	**d** [Å]	**d_1/d**	**ρ_{bcp}** [eÅ^{-3}]	**$\nabla^2\rho_{bcp}$** [eÅ^{-5}]	**ε**	**G/ρ_{bcp}** [a.u.]	**H/ρ_{bcp}** [a.u.]	**δ(A,B)**
**3**								
Au1-Cl5	2.292	0.50	0.70	5.3	0.00	0.90	-0.37	1.01
Au2-Cl6	2.294	0.50	0.70	5.3	0.00	0.90	-0.37	1.00
Au1-P7	2.238	0.52	0.85	1.7	0.01	0.64	-0.50	1.02
Au2-P8	2.242	0.52	0.84	1.7	0.00	-0.64	-0.50	1.01
Au3-P9	2.314	0.51	0.75	2.1	0.01	0.64	-0.44	0.89
Au4-P10	2.292	0.51	0.78	2.0	0.01	0.64	-0.46	0.90
Au3-C11	2.068	0.54	0.90	4.0	0.04	0.78	-0.46	0.91
Au4-C17	2.040	0.54	0.95	4.3	0.04	0.80	-0.48	0.92
**4**								
Au1-P5	2.305	0.51	0.76	2.0	0.01	0.64	-0.45	0.89
Au2-P4	2.268	0.52	0.82	1.0	0.01	0.58	-0.49	0.95
Au40-P44	2.306	0.51	0.76	2.0	0.01	0.63	-0.45	0.89
Au41-P43	2.269	0.52	0.82	1.0	0.01	0.58	-0.49	0.95
Au2-S3	2.338	0.50	0.69	4.2	0.03	0.80	-0.38	0.87
Au41-S42	2.338	0.50	0.69	4.2	0.03	0.80	-0.38	0.87
Au1-C10	2.052	0.54	0.93	4.0	0.03	0.77	-0.48	0.92
Au40-C49	2.052	0.54	0.93	4.0	0.03	0.77	-0.48	0.92
**5**								
Au2-P4	2.319	0.51	0.75	1.7	0.01	0.60	-0.45	0.88
Au2-P44	2.312	0.52	0.76	1.6	0.01	0.60	-0.45	0.89
Au41-P5	2.313	0.52	0.76	1.6	0.01	0.60	-0.45	0.89
Au41-P43	2.318	0.51	0.75	1.7	0.01	0.60	-0.45	0.88
Au1-S3	2.339	0.50	0.69	4.3	0.02	0.82	-0.38	0.84
Au40-S42	2.339	0.50	0.69	4.3	0.02	0.82	-0.38	0.84
Au1-C10	2.024	0.55	0.99	3.7	0.04	0.76	-0.50	0.95
Au40-C49	2.025	0.55	0.98	3.7	0.04	0.76	-0.50	0.95
Au2-H116	2.952	0.63	0.06	0.5	0.17	0.59	0.08	0.04
Au41-H85	2.952	0.63	0.06	0.5	0.17	0.59	0.08	0.04
**6_{cis}**								
Hg1-C9	2.073	0.55	0.90	3.0	0.04	0.70	-0.46	0.88
Hg1-Cl6	2.308	0.50	0.70	4.6	0.01	0.83	-0.37	0.97
Au2-Cl4	2.282	0.50	0.72	5.3	0.00	0.90	-0.38	1.06
Au3-Cl5	2.295	0.50	0.70	5.1	0.00	0.88	-0.37	1.05
Au2-P7	2.236	0.52	0.85	1.6	0.01	0.64	-0.50	1.03
Au3-P8	2.238	0.52	0.85	1.7	0.01	0.64	-0.50	1.03
**6_{trans}**								

Hg1-C9	2.075	0.55	0.90	3.0	0.04	0.70	-0.46	0.88
Hg1-Cl6	2.302	0.50	0.71	4.7	0.00	0.84	-0.37	0.99
Au2-Cl4	2.285	0.50	0.72	5.2	0.00	0.89	-0.38	1.07
Au3-Cl5	2.284	0.50	0.72	5.2	0.00	0.88	-0.38	1.06
Au2-P7	2.239	0.52	0.84	1.8	0.00	0.64	-0.50	1.03
Au3-P8	2.232	0.52	0.86	1.7	0.01	0.64	-0.51	1.03

ρ_{bcp} – electron density, $\nabla^2\rho_{bcp}$ – Laplacian, d_1/d – ratio, ε – bond ellipticity, G/ρ_{bcp} and H/ρ_{bcp} – kinetic and total energy density over ρ_{bcp} ratios, δ – delocalization index. For labelling see Figure S39-42. In the main text, the labelling of the X-ray structure has been adopted for clarity.

Table S2. AIM atomic charges of **3**

atom	Q	atom	Q	atom	Q
Au1	0.07	C40	-0.04	H79	0.05
Au2	0.07	C41	-0.60	H80	0.05
Au3	0.02	C42	-0.04	H81	0.06
Au4	0.02	C43	-0.04	H82	0.06
Cl5	-0.55	C44	-0.03	H83	0.05
Cl6	-0.55	C45	-0.03	H84	0.05
P7	1.84	C46	-0.04	H85	0.05
P8	1.79	C47	-0.58	H86	0.07
P9	1.74	C48	-0.01	H87	0.07
P10	1.81	C49	-0.02	H88	0.06
C11	-0.13	C50	-0.03	H89	0.05
C12	-0.60	C51	-0.04	H90	0.05
C13	-0.05	C52	-0.04	H91	0.06
C14	-0.04	C53	-0.58	H92	0.08
C15	-0.04	C54	-0.03	H93	0.05
C16	-0.55	C55	-0.04	H94	0.04
C17	-0.14	C56	-0.03	H95	0.05
C18	-0.57	C57	-0.03	H96	0.07
C19	-0.04	C58	-0.02	H97	0.08
C20	-0.04	C59	-0.59	H98	0.05
C21	-0.04	C60	-0.01	H99	0.05
C22	-0.54	C61	-0.03	H100	0.04
C23	-0.59	C62	-0.03	H101	0.05
C24	-0.03	C63	-0.03	H102	0.07
C25	-0.03	C64	-0.03	H103	0.04
C26	-0.03	C65	-0.62	H104	0.05
C27	-0.04	C66	-0.04	H105	0.05
C28	-0.04	C67	-0.03	H106	0.06
C29	-0.58	C68	-0.03	H107	0.07
C30	-0.02	C69	-0.03	H108	0.05
C31	-0.03	C70	-0.02	H109	0.04
C32	-0.04	H71	0.06	H110	0.04
C33	-0.04	H72	0.05	H111	0.04
C34	-0.03	H73	0.06	H112	0.05
C35	-0.56	H74	0.06	H113	0.05
C36	-0.02	H75	0.05	H114	0.04
C37	-0.04	H76	0.06	H115	0.05
C38	-0.04	H77	0.07	H116	0.07
C39	-0.04	H78	0.06	sum	0.03

For labelling see Figure S39.

Table S3. AIM atomic charges of compound **4**

atom	Q	atom	Q	atom	Q	atom	Q
Au1	0.02	C37	-0.03	C73	-0.58	H109	0.06
Au2	0.00	C38	-0.03	C74	-0.03	H110	0.07
S3	0.06	C39	-0.04	C75	-0.05	H111	0.06
P4	1.83	Au40	0.02	C76	-0.03	H112	0.02
P5	1.76	Au41	0.00	C77	-0.02	H113	0.05
C6	-0.09	S42	0.06	C78	-0.04	H114	0.03
C7	0.03	P43	1.83	H79	0.07	H115	0.05
C8	0.03	P44	1.76	H80	0.06	H116	0.07
C9	-0.09	C45	-0.09	H81	0.02	H117	0.07
C10	-0.15	C46	0.03	H82	0.05	H118	0.08
C11	-0.59	C47	0.03	H83	0.03	H119	0.07
C12	-0.03	C48	-0.09	H84	0.05	H120	0.07
C13	-0.02	C49	-0.15	H85	0.07	H121	0.06
C14	-0.04	C50	-0.60	H86	0.07	H122	0.06
C15	-0.56	C51	-0.04	H87	0.08	H123	0.07
C16	-0.62	C52	-0.02	H88	0.07	H124	0.07
C17	-0.03	C53	-0.04	H89	0.07	H125	0.06
C18	-0.04	C54	-0.56	H90	0.06	H126	0.04
C19	-0.02	C55	-0.62	H91	0.06	H127	0.06
C20	-0.02	C56	-0.03	H92	0.07	H128	0.07
C21	-0.03	C57	-0.04	H93	0.07	H129	0.08
C22	-0.58	C58	-0.02	H94	0.06	H130	0.07
C23	-0.03	C59	-0.02	H95	0.04	H131	0.06
C24	-0.02	C60	-0.03	H96	0.06	H132	0.06
C25	-0.02	C61	-0.58	H97	0.07	H133	0.07
C26	-0.03	C62	-0.03	H98	0.08	H134	0.07
C27	-0.03	C63	-0.02	H99	0.07	H135	0.06
C28	-0.62	C64	-0.02	H100	0.06	H136	0.05
C29	-0.04	C65	-0.03	H101	0.06	H137	0.05
C30	-0.03	C66	-0.03	H102	0.07	H138	0.06
C31	-0.03	C67	-0.62	H103	0.07	H139	0.07
C32	-0.04	C68	-0.04	H104	0.06	H140	0.06
C33	-0.03	C69	-0.03	H105	0.05	sum	2.05
C34	-0.58	C70	-0.03	H106	0.05		
C35	-0.03	C71	-0.04	H107	0.06		
C36	-0.05	C72	-0.03	H108	0.07		

For labelling see Figure S40.

Table S4. AIM atomic charges of compound **5**

atom	Q	atom	Q	atom	Q	atom	Q
Au1	0.12	C37	-0.02	C73	-0.62	H109	0.07
Au2	-0.09	C38	-0.02	C74	-0.04	H110	0.08
S3	0.03	C39	-0.03	C75	-0.03	H111	0.06
P4	1.78	Au40	0.12	C76	-0.02	H112	0.04
P5	1.83	Au41	-0.09	C77	-0.02	H113	0.03
C6	-0.10	S42	0.03	C78	-0.03	H114	0.04
C7	0.02	P43	1.78	H79	0.08	H115	0.02
C8	0.04	P44	1.83	H80	0.06	H116	0.05
C9	-0.07	C45	-0.10	H81	0.04	H117	0.06
C10	-0.13	C46	0.02	H82	0.03	H118	0.08
C11	-0.59	C47	0.04	H83	0.03	H119	0.08
C12	-0.04	C48	-0.07	H84	0.02	H120	0.08
C13	-0.03	C49	-0.13	H85	0.05	H121	0.04
C14	-0.03	C50	-0.59	H86	0.06	H122	0.06
C15	-0.61	C51	-0.04	H87	0.08	H123	0.08
C16	-0.61	C52	-0.03	H88	0.08	H124	0.08
C17	-0.03	C53	-0.03	H89	0.08	H125	0.07
C18	-0.03	C54	-0.61	H90	0.04	H126	0.06
C19	-0.02	C55	-0.61	H91	0.06	H127	0.07
C20	-0.02	C56	-0.03	H92	0.08	H128	0.07
C21	-0.03	C57	-0.03	H93	0.08	H129	0.07
C22	-0.59	C58	-0.02	H94	0.07	H130	0.06
C23	-0.03	C59	-0.02	H95	0.06	H131	0.07
C24	-0.04	C60	-0.03	H96	0.07	H132	0.07
C25	-0.02	C61	-0.59	H97	0.07	H133	0.07
C26	-0.02	C62	-0.03	H98	0.07	H134	0.06
C27	-0.03	C63	-0.04	H99	0.06	H135	0.05
C28	-0.61	C64	-0.02	H100	0.07	H136	0.04
C29	-0.03	C65	-0.02	H101	0.07	H137	0.07
C30	-0.04	C66	-0.03	H102	0.07	H138	0.07
C31	-0.02	C67	-0.61	H103	0.06	H139	0.07
C32	-0.02	C68	-0.03	H104	0.05	H140	0.07
C33	-0.04	C69	-0.04	H105	0.04	sum	2.05
C34	-0.62	C70	-0.02	H106	0.07		
C35	-0.04	C71	-0.02	H107	0.07		
C36	-0.03	C72	-0.04	H108	0.07		

For labelling see Figure S41.

Table S5. AIM atomic charges of compounds $\mathbf{6}_{\mathbf{trans}}$ and $\mathbf{6}_{\mathbf{cis}}$

$\mathbf{6}_{\mathbf{trans}}$				$\mathbf{6}_{\mathbf{cis}}$			
atom	Q	atom	Q	atom	Q	atom	Q
Hg1	0.65	C32	-0.03	Hg1	0.64	C32	-0.04
Au2	0.07	C33	-0.60	Au2	0.07	C33	-0.60
Au3	0.07	C34	-0.03	Au3	0.07	C34	-0.04
Cl4	-0.53	C35	-0.02	Cl4	-0.52	C35	-0.04
Cl5	-0.53	C36	-0.03	Cl5	-0.51	C36	-0.02
Cl6	-0.47	C37	-0.02	Cl6	-0.47	C37	-0.02
P7	1.79	C38	-0.02	P7	1.87	C38	-0.02
P8	1.80	H39	0.07	P8	1.91	H39	0.08
C9	-0.15	H40	0.06	C9	-0.15	H40	0.06
C10	-0.57	H41	0.07	C10	-0.60	H41	0.07
C11	-0.03	H42	0.08	C11	-0.04	H42	0.07
C12	-0.03	H43	0.08	C12	-0.03	H43	0.06
C13	-0.03	H44	0.06	C13	-0.03	H44	0.06
C14	-0.52	H45	0.05	C14	-0.61	H45	0.06
C15	-0.58	H46	0.06	C15	-0.60	H46	0.06
C16	-0.01	H47	0.06	C16	-0.03	H47	0.05
C17	-0.03	H48	0.06	C17	-0.03	H48	0.04
C18	-0.04	H49	0.06	C18	-0.04	H49	0.06
C19	-0.02	H50	0.06	C19	-0.04	H50	0.05
C20	-0.04	H51	0.07	C20	-0.03	H51	0.08
C21	-0.60	H52	0.08	C21	-0.61	H52	0.08
C22	-0.03	H53	0.06	C22	-0.04	H53	0.06
C23	-0.04	H54	0.06	C23	-0.03	H54	0.05
C24	-0.03	H55	0.06	C24	-0.01	H55	0.05
C25	-0.03	H56	0.06	C25	-0.02	H56	0.06
C26	-0.03	H57	0.04	C26	-0.02	H57	0.06
C27	-0.62	H58	0.06	C27	-0.62	H58	0.05
C28	-0.03	H59	0.06	C28	-0.03	H59	0.05
C29	-0.03	H60	0.06	C29	-0.03	H60	0.06
C30	-0.03	H61	0.06	C30	-0.03	H61	0.07
C31	-0.03	sum	0.02	C31	-0.03	sum	0.01

For labelling see Figure S42.

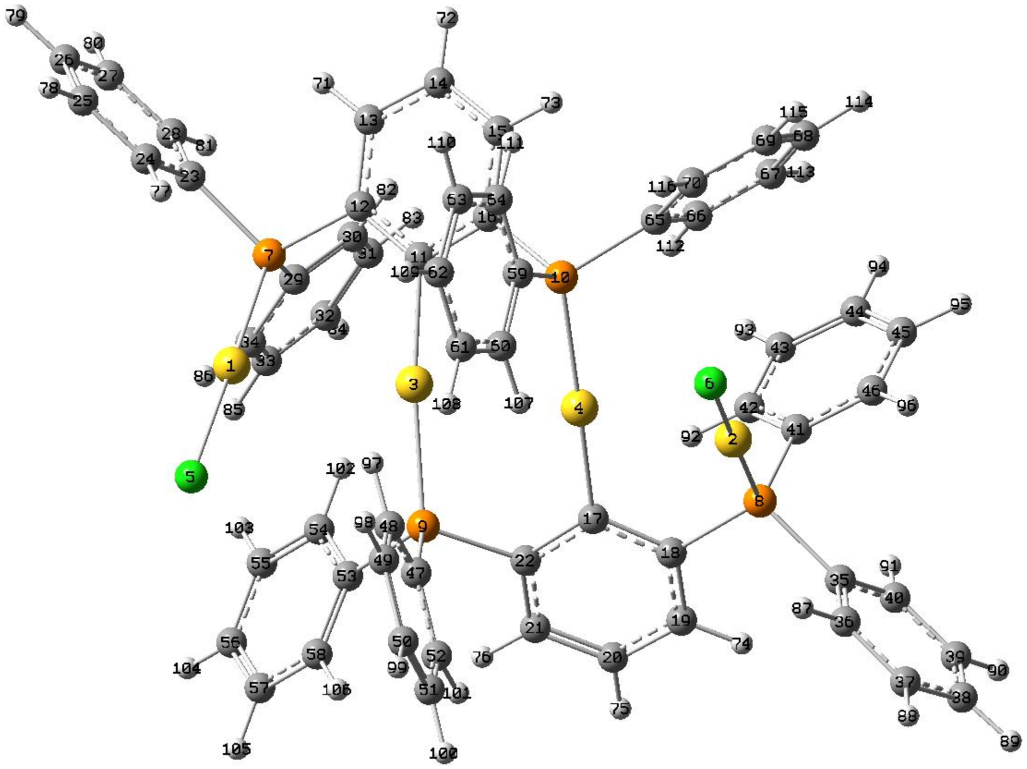

Figure S39: Labeling schemes in calculated **3**.

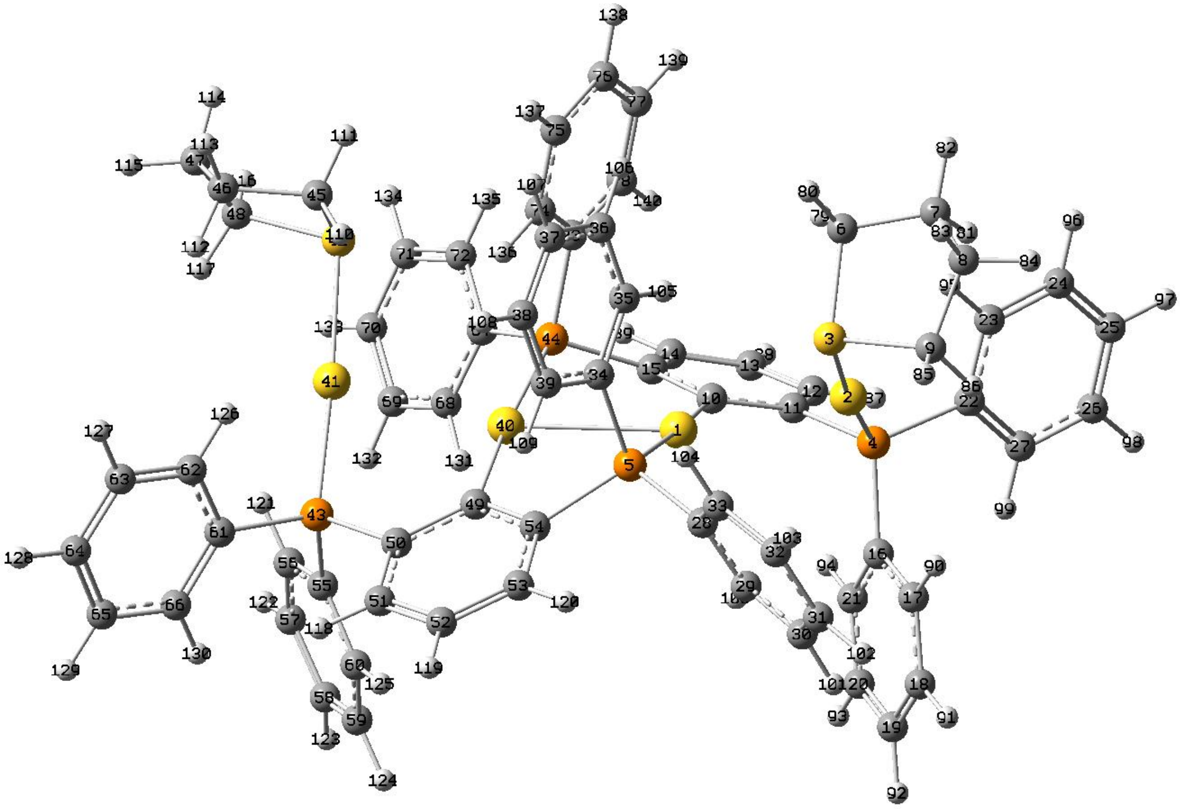

Figure S40. Labeling schemes in calculated **4**.

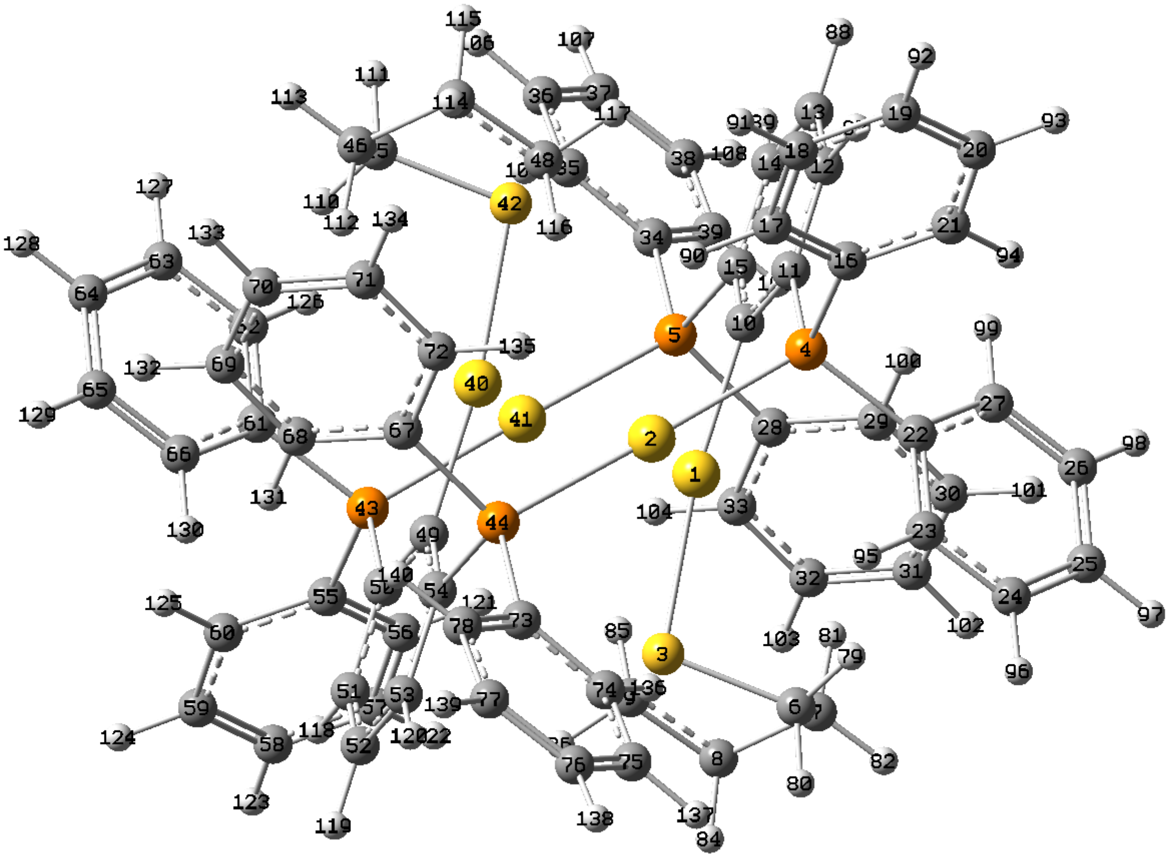

Figure S41. Labeling schemes in calculated **5**.

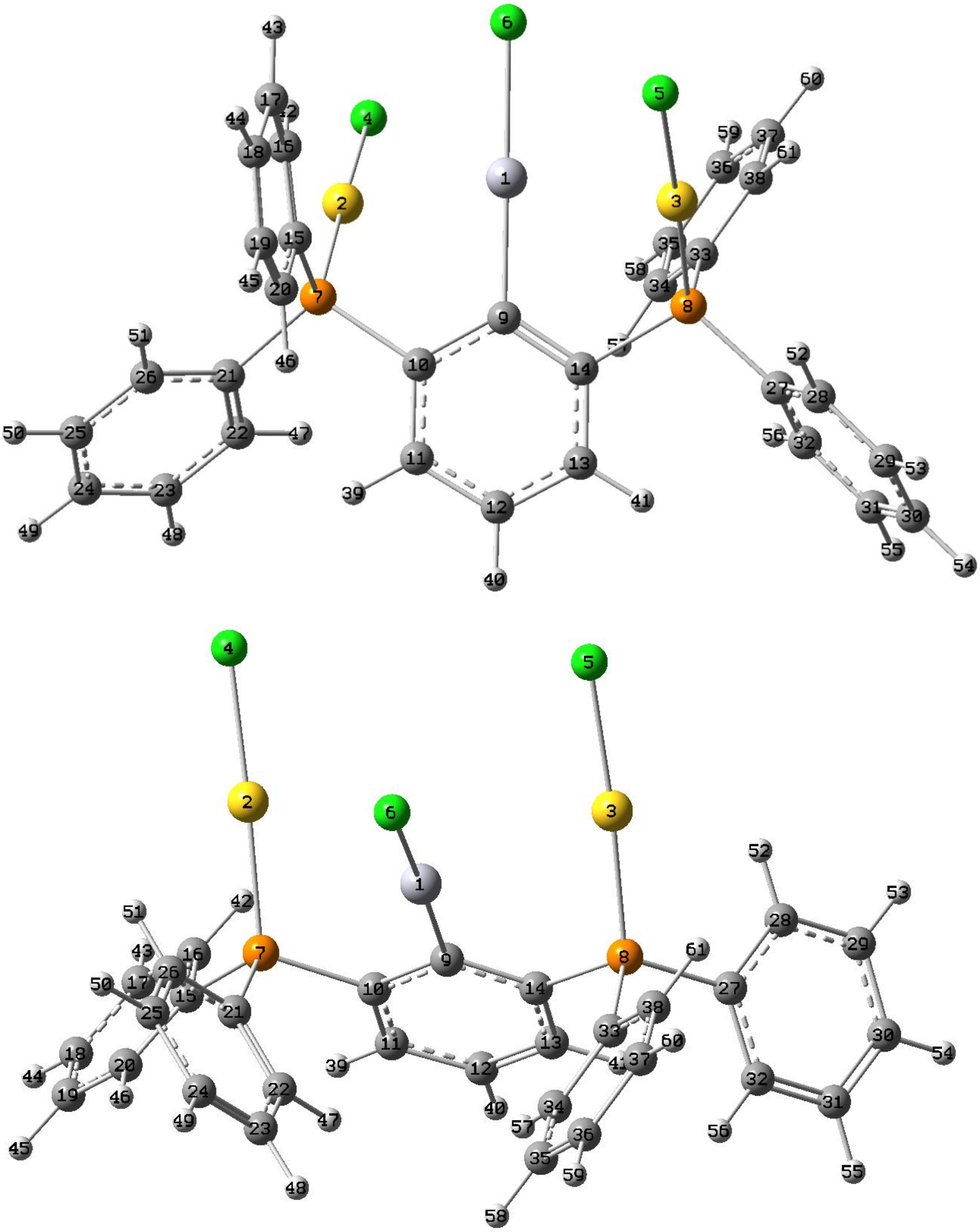

Figure S42. Labeling schemes in calculated $\mathbf{6}_{\mathbf{trans}}$ and $\mathbf{6}_{\mathbf{cis}}$.

References

[20] (a) T. D. Yarwood, A. J. Waring, P. Le. Coe, *J. Fluor. Chem.*, **1996**, *78*, 113–119. (b) Bennetau, F. Rajarison, J. Dunoguès, P. Babin, *Tetrahedron*,**1993**, *49*, 10843–10854.

[21] M. Schlosser, C. Heiss, *Eur. J. Org. Chem.* **2003**, 4618–4624

[22] (a) N. A. Yakelis, R. G. Bergman, *Organometallics* **2005**, *24*, 3579–3581. (b) M. Brookhart, B. Grant, A. F. Volpe Jr., *Organometallics* **1992**, *11*, 3920–3922.

[23] G. R. Fulmer, A. J. M. Miller, N. H. Sherden, H. E. Gottlieb, A. Nudelman, B. M. Stoltz, J. E. Bercaw, K. I. Goldberg, *Organometallics* **2010**, *29*, 2176–2179

[24] Qual Browser Thermo Xcalibur, version 2.1.0 SP1.1160; Thermo Fischer Scientific Inc.: Waltham, MA 02454, 2011.

[25] (a) G. M. Sheldrick *Acta Cryst*. **2008**, *A64*, 112-122. (b) G.M. Sheldrick *Acta Cryst*. **2015**, *C71*, 3-8. (c) L. J. Farrugia, *J. Appl. Crystallogr*. **1999**, *32*, 837–838.

[26] A. Spek J. Appl. Cryst. **2003**, 36, 7–13.

[27] H. D. Flack *Acta Cryst*. **1983**, *A39*, 876–881.

[28] K. Brandenburg, DIAMOND version 3.2i, Crystal Impact GbR, Bonn Germany **2012**.

[29] (a) J. P. Perdew, J. A. Chevary, S. H. Vosko, K. A. Jackson, M. R. Pederson, D. J. Singh, C. Fiolhais *Phys. Rev. B* **1992**, *46*, 6671–6687. (b) A. D. Becke, *J. Chem. Phys.* **1993**, *98*, 5648–5652.

[30] M. J. Frisch, G. W. Trucks, H. B. Schlegel, G. E. Scuseria, M. A. Robb, J. R. Cheeseman, G. Scalmani, V. Barone, B. Mennucci, G. A. Petersson, H. Nakatsuji, M. Caricato, X. Li, H. P. Hratchian, A. F. Izmaylov, J. Bloino, G. Zheng, J. L. Sonnenberg, M. Hada, M. Ehara, K. Toyota, R. Fukuda, J. Hasegawa, M. Ishida, T. Nakajima, Y. Honda, O. Kitao, H. Nakai, T. Vreven, J. A. Montgomery Jr., J. E. Peralta, F. Ogliaro, M. Bearpark, J. J. Heyd, E. Brothers, K. N. Kudin, V. N. Staroverov, R. Kobayashi, J. Normand, K. Raghavachari, A. Rendell, J. C. Burant, S. S. Iyengar, J. Tomasi, M. Cossi, N. Rega, J. M. Millam, M. Klene, J. E. Knox, J. B. Cross, V. Bakken, C. Adamo, J. Jaramillo, R. Gomperts, R. E. Stratmann, O. Yazyev, A. J. Austin, R. Cammi, C. Pomelli, J. W. Ochterski, R. L. Martin, K. Morokuma, V. G. Zakrzewski,

G. A. Voth, P. Salvador, J. J. Dannenberg, S. Dapprich, A.D. Daniels, O. Farkas, J. B. Foresman, J. V. Ortiz, J. Cioslowski, D. J. Fox, *Gaussian09*, revision B.01; Gaussian, Inc.: Wallingford, CT, **2010**.

[31] Figgen, D; Rauhut, G.; Dolg, M.; Stoll, H. *Chem. Phys.*, **2005**, *311*, 227–244.

[32] Peterson, K. A.; Puzzarini, C. *Theor. Chem. Acc.*, **2005**, *114*, 283–296.

[33] Keith, T.A. *AIMall* (Version 13.11.04, Professional) T. K. Gristmill Software, Overland Park KS, USA, **2009** (http://aim.tkgristmill.com).

[34] Kohout, M *DGRID*, version 4.5, Radebeul, **2009**.

[35] J. Contreras-García, E. R. Johnson, S. Keinan, R. Chaudret, J.-P. Piquemal, D. N. Beratan, W. Yang *J. Chem. Theor. Comp.*, **2011**, *7*, 625–632.

[36] C. B. Hübschle, P. Luger *J. Appl. Crystallogr*. **2006**, *39*, 901–904.
